# Discovery of first-in-class inhibitors of ASH1L histone methyltransferase with anti-leukemic activity

**DOI:** 10.1038/s41467-021-23152-6

**Published:** 2021-05-14

**Authors:** David S. Rogawski, Jing Deng, Hao Li, Hongzhi Miao, Dmitry Borkin, Trupta Purohit, Jiho Song, Jennifer Chase, Shuangjiang Li, Juliano Ndoj, Szymon Klossowski, EunGi Kim, Fengbiao Mao, Bo Zhou, James Ropa, Marta Z. Krotoska, Zhuang Jin, Patricia Ernst, Xiaomin Feng, Gang Huang, Kenichi Nishioka, Samantha Kelly, Miao He, Bo Wen, Duxin Sun, Andrew Muntean, Yali Dou, Ivan Maillard, Tomasz Cierpicki, Jolanta Grembecka

**Affiliations:** 1 Department of Pathology, University of Michigan, Ann Arbor, MI USA; 2 Life Sciences Institute, University of Michigan, Ann Arbor, MI USA; 3 Department of Microbiology and Immunology, Indiana University School of Medicine, Indianapolis, IN USA; 4 Department of Pediatrics, University of Colorado Denver, Anschutz Medical Campus, Aurora, CO USA; 5 Department of Pathology and Laboratory Medicine, Cincinnati Children’s Hospital, Cincinnati, OH USA; 6 Department of Internal Medicine Musashimurayama Hospital, Enoki 1-1-5, Musashimurayama, Tokyo, Japan; 7 Division of Hematology-Oncology, Perelman School of Medicine, University of Pennsylvania, Philadelphia, PA USA; 8 College of Pharmacy, University of Michigan, Ann Arbor, MI USA

**Keywords:** Small molecules, Structure-based drug design

## Abstract

ASH1L histone methyltransferase plays a crucial role in the pathogenesis of different diseases, including acute leukemia. While ASH1L represents an attractive drug target, developing ASH1L inhibitors is challenging, as the catalytic SET domain adapts an inactive conformation with autoinhibitory loop blocking the access to the active site. Here, by applying fragment-based screening followed by medicinal chemistry and a structure-based design, we developed first-in-class small molecule inhibitors of the ASH1L SET domain. The crystal structures of ASH1L-inhibitor complexes reveal compound binding to the autoinhibitory loop region in the SET domain. When tested in MLL leukemia models, our lead compound, AS-99, blocks cell proliferation, induces apoptosis and differentiation, downregulates MLL fusion target genes, and reduces the leukemia burden in vivo. This work validates the ASH1L SET domain as a druggable target and provides a chemical probe to further study the biological functions of ASH1L as well as to develop therapeutic agents.

## Abstract (web summary)

The histone methyltransferase ASH1L plays a role in various diseases, including cancer, and has been validated as a therapeutic target; however, no inhibitors of ASH1L have been reported. Here the authors present small molecule inhibitors of ASH1L and demonstrate their on-target activity in leukemia cells and a mouse model of leukemia.

## Introduction

Chromatin modulation through covalent modifications of histones, including histone lysine methylation, plays an important role in the regulation of gene expression both in normal and pathological states^1^. Histone methyltransferases (HMTs) involved in methylation of histone 3 on lysine 36 (H3K36) play an important role as regulators of cell growth, differentiation, stemness, and DNA repair pathways^2,3^. The ASH1L (absent, small, or homeotic-like 1) protein is a histone lysine methyltransferase that catalyzes the mono- and dimethylation of H3K36, representing an activating mark on chromatin^2,4–6^. At the molecular level, ASH1L is a large, ~3000 amino acid protein that contains a catalytic SET domain responsible for the transfer of a methyl group from the S-adenosyl methionine (SAM) cofactor to the lysine substrate^2,7^. In addition to the SET domain, ASH1L contains three chromatin reader domains, such as bromodomain, PHD and BAH, as well as a long unstructured region at the N-terminus of the protein^2^.

In mammals, ASH1L shares a conserved functional relationship with another trithorax-group protein Mixed Lineage Leukemia 1 (MLL1)^8^. Both ASH1L and MLL1 positively regulate the expression of *HOX* genes and support the self-renewal potential of hematopoietic stem cells^4,9–12^. In addition, ASH1L plays a crucial role in the pathogenesis of acute leukemia with chromosomal translocations of the *MLL1* gene (also known as *KMT2A*)^9^, which occur in 5–10% of acute leukemia patients and lead to very poor prognosis (only ~35% 5-year survival)^13–15^. The knockdown of *ASH1L* in MLL leukemia cells leads to growth arrest, apoptosis, differentiation, and downregulation of *HOXA9* genes that are essential to leukemogenesis, and abrogates development of MLL leukemia in vivo^9^. At the molecular level, ASH1L maintains the H3K36me2 mark, which enables the recruitment of the MLL1 protein complex to chromatin to activate target gene expression in leukemia cells^9^. In addition, the overexpression of ASH1L was found in thyroid, breast, and liver cancers, and is associated with enhanced cancer cell growth and aggressive disease^16–18^. Thus, the importance of ASH1L in acute leukemia and other cancers strongly supports its high relevance as an attractive therapeutic target in oncology. However, to date no small molecule inhibitors of ASH1L have been reported.

Previous studies have shown that the catalytic SET domain of ASH1L is involved in the regulation of *Hoxa* genes in mouse embryonic stem (ES) cells^5^, suggesting that the histone methyltransferase activity of ASH1L is important for its gene activating function. Here, we demonstrate the relevance of the catalytic SET domain of ASH1L in the leukemic transformation mediated by MLL fusion proteins, supporting the rationale for inhibitor development. Then, we present the development of the first-in-class small molecule inhibitors of the catalytic SET domain of ASH1L with nanomolar activity and high selectivity to ASH1L. We also report crystal structures of the ASH1L-inhibitor complexes, revealing compounds binding to the autoinhibitory loop region in the SET domain. When tested in MLL leukemia cells, our lead compound, **AS-99**, blocked proliferation, induced apoptosis and differentiation, downregulated MLL fusion target genes, and reduced the leukemia burden in a mouse model of MLL leukemia. Overall, this work reveals that the ASH1L SET domain represents a druggable target and provides a valuable chemical probe to study the biological functions of ASH1L and a molecular scaffold to develop therapeutic agents targeting ASH1L.

## Results

### ASH1L SET domain is required for transformation by MLL fusions

**Fig. 1:**
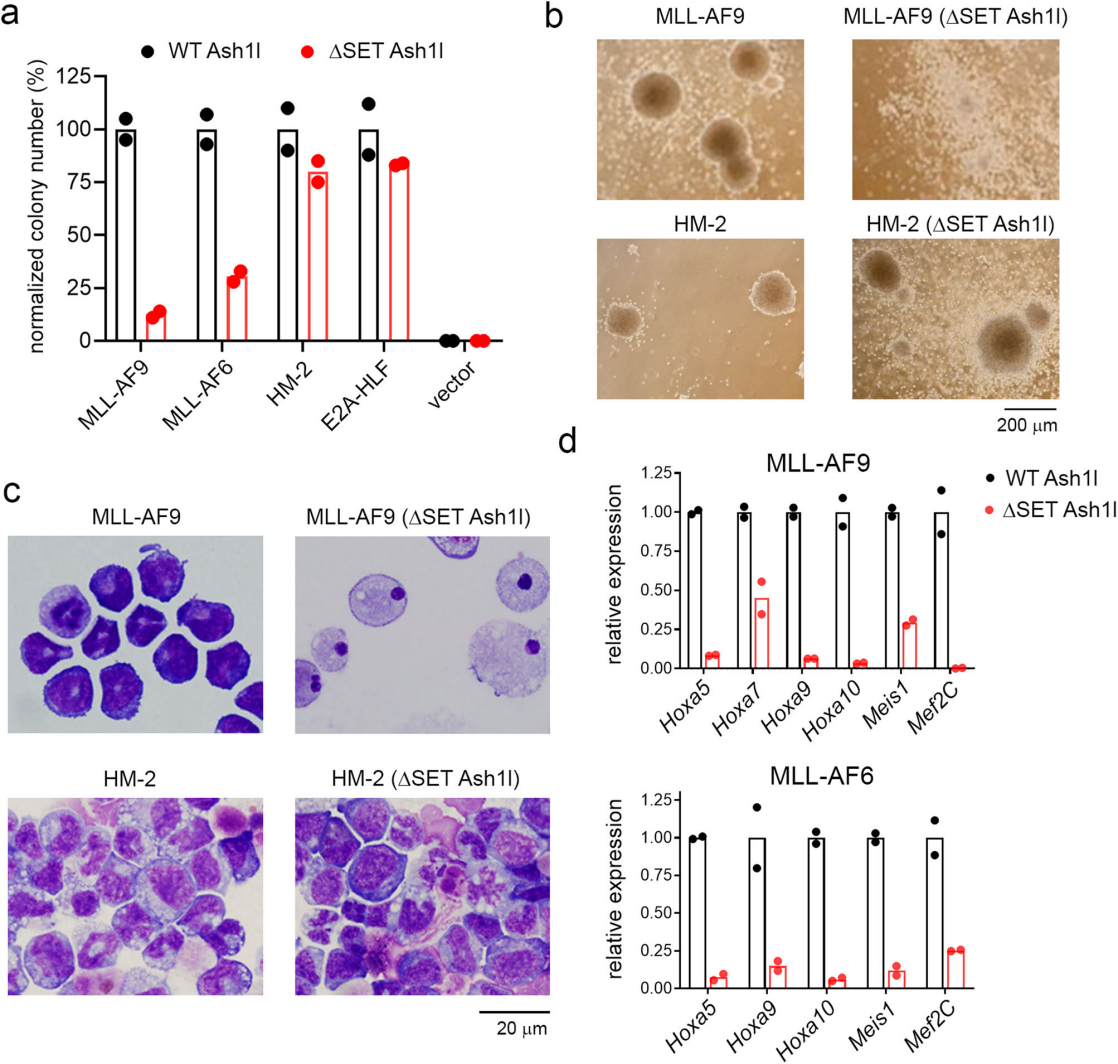
SET domain of ASH1L is essential for efficient transformation by MLL fusion oncogenes. **a** Normalized colony counts from the round four of colony-forming assay performed in mouse bone marrow (BM) progenitor cells derived from the WT or **∆**SET Ash1l mice and transduced with *MLL-AF9*, *MLL-AF6*, *HOXA9/MEIS1* (HM-2), *E2A-HLF* or vector alone (MSCV). *n* = 2. **b**, **c** Representative pictures of colonies (**b**) or Wright-Giemsa stained cytospins (**c**) from the round four of colony assay performed in bone marrow cells derived from WT or **∆**SET Ash1l mice and transduced with *MLL-AF9* or *HOXA9/MEIS1* (HM-2). The experiment was repeated twice with similar results. **d** Quantitative RT-PCR performed in BM cells derived from WT or **∆**SET Ash1l mice and transduced with *MLL-AF9* or *MLL-AF6*. Gene expression was normalized to *Gapdh* and gene expression changes in **∆**SET Ash1l cells were referenced to the corresponding values in the WT Ash1l background. Data represent two independent experiments each performed in duplicates.

Previous studies have revealed an important role of ASH1L in leukemogenesis mediated by MLL fusion proteins^9^, however, the requirement for the catalytic SET domain in this process was unknown. To address this question, we took advantage of a mouse model with an in-frame deletion of the Ash1l SET domain (ΔSET Ash1l)^5^. Importantly, these mice remain viable, and we isolated bone marrow cells from these mice and from the wild type (WT) control mice and transformed them with two *MLL* fusion oncogenes, *MLL-AF9* and *MLL-AF6*, as well as with control oncogenes, *HOXA9/MEIS1* (HM-2) and *E2A-HLF*, the later acts through non-*HOX* pathways^19^. By performing a colony forming assay with serial replating, we found that the transduction of WT Ash1l bone marrow cells with any of these oncogenes resulted in colony formation after several rounds of replating, which is indicative of transformation (Fig. 1a and Supplementary Fig. 1a). In contrast, both *MLL-AF9* and *MLL-AF6* oncogenes failed to efficiently transform bone marrow cells isolated from the ΔSET Ash1l mice as manifested by a substantially reduced (>75%) colony number compared to the WT Ash1l background (Fig. 1a and Supplementary Fig. 1a). Furthermore, the remaining colonies from ΔSET Ash1l cells transduced with the *MLL* fusion oncogenes were significantly smaller and more diffused when compared to colonies from the WT Ash1l background (Fig. 1b and Supplementary Fig. 1b). The cells derived from these colonies showed an increased differentiating phenotype and a higher level of myeloid differentiation markers (Ly-6G and Gr-1), supporting the inefficient transformation of ΔSET Ash1l bone marrow cells by MLL fusion proteins (Fig. 1c and Supplementary Fig. 1d). In contrast, the loss of the Ash1l SET domain did not impact transformation mediated by the *HOXA9/MEIS1* or *E2A-HLF* oncogenes, as the number and size of the colonies and cell morphology did not differ between WT or ΔSET Ash1l bone marrow cells (Fig. 1a,b,c and Supplementary Fig. 1a,c). Finally, we performed gene expression studies in *MLL-AF9* and *MLL-AF6* transformed bone marrow cells and found that genes highly relevant to MLL leukemia (*Hoxa5*, *Hoxa9*, *Hoxa10*, *Meis1*, and *Mef2C*) were expressed at much lower levels in the ΔSET Ash1l versus WT Ash1l cells, (Fig. 1d). In contrast, the expression level of these genes was not markedly different in the bone marrow cells transformed with control oncogenes *HOXA9/MEIS1* or *E2A-HLF* in the ΔSET Ash1l versus WT Ash1l cells, Supplementary Fig. 1e. Overall, these results validate the SET domain of ASH1L as essential for MLL-fusion protein mediated leukemogenesis, supporting the rationale for developing small molecules blocking the histone methyltransferase activity of ASH1L.

### Identification of a fragment hit that binds to the ASH1L SET domain

**Fig. 2:**
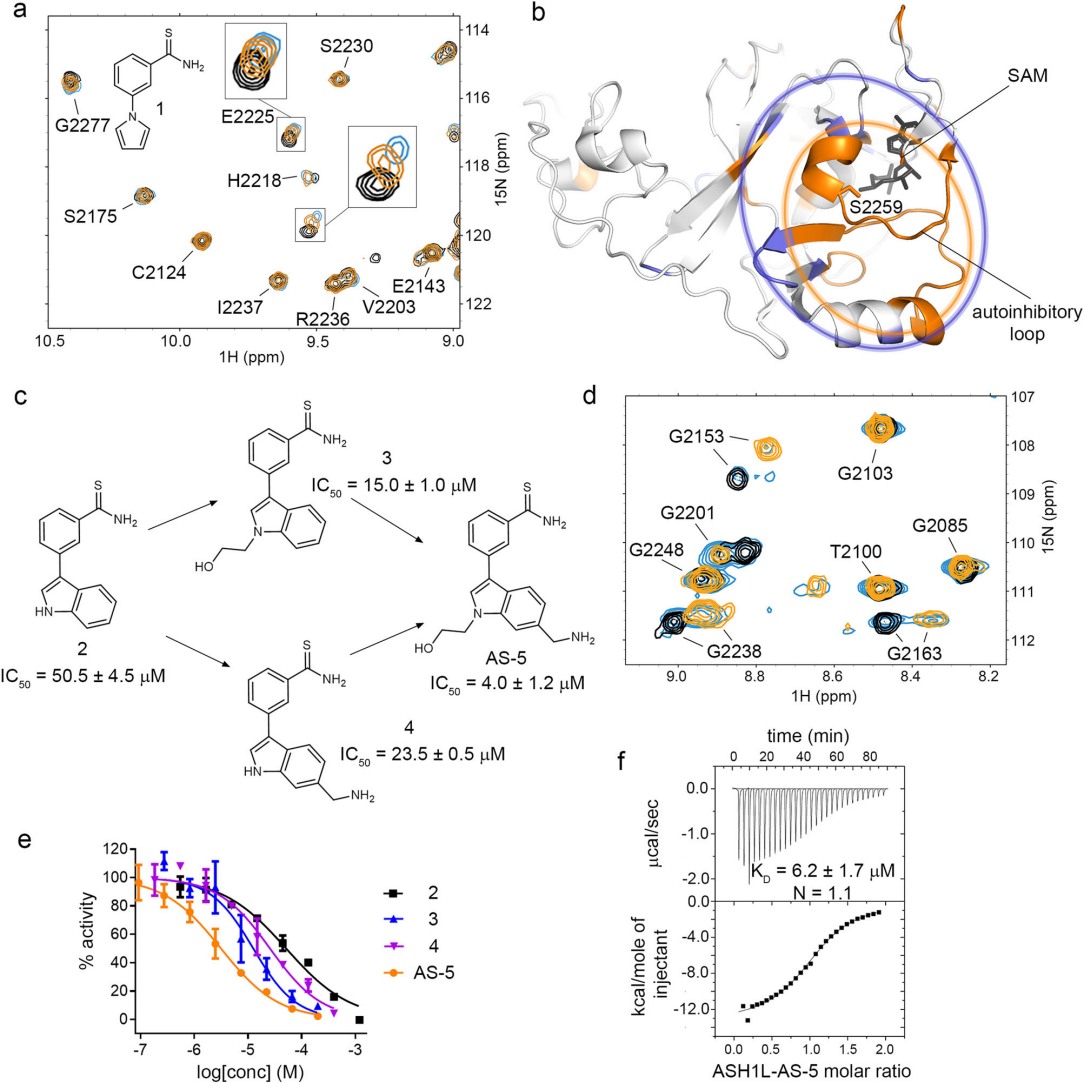
Development and characterization of ASH1L inhibitors. **a** Structure of the fragment hit, compound **1**, and its binding to ASH1L SET domain. Superposition of the ^1^H-^15^N TROSY-HSQC spectra of 100 µM ASH1L with 5% DMSO (black) with 500 µM (orange) or 1 mM (blue) of compound **1**. **b** Chemical shift perturbations upon binding of 1 to ASH1L mapped on the crystal structure of ASH1L SET domain (PDB code 4YNM). Residues experiencing chemical shift perturbations calculated as ${\triangle }_{{\rm{HN}}}=\sqrt{({{\rm{\delta }}}_{{\rm{HN}}}^{2}+{{\rm{\delta }}}_{{\rm{N}}}^{2})}$ larger than 15 Hz are colored and encircled in violet. Residues unobserved or unassigned are colored and encircled in orange. **c** Chemical structures and activities for selected ASH1L inhibitors. IC_50_ values represent mean ± s.d. from two independent experiments. **d** Superposition of the ^1^H-^15^N TROSY-HSQC spectra of 100 µM ASH1L with 5% DMSO (black) and with 500 µM (orange) or 100 µM (blue) of **AS-5**. **e** Titration curves from the HMT assay for ASH1L with compounds presented in panel (**c**) mean ± s.d. Representative curves are shown from two independent experiments, each performed in duplicates. **f** Binding isotherm from the ITC experiment performed for the binding of **AS-5** to ASH1L. Data are mean ± s.d. from two independent experiments. A representative binding isotherm is shown. N represents stoichiometry of binding.

To identify ASH1L inhibitors we conducted an NMR-based screening of ~1600 fragment-like compounds from an in-house library and identified a thioamide containing compound **1** that binds to the ASH1L SET domain, as validated by chemical shift perturbations on the ^15^N-^1^H TROSY-HSQC spectrum (Fig. 2a and Supplementary Fig. 2a). In NMR titration experiments, we observed linear chemical shift perturbations up to 1 mM of **1**, indicating weak binding affinity with K_d_ above 1 mM (Fig. 2a). To identify the binding site of **1**, we mapped chemical shift perturbations onto the crystal structure of the ASH1L SET domain (Fig. 2b). As we previously reported, due to conformational heterogeneity we could not observe signals on the NMR spectra of ASH1L corresponding to the autoinhibitory loop region^7^. However, we found that the residues most perturbed upon binding of **1** to ASH1L correspond to the amino acids surrounding the autoinhibitory loop (Fig. 2b). To confirm whether **1** binds to this region on ASH1L, we mutated S2259 to methionine to introduce steric hindrance in the autoinhibitory loop region (Supplementary Fig. 2b). Indeed, the NMR spectra of the ASH1L S2259M showed no chemical shift perturbations in the presence of **1**, validating no binding and supporting that our fragment hit binds to the autoinhibitory loop region (Supplementary Fig. 2b,c).

### Chemical optimization of a fragment hit 1

To improve the potency of **1**, we undertook medicinal chemistry optimization and found that the replacement of the pyrrole with an indole, which resulted in compound **2** (Fig. 2c), substantially improved binding affinity, as evidenced by more extensive chemical shift perturbations on the NMR spectra (Fig. 2d). To test whether **2** inhibits catalytic activity of ASH1L, we used an in vitro enzymatic assay with nucleosomes as the substrate^7^ and found that **2** blocked ASH1L activity with an IC_50_ value of 50 μM (Fig. 2c,e). Interestingly, despite the moderate inhibitory activity, we observed slow exchange kinetics on the ^15^N-^1^H TROSY-HSQC spectra upon binding of **2** to the SET domain of ASH1L, indicating a slow off-rate for the dissociation of the inhibitor from the protein (Fig. 2d).

Next, we substituted the indole nitrogen in compound **2** with a hydroxyethyl group, yielding compound **3** with three-fold improved inhibitory activity (IC_50_ = 15 μM, Fig. 2c, e). In addition, the substitution of the indole at position six with the aminomethyl group resulted in **4** with a two-fold improved inhibitory activity (IC_50_ = 23 µM) over **2**. By combining both substituents we synthesized compound **5** (**AS-5**), which exhibited low micromolar activity (IC_50_ = 4.0 μM, Fig. 2c, e). We then used isothermal titration calorimetry (ITC) to quantify the binding affinity of **AS-5** to ASH1L SET and obtained 1:1 stoichiometry and K_d_ of 6.2 μM, which is consistent with the inhibitory activity for this compound (Fig. 2f). Overall, these efforts led to over 200-fold improvement of the activity for **AS-5** versus the original fragment hit.

### AS-5 binds to the autoinhibitory loop region of ASH1L SET

**Fig. 3:**
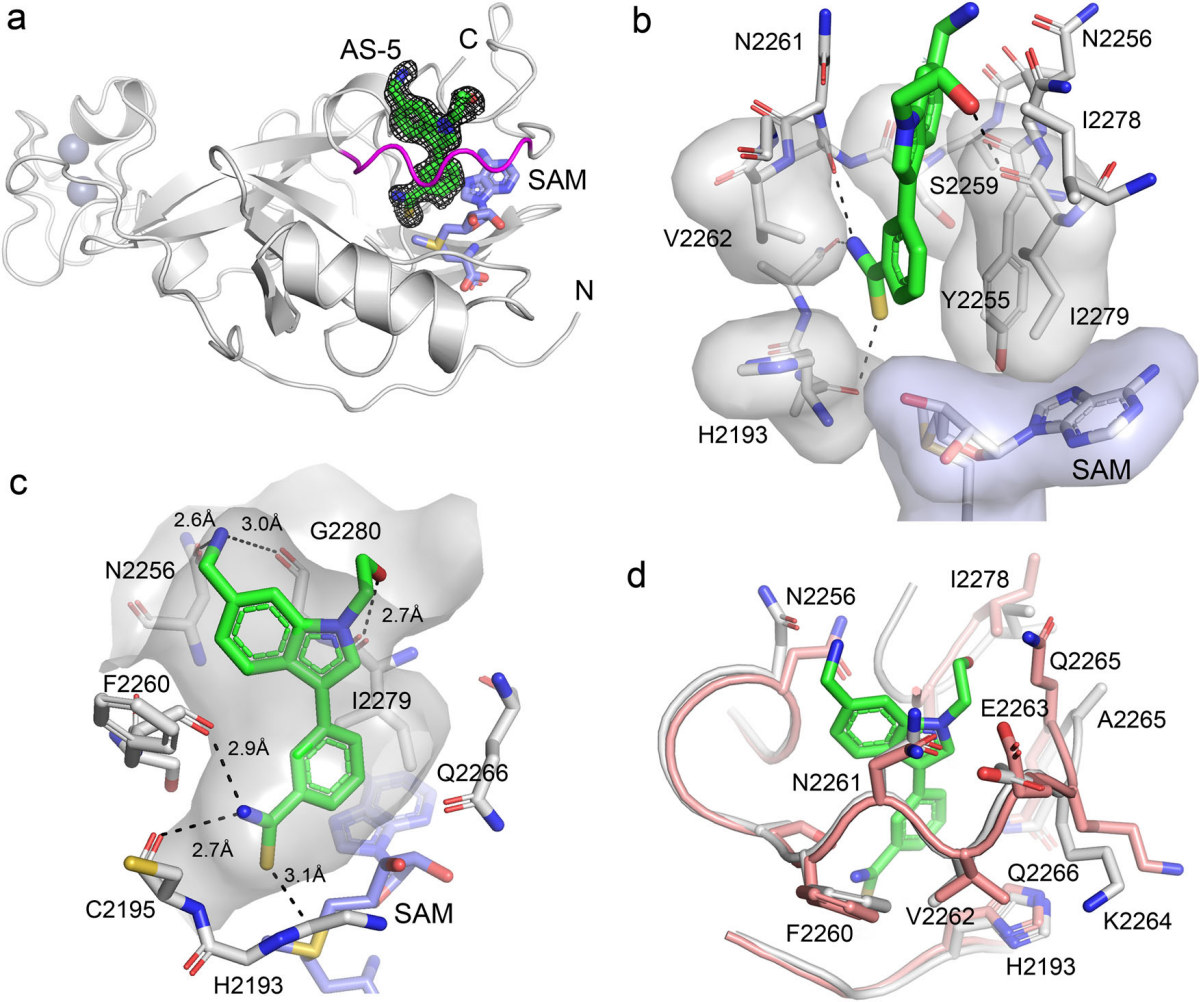
Crystal structure of ASH1L SET in complex with AS-5. **a** Overall structure of ASH1L-AS-5 complex with 2Fo-Fc electron density map for **AS-5** contoured at the 1σ level. Protein is shown as ribbon with autoinhibitory loop in magenta and **AS-5** shown in sticks with green carbons. **b** ASH1L residues involved in hydrophobic contacts with **AS-5** shown in sticks and transparent surfaces (gray) and SAM is shown in sticks with the blue surface. Hydrogen bonds and chalcogen bonds are shown as dashed lines. Color-coding: **AS-5** carbons (green), protein and SAM carbons (gray), oxygens (red), nitrogen (blue), sulfur (yellow). **c** Details of internal pocket in ASH1L (shown as semi-transparent surface) and binding mode of **AS-5** (shown in sticks with green carbons). Selected residues in the ASH1L binding site are shown as sticks with color coding is as in panel (**c**). Hydrogen bonds and chalcogen bonds are shown as dashed lines with distances in Å. **d** Superposition of ASH1L structure (PDB code 4YNM with salmon carbons) on the structure of ASH1L-**AS-5** complex (protein with gray carbons and **AS-5** with green carbons).

To further characterize the binding of ASH1L inhibitors we crystallized the ASH1L SET domain in complex with **AS-5**. Crystals of the wild-type SET domain diffracted to a low resolution (~3 Å), precluding detailed structural analysis. In contrast, co-crystallization of **AS-5** with ASH1L SET Q2265A mutant^7^ yielded well diffracting crystals (1.69 Å resolution), enabling structure determination of the complex, which showed a well-defined electron density map of **AS-5** bound to ASH1L (Fig. 3a, Supplementary Fig. 3a and Supplementary Table 1). The ASH1L-**AS-5** structure revealed that the compound binds to a predominantly buried site formed by residues from the autoinhibitory loop and the C-terminus of the SET domain and is restricted at the bottom by the SAM co-factor (Fig. 3a). This site is lined by the hydrophobic portions of the side chains of H2193, Y2255, S2259, V2262, and I2279, the polar groups from the side chains of N2256, N2261, and Q2266, and the SAM co-factor (Fig. 3b-d). An interesting feature of the binding site on ASH1L is the presence of multiple backbone carbonyls from S2259, C2159, H2193, N2256, F2260, K2264, and G2280, most of which are involved in the interactions with **AS-5** (Fig. 3b, c). Overall, the crystal structure shows high shape complementarity of **AS-5** to the binding site in the ASH1L SET domain (Fig. 3b, c). The thioamide group of **AS-5** is deeply inserted into the pocket in ASH1L and is engaged in a network of high-quality interactions, including two hydrogen bonds with carbonyl groups of C2195 and F2260, Fig. 3b, c. In addition, the thioamide group is located at a close distance (O ˑˑˑ S = C distance of 3.1 Å) and has favorable geometry (O ˑˑˑ S = C angle of 146°) to the carbonyl group of H2193, suggesting the formation of the chalcogen bond^20^ (Fig. 3b, c). The hydroxyethyl substituent on the indole nitrogen forms a hydrogen bond with the backbone carbonyl of I2279, while the aminomethyl group is involved in hydrogen bonds with the backbone carbonyl of G2280 and with the side chain of N2256 (Fig. 3b, c).

We have previously reported that the autoinhibitory loop in ASH1L experiences conformational dynamics that regulate its enzymatic activity^7^. The crystal structure of the ASH1L SET domain in the absence of the ligand reveals the presence of a relatively narrow crevice between the autoinhibitory and C-terminal loops, with the autoinhibitory loop blocking access to the active site. The binding of **AS-5** to this region induces relatively small conformational changes (Fig. 3d and Supplementary Fig. 3b) and suggests that **AS-5** exploits conformational heterogeneity in the ASH1L SET domain rather than inducing large conformational changes upon binding to the protein.

### Structure-based design and development of potent ASH1L inhibitors

**Fig. 4:**
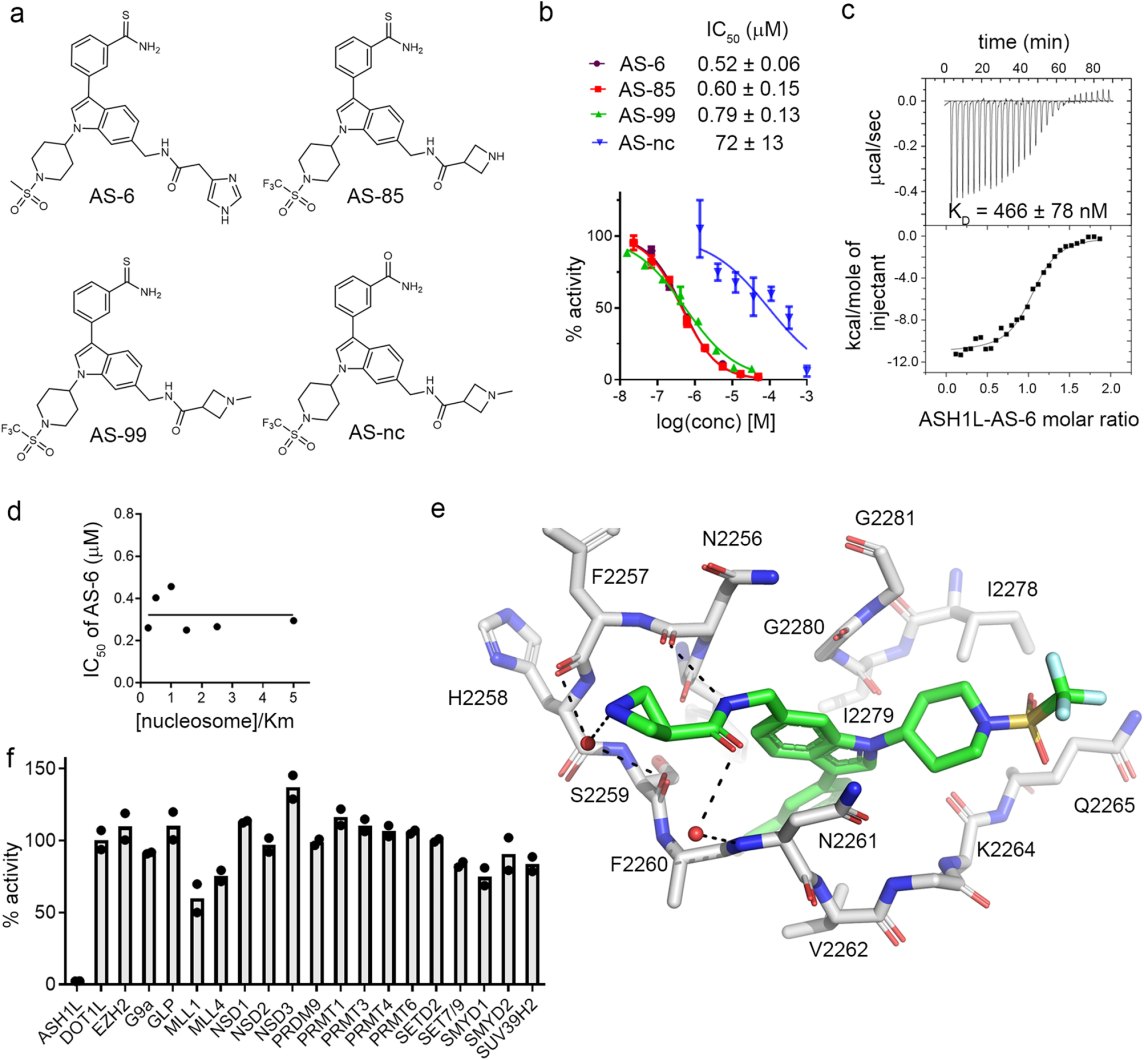
Structure-based optimization of ASH1L inhibitors. **a** Chemical structures of ASH1L inhibitors developed using structure-based design. **b** Titration curves and IC_50_ values from the HMT assay. Data are mean ± s.d. from two independent experiments. **c** Binding isotherm from the ITC experiment performed for the binding of **AS-6** to ASH1L. Data are mean ± s.d. from two independent experiments. A representative binding isotherm is shown. **d** Mechanistic studies from HMT assay with the IC_50_ values for **AS-6** measured at various nucleosome concentration demonstrating non-competitive inhibition with nucleosome. **e** Crystal structure of ASH1L-**AS-85** complex determined at 1.69 Å resolution. Selected ASH1L residues (gray carbons) and AS-85 (green carbons) are shown in sticks. Hydrogen bonds are shown as dashed lines. **f** Selectivity of **AS-99** tested at 50 µM concentration against a panel of histone methyltransferases. Data represent two independent experiments each performed in duplicates.

Analysis of the crystal structure of the ASH1L-**AS-5** complex suggests that the protein could accommodate larger substituents at the indole nitrogen and showed that the aminomethyl group in **AS-5** points towards the C-terminus of an α-helix in the SET domain. Thus, we replaced the hydroxyethyl group at indole nitrogen of **AS-5** with 1-(methylsulfonyl)piperidine substituent and introduced a positively charged imidazole moiety at position six of indole to afford favorable interactions with the helix dipole, resulting in compound **6** (**AS-6**), (Fig. 4a). Importantly, compound **AS-6** demonstrated ~10-fold improved inhibitory activity over **AS-5**, resulting in an IC_50_ value of 0.52 µM and K_d_ of 466 nM for binding to ASH1L as measured by ITC (Fig. 4b, c).

The binding mode of the ASH1L inhibitors that we developed suggests a requirement for the SAM co-factor for their effective binding (Fig. 3a–c). To further investigate the mechanism of inhibition and evaluate whether **AS-6** is competitive with the nucleosome substrate, we tested the activity of **AS-6** as a function of nucleosome concentration. Interestingly, **AS-6** demonstrates the ASH1L inhibition that is non-competitive with the nucleosome, suggesting that the inhibitor does not directly prevent substrate binding to the SET domain (Fig. 4d). Such a model remains in agreement with the reports emphasizing the requirement for ASH1L motifs outside of the SET domain to achieve catalytic activity, likely by facilitating nucleosome binding^7,21^.

Despite potent in vitro activity, **AS-6** demonstrated a very limited effect on the proliferation of the MLL leukemia cells (GI_50_ ~ 50 µM) (Supplementary Fig. 4), possibly due to poor cell permeability associated with the higher polarity of this compound (clogP = 1.6, tPSA = 120), Supplementary Table 2. Thus, we designed two further analogs of **AS-6** by introducing 1-(trifluoromethylsulfonyl) piperidine moiety at the indole nitrogen and azetidine or methyl-azetidine rings at position six of the indole, yielding **AS-85** (clogP = 3.4, tPSA = 108) and **AS-99** (clogP = 4.1, tPSA = 99), respectively (Fig. 4a). Importantly, both compounds are potent ASH1L inhibitors (IC_50_ of 0.60 and 0.79 µM for **AS-85** and **AS-99**, respectively), and strongly bind to the ASH1L SET domain, with the K_d_ values of 0.78 and 0.89 µM, respectively, Fig. 4a, b and Supplementary Fig. 5a. Both analogs, **AS-85** and **AS-99** have been designed to increase cLogP and decrease tPSA when compared to **AS-6**, with the aim to improve their cell permeability. We also noticed that ligand efficiency (LE)^22^ for these compounds slightly dropped during the lead optimization process (from ~0.32 to ~0.24), which was associated with the need to improve the activity, cell permeability and drug-like properties, Supplementary Table 2.

To understand how optimized inhibitors bind to ASH1L, we determined the crystal structure of the wild-type ASH1L in complex with **AS-85** (1.69 Å resolution) and found well defined electron density of the ligand (Fig. 4e and Supplementary Fig. 5b). Overall, the binding mode of **AS-85** is very similar to **AS-5**, and the common core structure occupies the same site on ASH1L (Fig. 3 and Fig. 4e). The piperidine moiety at indole nitrogen fits into a ledge formed by the side chains of E2263, Q2265, and I2278 as well as the backbone of the C-terminal residues G2280 and G2281 (Fig. 4e). The positively charged azetidine ring of **AS-85** forms favorable contacts with the helix dipole from the short helical fragment encompassing residues from N2254 to H2258 and a water-mediated hydrogen bond with F2257 (Fig. 4e). In addition, the amide group in the linker extending from the indole ring at position six is involved in a hydrogen bond with the backbone carbonyl of N2256 and a water-mediated hydrogen bond with N2261 (Fig. 4e).

An intriguing feature of this class of ASH1L inhibitors is the presence of a thioamide group, which forms a unique network of interactions, including the chalcogen bond between the sulfur in thioamide and the backbone carbonyl of H2193 (Fig. 3b and Supplementary Fig. 5b). To assess whether sulfur in the thioamide is required for high binding affinity we replaced the thioamide in **AS-99** with an amide group, yielding **AS-nc** (Fig. 4a). Surprisingly, the replacement of sulfur with oxygen resulted in ~100-fold reduced inhibitory activity, yielding an IC_50_ of 72 µM for **AS-nc** (Fig. 4b), emphasizing the high relevance of the chalcogen bond involving the thioamide group in this class of ASH1L inhibitors. The modeling demonstrates a relatively close distance between the amide oxygen in **AS-nc** and the carbonyl group of H2193, which likely results in repulsive interactions, significantly lowering the activity of **AS-nc** versus **AS-99** (Supplementary Fig. 5c). Since **AS-nc** has poor in vitro activity and a very similar structure to **AS-99**, we used this compound as a negative control for cell-based studies.

Structural analysis revealed that the ASH1L inhibitors reported here bind to the pocket formed by the autoinhibitory loop representing a characteristic feature of H3K36 histone methyltransferases, including the NSD family and SETD2^23^. To evaluate the selectivity of these compounds towards ASH1L, we tested **AS-99** against a panel of 20 histone methyltransferases, including NSD1, NSD2, NSD3, and SETD2. Importantly, no significant inhibition was observed at 50 µM of **AS-99** on any of the tested HMTs, indicating over 100-fold selectivity towards ASH1L (Fig. 4f). Furthermore, **AS-99** did not show substantial inhibition in a panel of representative kinases (Supplementary Fig. 6). Overall, **AS-99** is a sub-micromolar ASH1L inhibitor with a favorable selectivity profile and no substantial off-target effects.

### AS-99 shows on-target activity in MLL leukemia cells

**Fig. 5:**
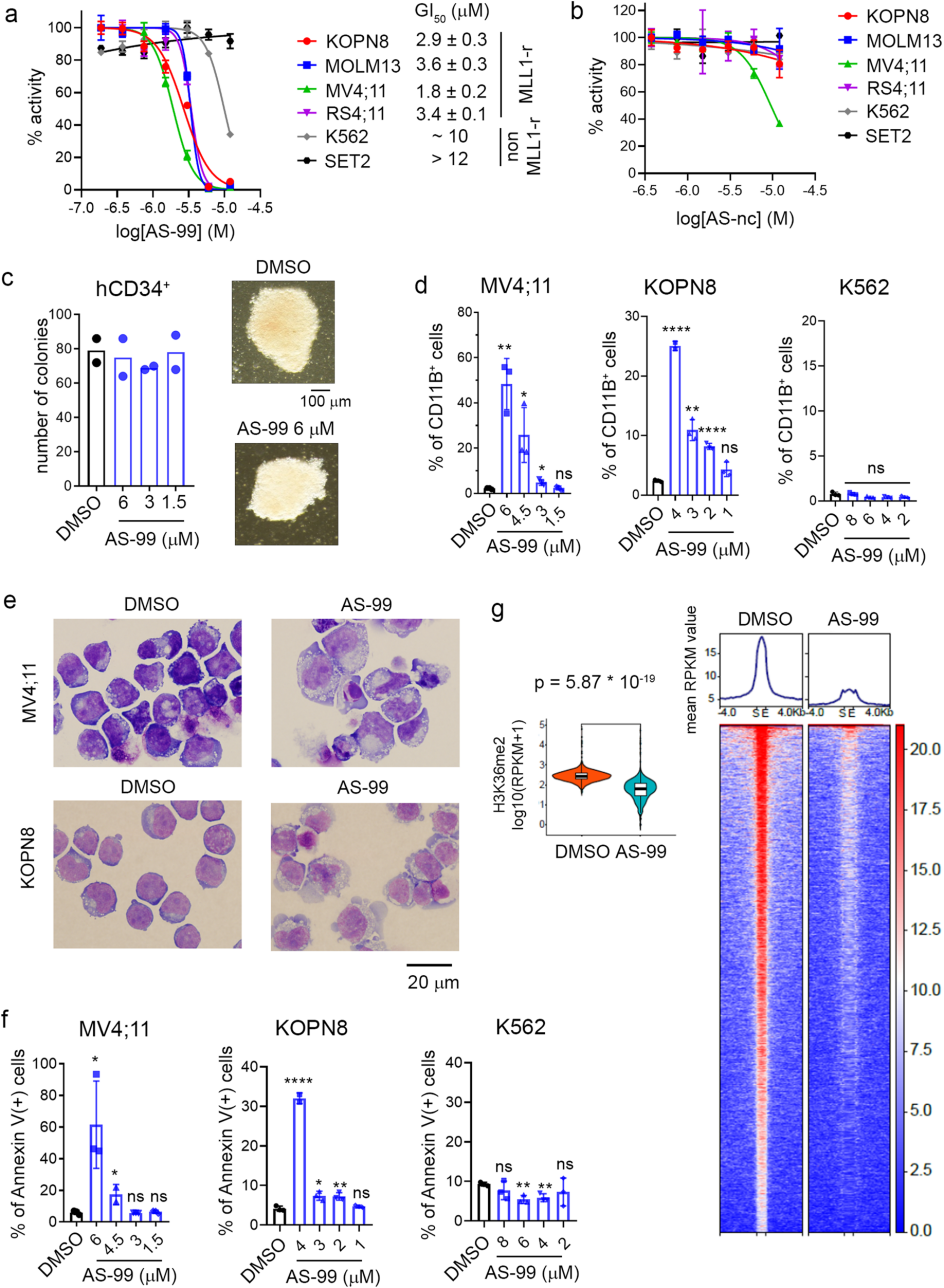
Cellular activity of ASH1L inhibitor AS-99. **a**, **b **Titration curves from the MTT cell viability assay performed after 7 days of treatment of human *MLL1* rearranged (MLL1-r) leukemia cell lines (MV4;11, MOLM13, KOPN8, RS4;11) and control leukemia cell lines, non–MLL1-r (K562 and SET2) with **AS-99** (**a**) or **AS-nc** (**b**); mean ± SD, *n* = 4 biological replicates. Representative graphs are shown from 2–3 independent experiments. GI_50_ values correspond to **AS-99** concentrations required to achieve 50% inhibition of cell proliferation. **c** Colony counts and representative images of colonies from the colony-forming assay performed with normal human hematopoietic CD34^+^ cells isolated from cord blood treated for 7 days with DMSO or **AS-99**; mean ± SD, *n* = 2. The experiment was performed twice. Representative data is shown. **d** Quantification of CD11B expression in human leukemia cells treated for 7 days with **AS-99**, detected by flow cytometry; mean ± SD, *n* = 3 biological replicates. *P* values (MV4;11: 6 µM: 0.0021, 4.5 µM: 0.026, 3 µM: 0.028, 1.5 µM: 0.73); KOPN8 (4 µM <0.0001, 3 µM: 0.0012, 2 µM: <0.0001, 1 µM: 0.055); K562 (8 µM: 0.78, 6 µM: 0.070, 4 µM: 0.055, 2 µM: 0.081) were calculated using unpaired 2-tailed *t* test. Two independent experiments were performed for each cell line in triplicates. Representative graphs are shown. Gating strategy is presented in Supplementary Fig. 8c. **e** Wright-Giemsa–stained cytospins for MV4;11 and KOPN8 cells after 7 days of treatment with DMSO or **AS-99**: in MV4;11 at 6 µM and in KOPN8 at 4 µM. **f** Flow cytometry analysis of apoptosis induced by **AS-99** in MV4;11, KOPN8, and K562 cells after 7 days of treatment. Mean ± SD, *n* = 3 biological replicates. *P* values (MV4;11: 6 µM: 0.026, 4.5 µM: 0.049, 3 µM: 0.65, 1.5 µM: 0.70; KOPN8: 4 µM: < 0.0001, 3 µM: 0.012, 2 µM: 0.010, 1 µM: 0.24; K562: 8 µM: 0.31, 6 µM: 0.004, 4 µM: 0.006, 2 µM: 0.39) were calculated using unpaired 2-tailed *t* test. Two independent experiments were performed in triplicates. Representative graphs are shown. *P < 0.05; **P < 0.01; ****P < 0.0001; *NS* not significant. Gating strategy is presented in Supplementary Fig. 8d. **g** CUT&RUN experiment in MV4;11 cells treated with **AS-99** (5.5 µM) or DMSO showing H3K36me2 peaks. The rows show the RPKM (Reads Per Kilobase Million) values on the peaks (normalized to 1 kb) and 4 kb regions flanking the peaks. Peaks were sorted by total normalized signals of DMSO-treated cells within each category. Violin plots show the RPKM values of each peak in DMSO and **AS-99** treated cells. The boxes represent 25th (minima) and 75th (maxima) percentile and the lines represent the median. The high and low whisker ends are the largest values within 1.5-times interquartile range above 75th and the smallest value within 1.5-times interquartile range below the 25th percentile, respectively. Mann–Whitney two-sided *U* test was used for statistical analysis to compare the difference of signals between DMSO- and **AS-99**-treated cells.

Based on our findings demonstrating the importance of the ASH1L SET domain in MLL leukemia (Fig. 1), we evaluated the effect of the ASH1L inhibitors in a panel of leukemia cells with *MLL1* translocations. First, we found that **AS-85** inhibits the growth of leukemia cells (MV4;11, MOLM13, and KOPN8) harboring different *MLL1* translocations, with GI_50_ values ranging from 5 µM to 25 µM, and demonstrates no effect in K562 leukemia cells without *MLL1* translocations (Supplementary Fig. 7a,b). We also observed time-dependent cell growth inhibition for **AS-85**, a characteristic feature of histone-methyltransferase inhibitors^24,25^, with 7–10 days representing an optimal treatment time (Supplementary Fig. 7a). Furthermore, a more pronounced effect on the growth of the MLL leukemia cells was observed for **AS-99**, with the GI_50_ values ranging from 1.8 µM to 3.6 µM (Fig. 5a), likely due to the improved cell permeability over **AS-85** resulting from the methyl substitution of the basic nitrogen in the azetidine ring (Fig. 4a), which led to higher cLogP and decreased tPSA (Supplementary Table 2). Importantly, **AS-99** showed a several fold weaker effect on the proliferation of leukemia cells without *MLL1* translocations, such as SET2 and K562, with no or limited effects at 10 µM or higher concentrations (Fig. 5a). Finally, we also tested the structurally related **AS-nc** (serving as a negative control) in MLL leukemia cells and found no or much weaker effects (Fig. 5b). To assess potential toxicity in normal cells, we tested **AS-99** in normal hematopoietic progenitor cells (human CD34 + cells from cord blood) and found no effect on colony number or morphology (Fig. 5c).

ASH1L knockdown^9^ and deletion of the ASH1L SET domain (Fig. 1) induced differentiation and apoptosis of MLL leukemia cells. Similarly, pharmacologic inhibition of ASH1L with **AS-99**, but not **AS-nc**, induced differentiation in MLL leukemia cells (MV4;11 and KOPN8) as assessed by markedly (up to 50%) increased expression of the CD11B differentiation marker and cell morphology changes (Fig. 5d, e and Supplementary Fig. 8a). These effects were not observed in the control leukemia cell line K562, thus supporting the on-target mechanism of action of **AS-99** (Fig. 5d, e). In addition, **AS-99** also induced apoptosis in the MLL leukemia cells, but not in the K562 cells, as assessed by the quantification of the Annexin V positive cells (Fig. 5f and Supplementary Fig. 8b). The effects on differentiation or apoptosis were not observed upon treatment of MLL leukemia cells with **AS-nc** (Supplementary Fig. 9a–c), further supporting the on-target activity of **AS-99**.

To assess whether **AS-99** blocks H3K36 methylation in cells, we performed a CUT&RUN experiment in MV4;11 cells. Indeed, treatment with **AS-99** resulted in a reduced number of H3K36me2 peaks when compared to the DMSO-treated cells, confirming that **AS-99** blocks the enzymatic activity of ASH1L in the MLL1-rearranged leukemia cells (Fig. 5g). Overall, these results further support the on-target activity of **AS-99** in leukemia cells.

### AS-99 suppresses MLL fusion driven transcriptional programs

**Fig. 6:**
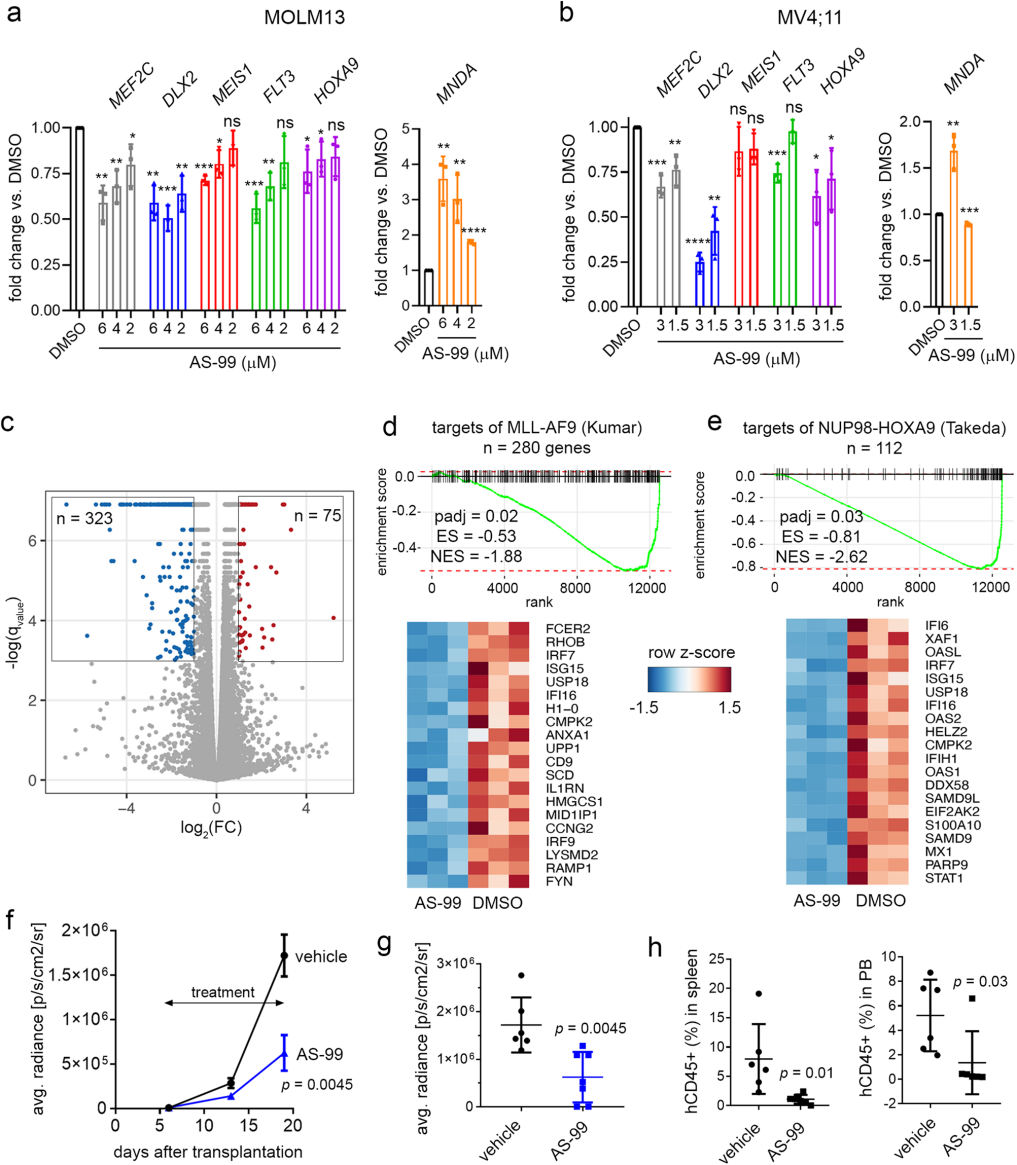
AS-99 impairs transcriptional program of MLL fusion proteins and reduces leukemia burden. **a**, **b** Quantitative RT-PCR performed in MOLM13 cells (**a**) or MV4;11 cells (**b**) after 7 days of treatment with **AS-99**. Gene expression was normalized to *HPRT1* and referenced to the DMSO treated cells. Representative data from two independent experiments, each performed in triplicates (mean ± SD, *n* = 3 biological replicates) are shown. *P* values were calculated using unpaired 2-tailed *t* test. For gene expression in MOLM13 cells, the *P* values are as follows: *MEF2C*: 6 µM: 0.0017, 4 µM: 0.0035, 2 µM: 0.036, *DLX2*: 6 µM 0.0017, 4 µM: 0.0003, 2 µM: 0.0032, *MEIS1*: 6 µM: < 0.0001, 4 µM: 0.011, 2 µM: 0.12; *FLT3*: 6 µM: 0.0007, 4 µM: 0.0018, 2 µM: 0.086; *HOXA9*: 6 µM: 0.026, 4 µM: 0.038, 2 µM: 0.064; *MNDA*: 6 µM: 0.0021, 4 µM: 0.0068, 2 µM: <0.0001. For gene expression in MV4;11 cells, the *P* values are as follows: *MEF2C*: 3 µM: 0.0007, 1.5 µM: 0.009, *DLX2*: 3 µM: <0.0001, 1.5 µM: 0.0017, *MEIS1*: 3 µM: 0.16, 1.5 µM: 0.074, *FLT3*: 3 µM: 0.0010, 1.5 µM: 0.57, *HOXA9*: 3 µM: 0.010, 1.5 µM: 0.044, *MNDA*: 3 µM: 0.0031, 1.5 µM: 0.0004. *P < 0.05; **P < 0.01; ***P < 0.001; ****P < 0.0001; *NS* not significant. **c** Results from RNA-seq studies for **AS-99** (3 µM) versus DMSO-treated MV4;11 cells. Volcano plot showing q-values for differential expression analysis of each gene versus fold-change (FC) for **AS-99** over DMSO-treated cells. Blue dots indicate significantly downregulated genes, red dots indicate significantly upregulated genes (*q* < 0.05, fold-change > 2). **d**, **e** Plots showing enriched gene sets upon treatment of MV4;11 cells with **AS-99** or DMSO determined by fgsea for defined targets of MLL-AF9^27^ (**d**) or targets of NUP98-HOXA9^28^ (**e**). The top 20 differentially expressed genes from the Leading Edge of the indicated fgsea analysis are shown below each of the enrichment plots. padj = adjusted *p* value; *ES* enrichment score, *NES* normalized enrichment score. **f**–**h** Effect of **AS-99** (30 mg/kg, q.d., i.p.) on leukemia burden in the MV4;11 xenotransplantation murine model. Quantification of bioluminescence signal in mice treated with **AS-99** (*n* = 7 mice) or vehicle (*n* = 6 mice) at the indicated days. Mean ± SEM (**f**). Quantification of bioluminescence signal shown for individual mice at the last day of treatment (day 19 post-transplantation). Mean ± SD (**g**). Flow cytometry quantification of human CD45+ cells in spleen, and blood samples harvested from the mice after last day of treatment with **AS-99** (*n* = 7 mice) or vehicle (*n* = 6 mice) (**h**). Mean ± SD. *P* values were calculated using the unpaired 2-tailed *t* test. Gating strategy is presented in Supplementary Fig. 11e.

To evaluate the mechanism of action of the ASH1L inhibitors, we performed gene expression studies with **AS-99** and **AS-nc** in MOLM13 and MV4;11 MLL leukemia cells harboring *MLL-AF9* and *MLL-AF4* translocations, respectively. The treatment of these cells with **AS-99** led to a dose-dependent downregulation of canonical MLL fusion target genes required for leukemogenesis^9,19,26^, including *MEF2C*, *DLX2*, *FLT3*, and *HOXA9* (Fig. 6a, b). In contrast, no changes in the expression of these genes were observed for the negative control compound **AS-nc**, (Supplementary Fig. 10a, b). These effects were accompanied by a substantial increase in the expression level of the *MNDA* differentiation marker upon treatment with **AS-99**, but not with **AS-nc**, (Fig. 6a, b and Supplementary Fig. 10a, b), which is consistent with the differentiating phenotype of these cells (Fig. 5e).

Next, we performed global gene expression studies using RNA-seq after seven days of treatment of MV4;11 cells with **AS-99** and observed more downregulated (323) than up-regulated (75) genes (Fig. 6c). This was found to be consistent with reduced H3K36me2 level (Fig. 5g), which is associated with active transcription^2^. Importantly, Gene Set Enrichment Analysis (GSEA) revealed the downregulation of transcriptional programs driven by MLL fusion proteins and *HOXA9* upon treatment of MV4;11 cells with **AS-99**. For example, **AS-99** led to the downregulation of genes induced by the expression of MLL-AF9 (Fig. 6d)^27^. Furthermore, **AS-99** resulted in the suppression of the genes upregulated by *NUP98-HOXA9* oncogene^28^, suggesting that our ASH1L inhibitor represses the *HOXA9*-regulated program (Fig. 6e). All of these results strongly support that the inhibition of ASH1L catalytic activity with a small molecule inhibitor reverses the leukemogenic activity of MLL fusion proteins.

### AS-99 reduces leukemia burden in mice

To assess the utility of AS-99 for in vivo studies in mice we performed pharmacokinetic (PK) studies of this compound in mice, which revealed favorable exposure in plasma upon i.v. and i.p. administration (AUC = 9701 hr* ng/mL and 10,699 hr* ng/mL, respectively), suitable half-life (~5–6 h) and C_max_ > 10 µM, Supplementary Fig. 11a,b. To evaluate the potential therapeutic effects of the ASH1L inhibitors, we performed an in vivo study with **AS-99** using a xenotransplantation model of MLL leukemia with MV4;11 cells expressing luciferase transplanted into NSG mice^29^. The treatment with **AS-99** (30 mg/kg, i.p., q.d.) or vehicle was initiated six days after transplantation and was continued for 14 consecutive days with the progression of the diseases monitored by bioluminescence signals (Fig. 6f). No substantial toxicity was observed during the treatment period as indicated by measurement of mice body weight, which was only slightly reduced (by ~10%) after 7 days of treatment with **AS-99**, but this difference became less pronounced as the treatment continued (Supplementary Fig. 11c). Importantly, the mice treated with **AS-99** demonstrated a 65% reduction in the bioluminescence signal when compared to the vehicle-treated control mice (Fig. 6f, g). This effect was accompanied by a significantly lower level of leukemic blasts (human CD45 + cells) in the peripheral blood and spleen of the **AS-99**-treated mice when compared to the vehicle-treated mice on the last day of the treatment (Fig. 6h). We have also assessed the effect of **AS-99** in normal mice using the same treatment schedule as in the leukemia model and found no significant difference on blood counts and other blood parameters between **AS-99** and vehicle-treated mice, Supplementary Fig. 12a. The T-cells were unaffected while myeloid and B-cells were slightly changed (increased and reduced, respectively) in the **AS-99**-treated mice, although they remained in the normal range, Supplementary Fig. 12b. Overall, **AS-99** reduced the leukemia burden in the xenotransplantation mouse model of MLL leukemia without affecting blood counts in normal mice, indicating that this compound can serve as a valuable chemical probe and a candidate for further development.

## Discussion

In this study, we validated the role of the catalytic ASH1L SET domain in MLL leukemia. Importantly, mice deficient with Ash1l^30^ or with a loss of the Ash1l SET domain^5^ remained viable with no major developmental defects, suggesting that targeting ASH1L may have limited or no side effects and supporting ASH1L as an attractive target for drug discovery. However, ASH1L is a challenging target for inhibitor development as the SET domain adapts an inactive conformation with the autoinhibitory loop blocking access to the active site^2,7^. By applying fragment-based screening, followed by medicinal chemistry and a structure-based design, we developed first-in-class and potent ASH1L inhibitors, including **AS-99** (IC_50_ = 0.79 µM, K_d_ = 0.89 µM). The systematic application of biochemical, biophysical, and structural studies resulted in ASH1L inhibitors with over 1000-fold improved binding affinity over the initial fragment hit. Thus, our study reveals the tractability of the ASH1L SET domain for inhibition by small molecules, as no such compounds were reported before. One of the striking features of our ASH1L inhibitors essential for high potency is the presence of the thioamide group, as its replacement by an amide results in ~100-fold reduced inhibitory activity. This is likely due to the loss of a chalcogen bond involving sulfur from the thioamide group and electrostatic repulsion between the oxygen in the amide analog and the carbonyl group of H2193 (Supplementary Fig. 5c). This example emphasizes favorable interactions of sulfur in protein-ligand complexes.

Structural studies demonstrate that the inhibitors we developed bind to a site adjacent to the autoinhibitory loop in the SET domain, possibly stabilizing the conformation in this region and allosterically blocking the enzymatic activity of ASH1L. Interestingly, the autoinhibitory loop, which is present in only four other H3K36 histone methyltransferases (NSD1, NSD2, NSD3, and SETD2)^2^, has poorly conserved amino acid sequences, providing a rationale for the >100-fold selectivity of **AS-99** to ASH1L over other HMTs.

When tested in MLL leukemia cells, **AS-99** downregulated the expression of MLL fusion target genes and reduced H3K36 dimethylation, strongly supporting an on-target mechanism of action. The GSEA analysis revealed that **AS-99** suppressed transcriptional programs driven by MLL fusion proteins and *HOXA9*. Treatment with **AS-99** also inhibited proliferation and induced differentiation and apoptosis in MLL leukemia cells, but not in control cell lines without *MLL1* translocation, excluding general toxicity. Thus, **AS-99** recapitulates the effects observed upon genetic inactivation of ASH1L^9^ or deletion of the ASH1L SET domain (this paper). When applied in vivo, **AS-99** reduced the leukemia burden in a systemic model of MLL leukemia, supporting the inhibition of ASH1L as an approach to target leukemia with *MLL1* translocations.

In summary, the **AS-99** compound that we report here represents a first-in-class, well characterized, and selective ASH1L inhibitor with sub-micromolar in vitro activity. This compound can serve as a valuable chemical probe to further explore the biological functions of ASH1L and to address a potential therapeutic benefit of blocking ASH1L in different diseases, including cancer. The outcome of these studies will pave the way towards the development of pharmacological agents targeting the catalytic SET domain of ASH1L.

## Methods

### Protein expression and purification

ASH1L SET domain (amino acids 2069–2288) was expressed and purified as previously described^7^ and was used in ITC, NMR, and X-ray crystallography experiments. Briefly, ASH1L (2069–2288) was fused with an N-terminal Mocr tag^31^ and was expressed in *E. Coli* BL21(DE3) cells. Transformed cells were harvested and lysed, and the supernatant was loaded on a column packed with Ni-NTA agarose for affinity chromatography. The Mocr-ASH1L fusion was eluted in the buffer containing 50 mM Tris, pH = 7.5, 500 mM NaCl, 1 mM TCEP and 500 mM imidazole, followed by the cleavage of Mocr tag using tobacco etch virus (TEV) protease. Cleaved ASH1L was isolated from Mocr by repeating the Ni-NTA affinity chromatography and collecting ASH1L eluted in the buffer containing 50 mM Tris, pH = 7.5, 500 mM NaCl, 1 mM TCEP and 20 mM of imidazole. For X-ray crystallography experiments, the protein was further purified by size-exclusion chromatography using a Superdex-75 column (GE Life Sciences). The ASH1L SET domain with Q2265A mutation (amino acids 2069-2288) was expressed and purified following the same protocol and was used in the X-ray crystallography experiments. An extended construct of the ASH1L SET domain (amino acids 2046-2330) was expressed and purified as reported^21^ and was used in the histone methyltransferase assays.

### NMR-based fragment screening and compound binding to ASH1L

Samples for NMR studies were prepared with 100 µM ^15^N-labeled ASH1L SET domain (2069–2288) in the buffer (50 mM Tris, pH = 7.5, 100 mM NaCl, 1 mM TCEP) mixed with compounds at various concentrations (100–500 µM) with a final DMSO concentration of 5%. For fragment screening, we used an in-house library of 1600 fragment-like compounds with the following properties: average M_w_ = 183 Da (range of 84–345 Da), mean clogP = 1.5 (from −2.5 to 3.7), hydrogen bond acceptors: from 0 to 6 (median = 3), hydrogen bond donors: 0 to 3 (median = 1).

Compounds were screened in mixtures of 20 compounds per sample at 250 μM final concentration of each compound and 5% DMSO. ^1^H-^15^N TROSY-HSQC spectra were acquired at 30 °C on a 600 MHz Bruker Avance III spectrometer equipped with cryoprobe, running Topspin version 2.1. Processing and spectral visualization was performed using NMRPipe v3.0^32^ and Sparky v3.113 (T. D. Goddard and D. G. Kneller, SPARKY 3, University of California, San Francisco).

### Chemical synthesis of ASH1L inhibitors

Description of chemical synthesis of ASH1L inhibitors is provided in the Supplementary Information.

### Histone methyltransferase assay

Histone methyltransferase (HMT) assays were carried out in duplicates with 50 nM ASH1L (amino acids 2046–2330) titrated by varying concentrations of compounds (final DMSO concentration was 2%) and a buffer containing 50 mM Tris pH = 8.5, 2 mM MgCl_2_, 1 mM TCEP and 0.01% Triton X-100. The mixtures of ASH1L and compounds were incubated at room temperature for 1 h. The HMT reactions were initiated by addition of 250 nM chicken nucleosomes (Reaction Biology, HMT-35-179), 1 μM ^3^H-labelled S-adenosyl methionine (Perkin Elmer, NET155V250UC) and 2 μM unlabeled S-adenosyl methionine. The reaction was continued for 1 h at room temperature, then quenched by addition of 10% Trichloroacetic acid (TCA) and then transferred to 96-well filter plates (Millipore sigma, MSFBN6B), washed twice with 10% TCA and then once with 200-proof ethanol. After extensive drying, 70 µL of Microscint-O scintillant (PerkinElmer) was added and counts per minute (CPM) were measured using a MicroBeta microplate counter (PerkinElmer). Titration curves were plotted using Prism (GraphPad Prism 8.0.0.) and IC_50_ values were calculated.

To assess the mechanism of action of ASH1L inhibitors, the IC_50_ values for **AS-6** were determined using varying concentrations of chicken nucleosomes, 0.1 µM ASH1L (2046–2330) and 40 µM of SAM (4 µM ^3^H SAM plus 36 µM SAM). The experiments were performed in duplicates.

The effect of **AS-99** on the inhibition of a panel of histone methyltransferases was assessed by mixing 50 µM of the compound with ASH1L, DOT1L, EZH2, G9a, GLP, NSD1, NSD2, NSD3, MLL1, MLL4, PRDM9, PRMT1, PRMT3, PRMT4, PRMT6, SETD2, SET7/9, SYMD1, SYMD2, or SUV39. These assays were pursued under the conditions provided in Supplementary Table 3.

### Isothermal titration calorimetry

Purified ASH1L (2069–2288) was extensively dialyzed against ITC buffer consisting of 50 mM phosphate (pH = 7.5), 50 mM NaCl, 1 mM TCEP at 4 °C. Compounds were dissolved in DMSO and diluted with the ITC buffer to final concentrations of 150–500 µM in 5% DMSO. ASH1L was diluted with the ITC buffer to the final concentrations of 15–50 µM in 5% DMSO. SAM was introduced to the solutions of ASH1L and compounds to the final concentration of 50 µM to maintain ASH1L stability. All samples were extensively degassed by vacuum aspiration for 20 min prior to measurements. The titrations were performed using a VP-ITC titration calorimetric system (MicroCal) at 25 °C. The titration curves were obtained by injecting 10 µL aliquots of compound solutions into the cell containing ASH1L at a time intervals of 200 s. For **AS-85**, the titration curves were obtained by injecting ASH1L (100–130 µM) to the compound solutions (10–15 µM). All titration data were analyzed with a single-site fitting model using Origin 7.0.

### Crystallization of ASH1L-inhibitor complexes

For crystallization of ASH1L Q2265A in complex with **AS-5**, 7 mg/mL of ASH1L Q2265A (2069–2288) in 50 mM Tris pH = 7.5, 100 mM NaCl, 1 mM TCEP buffer was mixed with **AS-5** (1:1 ratio) to reach final DMSO concentration of 5%. Crystals were obtained using the sitting drop method by adding the crystallization buffer containing 0.1 M Tris (pH = 8.5), 0.2 M MgCl_2_, and 30% PEG4000 to the mixture of ASH1L Q2265A—inhibitor complex (1:1 ratio). Crystallization plates were incubated at 17 °C. For cryoprotection, crystals were soaked in the crystallization buffer solution containing 25% PEG400 and were flash-frozen in the liquid nitrogen, prior to data collection. For crystallization of ASH1L in complex with **AS-85**, ASH1L (2069–2288) was concentrated to 8.4 mg/mL and was incubated with **AS-85** at 1:1 molar ratio in a final DMSO concentration of 2%. Crystals were obtained using the sitting drop method in 0.1 M HEPES (pH 7.5), 30% PEG3350 at 17 °C. For cryoprotection, crystals were soaked in a crystallization buffer containing 25% glycerol and flash-frozen in liquid nitrogen prior to data collection.

### Crystallographic data collection and structure determination

X-ray diffraction data were collected under cryogenic conditions at the 21ID-F, 21ID-G beamlines of the Life Sciences Collaborative Access Team at the Advanced Photon Source. The data were then indexed, integrated, and scaled by HKL2000 v.720^33^. Structures of the complexes were determined by the molecular replacement using MOLREP v.11^34^ and Phaser-MR v.1.17.1^35^ with ASH1L SET domain (PDB: 4YNM) as the search model. Refinement of the structures was performed using Phenix v.1.17.1^36^ and Coot v.0.8.9.2^37^. The structures were validated using MolProbity v.4.02b-467^38^.

### Profiling of AS-99 with kinases

Profiling of **AS-99** was performed at 25 µM compound concentration upon 1 h incubation with a panel of diverse, representative kinases at room temperature. This study was outsourced to the Contract Research Organization.

### Generation of murine bone marrow cells transformed with oncogenes

Generation of C57BL6 mice with the genotype of Ash1l SET domain deletion (ΔSET) was previously described^5^. Preparation of mouse cells transformed with *MLL-AF9*, *MLL-AF6*, *E2A-HLF*, and *HOXA9/MEIS1* (HM-2) oncogenes was performed in a similar way as described previously^39^. Briefly, mice were injected with 5-fluorouracil (Sigma, cat. #F6627) at 150 mg/kg, i.p. Five days later, the Lin-c-kit+ bone marrow cells were isolated from these mice using EasySep kit according to the manufacturer’s protocol (STEMCELL, cat. # 19856). The cells were then transduced via two rounds of spinoculation with retrovirus harvested from PlatE cells transfected with the plasmids encoding: *MLL-AF9*, *MLL-AF6*, *E2A-HLF*, *HOXA9/MEIS1* (HM-2), or MSCV-based vector (plasmids were kindly provided by Dr. Muntean). The transformed cells were then used for four rounds of colony forming assays with serial replating.

### Colony assay with WT and ΔSET Ash1l murine bone marrow cells

Mouse bone marrow cells transformed with various oncogenes or the vector alone were plated in the 35 mm dishes (StemCell, cat # 27100) at the concentration of 1 × 10^4^ cells/mL in the methylcellulose medium (StemCell, cat # M3234) containing 20% IMDM medium, 1% penicillin/streptomycin, murine IL-3 10 ng/ml, IL-6 10 mg/ml, and SCF 50 ng/ml. Plates were incubated at 37 °C, 5% CO_2_ for 7–10 days, at which time colonies were counted. To re-plate cells for the 2nd, 3rd, and 4th rounds, the cells were collected from the plates, washed in PBS twice, counted, and plated in the methylcellulose medium in the same way as in the 1st round. Colonies were counted for each round, and colonies from the 4th round were used for Wright-Giemsa staining.

### Viability assays

Human leukemia cells K562, MV4;11 and RS4;11 (purchased from ATCC) and KOPN8, MOLM13 (purchased from DSMZ) were cultured in RPMI 1640 (Invitrogen) supplemented with 10% heat inactivated (h.i.) FBS and 1% penicillin/streptomycin (Invitrogen). SET2 cells (purchased from DSMZ) were cultured in RPMI 1640 supplemented with 20% h.i. FBS and 1% penicillin/streptomycin. Cells were plated at 1 × 10^5^ to 2 × 10^5^ cells/mL depending on cell line and treated with 0.25% DMSO or varying concentrations of compounds and incubated at 37 °C, 5% CO_2_ incubator for 7 days. Media was changed on day 3 or 4 and compounds were resupplied. Viable cell numbers for DMSO treated samples were restored to the original concentration and the same cell dilution was used for all other samples. Cell suspensions (100 μL) were transferred to the 96-well plates for each sample in quadruplicate at the time of media change. At day 7, 10 µL of MTT reagent (M6494, Molecular Probes™) was added to each well of the 96 well plate and incubated at 37 °C for 4 h. The formazan was then solubilized by adding 100 µL acidified 10% SDS solution and incubated at 37 °C overnight. Plates were read for absorbance at 570 nm using a PHERAstar BMG microplate reader. The experiments were performed two times in quadruplicates with calculation of mean and SD for each condition. Data were analyzed in Prism 8.0.0 to obtain GI_50_ values.

### Real time qRT-PCR

0.1 × 10^6^ cells/mL of MV4;11 or MOLM13 cells were plated in triplicates and treated with DMSO, **AS-99** or **AS-nc**, maintaining the final DMSO concentration at 0.25% and were incubated at 37 °C in a 5% CO_2_ incubator. Cells were counted at day 3 or 4 using Trypan Blue (Thermo Fisher) and media was changed with compounds resupplied at that time point. Viable cell numbers for DMSO treated samples were restored to the original concentration and the same cell dilution was used for other samples. 0.5 × 10^6^ – 3 × 10^6^ cells from each sample were collected at days 7 or 8 of treatment, centrifuged at 211 xg, and RNA was extracted according to the RNeasy Mini Kit protocol (74106, Qiagen). Extracted RNA was quantified using Nanodrop 2000 UV-Vis spectrophotometer and 0.5–1 µg of total RNA was reverse transcribed into cDNA using High-Capacity cDNA Reverse Transcription Kit (4368814, Applied Biosystems™) according to the manufacturer’s protocol. Real-time PCR was performed using the CFX96 Touch Real-Time PCR Detection System (Bio-Rad). TaqMan Gene Expression Master Mix and TaqMan Gene Expression Assays were purchased from Applied Biosystems. Relative quantification of each gene transcript was carried out using the comparative Ct method as described in the Applied Biosystems User Bulletin no. 2. TaqMan Gene Expression Assays were used: *HPRT1*: Hs02800695_m1, *DLX2*: Hs00269993_m1, *FLT3*: Hs00174690_m1, *HOXA9*: Hs00365956_m1, *MEF2C*: Hs00231149_m1, *MEIS1*: Hs00180020_m1, *MNDA*: Hs00935905_m1.

For mouse cells from WT or ΔSET Ash1l background transformed with various oncogenes, cells were collected after the 4th round of colony-forming assay, washed twice with PBS, and counted. 0.5 – 2 × 10^6^ cells were used for RNA extraction. The RNA extraction and Real time PCR procedure were performed in a similar way as described above for human leukemia cell lines. TaqMan Gene Expression Assays were used: *Gapdh*: Hs99999905_m1, *Hoxa9*: Hs00365956_m1, *Meis1*: Hs00180020_m1, *Hoxa5*: Hs00430330_m1, *Hoxa10*: Hs00172012_m1, *Hoxa7*: Hs00600844_m1, *Mef2C*: Hs00231149_m1, *CD11b:* Mm00434455, *Gr-1*: Mm00439154, *Ly-6G*: Mm04934123.

### Flow cytometry analysis

K562, MOLM13, and MV4;11 cells were plated at 1 × 10^5^ cell/mL and treated for 7 or 8 days with **AS-99**, **AS-nc** or DMSO in triplicates in the same way as described for qRT-PCR studies. For Annexin V apoptosis assay, 1 × 10^5^ cells per sample were collected at the end of the treatment, washed with PBS and stained using the FITC Annexin V Apoptosis Detection Kit I (BD Pharmingen™) according to the manufacturer’s instructions. In addition, 1 × 10^5^ cells per sample were stained with Zombie Aqua™ dye (1:100 dilution) for 15 min using the Zombie Aqua™ Fixable Viability Kit (Zombie Aqua™ Fixable Viability Kit, Biolegend^®^) at 1:50 dilution and then for 30 min with an anti-CD11B-PE antibody (982606, ICRF44, Biolegend^®^) or anti-CD11B-Pacific Blue (101224, BD BioLegend) antibody at 1:50 dilution, according to the No-wash Sequential Staining Protocol described by the manufacturer (BioLegend, Zombie Aqua Fixable Viability Kit). Cells were washed twice with 500 µL PBS containing 1% FBS and resuspended in 200 µL PBS with 1% FBS. Flow cytometry experiments were performed on FACSCelesta flow cytometer using BD FACSDiva version 8 and all data were analyzed with FlowJo v.10.6.0 software (Tree Star, Inc.).

### RNA-seq studies

MV4;11 cells were plated at a concentration of 1 × 10^5^ cells/mL and treated with DMSO or 3 µM of **AS-99** for 7 days in a similar way as for qRT-PCR experiments. Total RNA was prepared from cells collected after 7 days of treatment using RNeasy Mini Kit (74106, Qiagen). 2 µg of total RNA for each sample, as quantified using Nanodrop 2000 UV-Vis spectrophotometer, was submitted to the Next-Generation Sequencing Services of the University of Michigan for RNA Sequencing on the Illumina NovaSeq (S4) Sequencer. ERCC RNA Spike-In Mix (4456740, Invitrogen™) was provided to control for variability in RNA expression.

For analysis of RNA-seq data, Fastq generation was performed using Illumina’s bcl2fastq2 software (v2.17). The cutadapt [https://cutadapt.readthedocs.org/en/stable/] and FastQC [http://www.bioinformatics.babraham.ac.uk/projects/fastqc/] tools wrapped in Trim Galore [http://www.bioinformatics.babraham.ac.uk/projects/trim_galore/] were used to trim low quality bases (Q < 20) and adapter for raw sequences. Reads with length <20 bp were removed after trimming. Cleaned RNA-seq reads were mapped to the hg19 genome for human using Tophat2 (2.1.1)^40^, which was shown to be accurate alignment of transcriptomes in the presence of insertions, deletions, and gene fusions. Duplicated reads for pair-end data were removed by SAMtools (v1.5)^41^. Aligned reads were assembled using Cufflinks (v2.2.1) and assembled transcriptome catalog was used as an input for Cuffdiff2^42^ to determine gene expression levels (FPKM, Fragments Per Kilobase per Million mapped reads) and differential expression between conditions using default options. Genes with *p* < 0.05 and fold-change >2 up or down were considered as differentially expressing genes to prepare the Volcano plot, which was generated using the “ggplot2” R package. Gene set analysis was performed and enrichment plots were generated using the “fgsea” package, with the test statistic generated by Cuffdiff2 used to rank genes for gene set enrichment analysis^43^. Fgsea was run using the C2, C5, and C6 curated datasets from MSigDB^44^. Heatmaps for the top 20 ranked genes in the Leading Edge from a given enrichment analysis were generated with the “gplots” package [https://cran.r-project.org/web/packages/gplots/index.html].

### Cytospins and Wrigth-Giemsa staining

0.1 to 1 × 10^5^ cells treated with **AS-99**, **AS-nc** or 0.25% DMSO were collected at day 7 for staining. Cytospins were prepared and stained using the PROTOCOL™ Hema 3™ Manual Staining System (22-122911, Fisher Scientific) as described before^45^. For WT or ΔSET Ash1l murine bone marrow cells transformed with various oncogenes, cells were collected after fourth round of colony assay, washed twice with PBS, and counted. 0.5 to 1 × 10^5^ cells in 100 µL PBS were used for cytospins and staining by applying the same procedure as described before^45^.

### CUT&RUN experiment

MV4;11 cells were plated at 1 × 10^5^ cells/mL and treated with 0.25% DMSO or 5.5 µM **AS-99** in triplicates for 4 days. At day 4, cells were counted using Trypan Blue dye, centrifuged at 211 xg and washed with Ca^++^ and Mg^++^ free 1X DPBS (14190144, Gibco™). 2 × 10^6^ viable cells per sample per antibody were processed for CUT&RUN experiment as previously described^46^. Briefly, cells were collected and washed by PBS at room temperature. 2 × 10^6^ cells were incubated with 30 µL Concanavalin A-coated beads (Bangs Laboratories, cat. # BP531) at room temperature for 10 min followed by overnight incubation at 4 °C with antibody against H3K36me2 (39255, Active Motif, 4 µg/ mL) at 4 °C. Samples were washed with DIG-wash buffer and treated with Protein A-micrococcal nuclease (pA-MN) fusion protein to a final concentration of 1 µg/mL at room temperature for 10 min. Digestion was carried out in the presence of 2 mM CaCl_2_ for 30 min at 0 °C. The digested and released particles were extracted by organic extraction method using phenol, chloroform, and ethanol. Purified DNA was submitted to the Next Generation Sequencing Services at the University of Michigan for sequencing on the Illumina NovaSeq (S4) Sequencer. Spike-in DNA (Micrococcal nuclease-treated Drosophila chromatin) was used for calibration.

For analysis of CUT&RUN data, cleaned sequencing reads were mapped to the hg19 genome for human by using Bowtie2 (v2-2.2.4)^47^ with parameters “-q --phred33 --end-to-end --very-sensitive --no-mixed --no-discordant --phred33 -I 10 -X 700”. Then, duplicated reads were removed using SAMtools (v1.5)^41^. Fragments of sizes >120 bp for H3K36me2 CUT&RUN were selected for downstream analysis. Filtered BAM files of mapping results were merged for the same sample using SAMtools and converted to BED format by using BEDTools v2.28.0^48^. Peak regions were called for each sample by using MACS (v 1.4.2)^49^ from bed files of Cut&Run with parameters “-w -S -p 0.00001 -g hs”. RPKM values for visualization in IGV v2.3^50^ were generated by MACS (V 1.4.2)^49^. Differential peaks between groups were called by MACS2 V2.1.1^51^ subcommand “bdgdiff”. Homer v4.10^52^ was employed to perform gene annotation of peaks and differential peaks. Heatmap of signals of H3K36me2 was plotted by deepTools v2.5.7^53^. Violin plots of H3K36me2 were performed by R package ggpubr v0.4.0.

### PK studies in mice

The pharmacokinetics of AS-99 ASH1L inhibitor was determined in female CD-1 mice (Charles River) following intravenous (i.v.) at 15 mg/kg and Intraperitoneal (i.p.) at 30 mg/kg dosing. **AS-99** was dissolved in the vehicle containing 25% DMSO, 45% PEG 400 and PBS. Serial blood samples (70 µL) were collected over 24 h, centrifuged at 21130 xg for 10 min to get plasma for analysis. Plasma concentrations of **AS-99** were determined by the LC-MS/MS method developed and validated for this study. The LC-MS/MS method consisted of a Shimadzu UFLC 20 A system and chromatographic separation of test compound was achieved using Waters XBridge C18 column (5 cm × 2.1 mm, 3.5 µm). An AB Sciex QTrap 5500 mass spectrometer equipped with an electrospray ionization source (ABI-Sciex, Toronto, Canada) in the positive-ion multiple reaction monitoring (MRM) mode was used for detection. All pharmacokinetic parameters were calculated by noncompartmental methods using WinNonlin^®^ version 3.2 (Pharsight Corporation, Mountain View, CA, USA). Parameters are presented as a mean ± standard deviation (SD).

### In vivo studies using bioimaging model

The in vivo bioimaging model was developed as described previously^29,54^. Briefly, the 8- to 10-week old female NSG mice (Jackson Laboratory) were intravenously injected with 5 × 10^6^ luciferase-expressing MV4;11 cells (received from Dr. Thomas Look, Dana Farber Cancer Institute). On day 6 after transplantation, mice were split into vehicle (*n* = 6) and **AS-99** (*n* = 7) treatment groups and treated for 14 consecutive days with vehicle (25% DMSO and 25% PEG400 in PBS) or **AS-99** (30 mg/kg, q.d., i.p.). For imaging, mice were kept anesthetized with isoflurane, and 50 mg/kg luciferin (E1603, Promega) was administered by intraperitoneal injection. Photonic emission was imaged using the in Vivo Imaging System BLI (IVIS, 200) with total imaging time of 10 s for each mouse.

### Flow cytometry analysis of hCD45 + cells

Cells isolated from spleen or peripheral blood (PB) of mice treated with **AS-99** (30 mg/kg, q.d., i.p.) or vehicle were used for the analysis of the level of human CD45 + (hCD45 + ) cells. Red blood cells were lysed with ACK lysis buffer (10-548E, Lonza). Up to 100,000 cells were washed in PBS (with 1% FBS) and stained with the anti–human CD45-BV421 antibody (563879, HI30, BD Biosciences) at 1:50 dilution for 30 min. Flow cytometry experiments were performed on LSR II, FACSCanto, or FACSAria (BD Biosciences) instruments and data were analyzed with FlowJo v.10.6.0 software (Tree Star, Inc.).

### Effect of AS-99 on normal hematopoiesis

For analysis of the effect of AS-99 on normal hematopoiesis in vivo, 8–10 weeks old female C57BL/6 mice (The Jackson Laboratory) were treated with vehicle (*n* = 9) or **AS-99** (*n* = 9) (30 mg/kg, i.p., q.d.) for 14 days. At the end point of the experiment blood samples were collected, processed and analyzed for complete blood counts (CBC) and to assess populations of myeloid (CD11b + /Gr-1 + ), B- (B220 + /CD19 + ), and T- (CD3 + ) cells using flow cytometry. The following antibodies from BioLegend were used for staining: anti-CD11b (#101208, M1/70), Gr-1 (#108433, RB6-8C5), B220 (#103224, RA3-6B2), CD19 (#115520, 6D5), CD3 (#109211, H57-597), Zombie Aqua-dead cells (#423101) at 1:50 dilution. The analysis of CBC and other blood parameters was performed by the ULAM Animal Diagnostic Laboratory using Element HT5 (HESKA).

### Ethics

Animal experiments performed in this study were approved by the University of Michigan Committee on Use and Care of Animals (UCUCA) and Unit for Laboratory Animal Medicine (ULAM). All mouse studies included randomization and were not blinded.

### Reporting summary

Further information on research design is available in the Nature Research Reporting Summary linked to this article.

## Supplementary information

Supplementary Information

Reporting Summary

## Data Availability

The data that support this study are available from the corresponding authors upon reasonable request. The coordinates for ASH1L-**AS-5** and ASH1L-**AS-85** complexes were deposited in PDB under PDB codes 6X0P [10.2210/pdb6x0p/pdb] and 6WZW [10.2210/pdb6wzw/pdb], respectively. RNA-seq and CUT&RUN data were deposited to GEO under the accession number GSE150087 (SubSeries numbers: GSE150085 and GSE150086 for the RNA-seq and CUT&RUN data, respectively). Source data are provided with this paper.

## Source data

Source Data
