# Supplementary material for: Discovery of first-in-class inhibitors of ASH1L histone methyltransferase with anti-leukemic activity
Source: Nat Commun. 2021 May 14;12:2792. doi: 10.1038/s41467-021-23152-6 (PMC8121805; doi:10.1038/s41467-021-23152-6)
Supplement: Supplementary file 1 — Supplementary Information [file 41467_2021_23152_MOESM1_ESM.pdf]

Discovery of first-in-class inhibitors of ASH1L histone methyltransferase with anti-leukemic activity

David S. Rogawski[1,#], Jing Deng[1,#], Hao Li[1,#], Hongzhi Miao[1], Dmitry Borkin[1], Trupta Purohit[1], Jiho Song[1], Jennifer Chase[2], Shuangjiang Li[1], Juliano Ndoj[1], Szymon Klossowski[1], EunGi Kim[1], Fengbiao Mao[1], Bo Zhou[1], James Ropa[1,3], Marta Z. Krotoska[1], Zhuang Jin[1], Patricia Ernst[4], Xiaomin Feng[5], Gang Huang[5], Kenichi Nishioka[6], Samantha Kelly[7], Miao He[8], Bo Wen[8], Duxin Sun[8], Andrew Muntean[1], Yali Dou[1], Ivan Maillard[2,7], Tomasz Cierpicki[1*] and Jolanta Grembecka[1*]

[1]Department of Pathology, [2]Life Sciences Institute, [8]College of Pharmacy, University of Michigan, Ann Arbor, MI 48109, USA
[3]Department of Microbiology and Immunology, Indiana University School of Medicine, Indianapolis, IN 46202
[4]Department of Pediatrics, University of Colorado Denver, Anschutz Medical Campus, Aurora, CO, 80045
[5]Department of Pathology and Laboratory Medicine, Cincinnati Children's Hospital, OH, 45227
[6]Department of Internal Medicine Musashimurayama Hospital, Enoki 1-1-5, Musashimurayama City, Tokyo, 208-0022, Japan
[7]Division of Hematology-Oncology, Perelman School of Medicine, University of Pennsylvania, Philadelphia, PA, 19104
[#]These authors contributed equally to this work

***Correspondence to:**
Jolanta Grembecka, PhD
Associate Professor
Department of Pathology
University of Michigan
4510C MSRB I
1150 W. Medical Center Drive
Ann Arbor, MI 48109-5605
jolantag@med.umich.edu
Phone: +1-734-615-9319; Fax: +1-734-615-0688

Tomasz Cierpicki, PhD
Associate Professor
Department of Pathology
University of Michigan
4510D MSRB I
1150 W. Medical Center Drive
Ann Arbor, MI 48109-5605
tomaszc@umich.edu
Phone: +1-734-615-9324; Fax: +1-734-615-0688

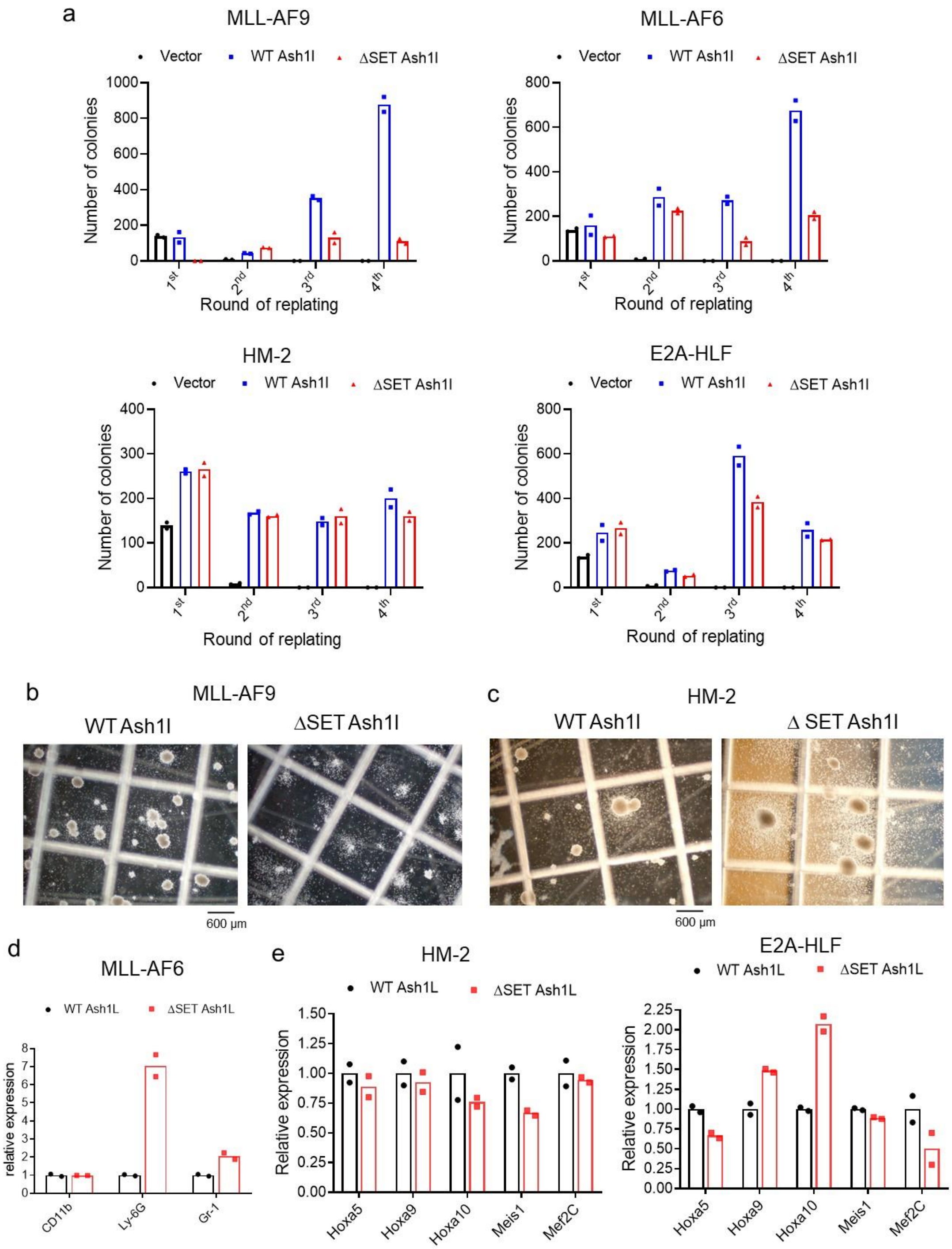

Supplementary Figure 1. SET domain of ASH1L is important for transformation by MLL fusion oncogenes. (**a**) Colony counts from four rounds of colony assays performed in murine bone marrow progenitor cells isolated from WT Ash1l or ΔSET Ash1l mice and transformed with *MLL-AF9*, *MLL-AF6*, *HOXA9/MEIS1* (HM-2), *E2A-HLF* or vector alone (MSCV). mean ± SD, n = 2. (**b**, **c**) Pictures of colonies from the bone marrow cells isolated from WT Ash1l or ΔSET Ash1l mice transduced with *MLL-AF9* (**b**) or *HOXA9/MEIS1* (HM-2) (**c**). **d**) Quantitative RT-PCR performed in bone marrow (BM) cells derived from WT or ΔSET Ash1l mice and transduced with

MLL-AF6 to detect differentiation markers**.** **e**) Quantitative RT-PCR performed in BM cells derived from WT or ΔSET Ash1l mice and transduced with *HOXA9/MEIS1* (*HM-2*) and *E2A-HLF* oncogenes. Gene expression in **d** and **e** was normalized to *Gapdh* and gene expression changes in ΔSET Ash1l cells were referenced to the corresponding values in the WT Ash1l background. Representative data from two independent experiments, each performed in duplicates (n = 2 biological replicates) are shown.

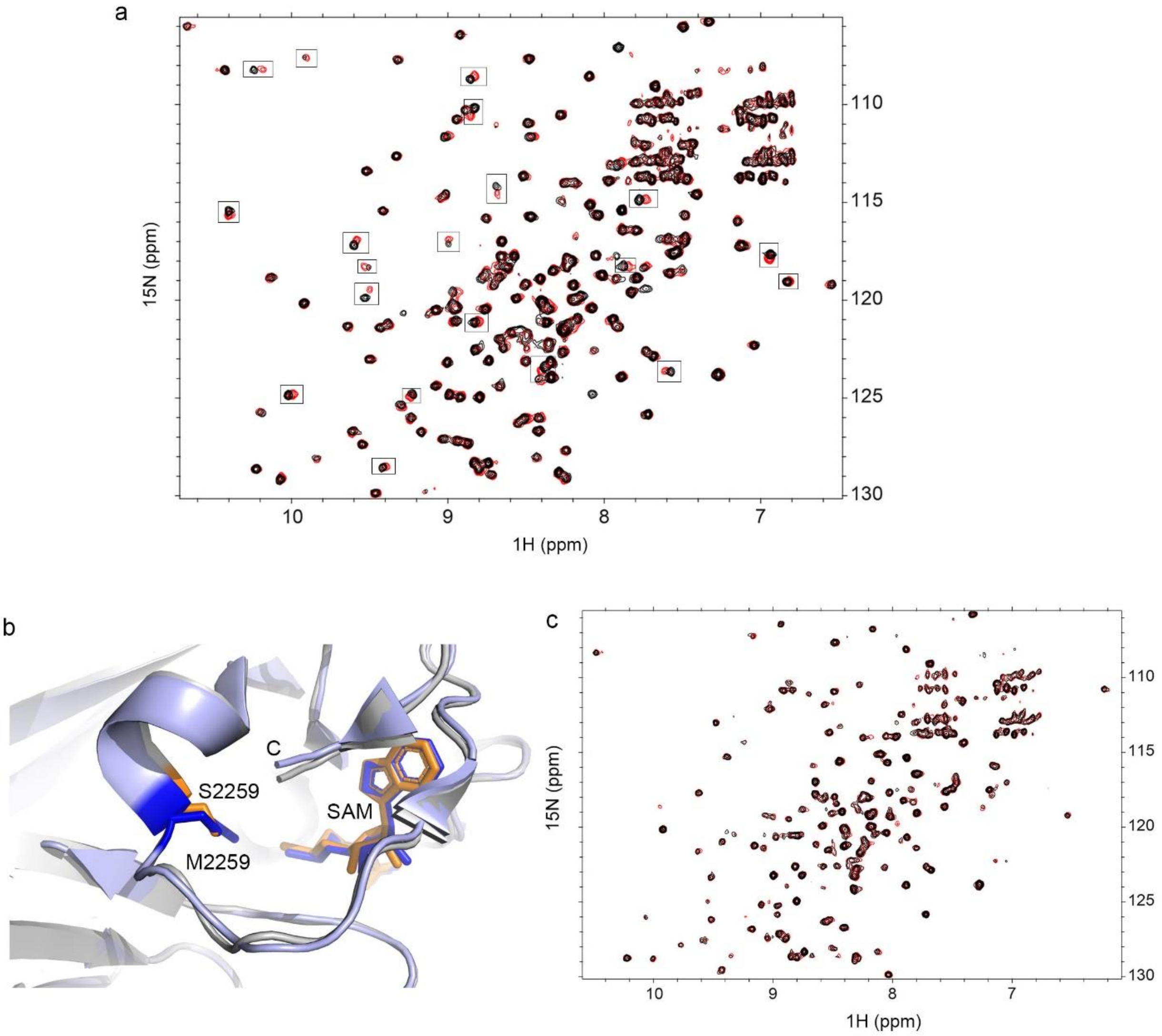

Supplementary Figure 2. Binding of ASH1L inhibitors. (**a**) Superposition of the ^{1}H-^{15}N TROSY-HSQC spectra of 100 µM ASH1L SET with 5% DMSO (black) or with 1 mM of compound **1** (red). Most perturbed residues are marked in boxes. (**b**) Superposition of the crystal structure of ASH1L SET domain (4YNM in PDB) and S2259M mutant of ASH1L (4YNP in PDB). (**c**) Superposition of the ^{1}H-^{15}N TROSY-HSQC spectra of 100 µM ASH1L S2259M mutant with 5% DMSO (black) or with 2 mM of compound **1** (red) demonstrating that compound **1** does not bind to the S2259M mutant.

a

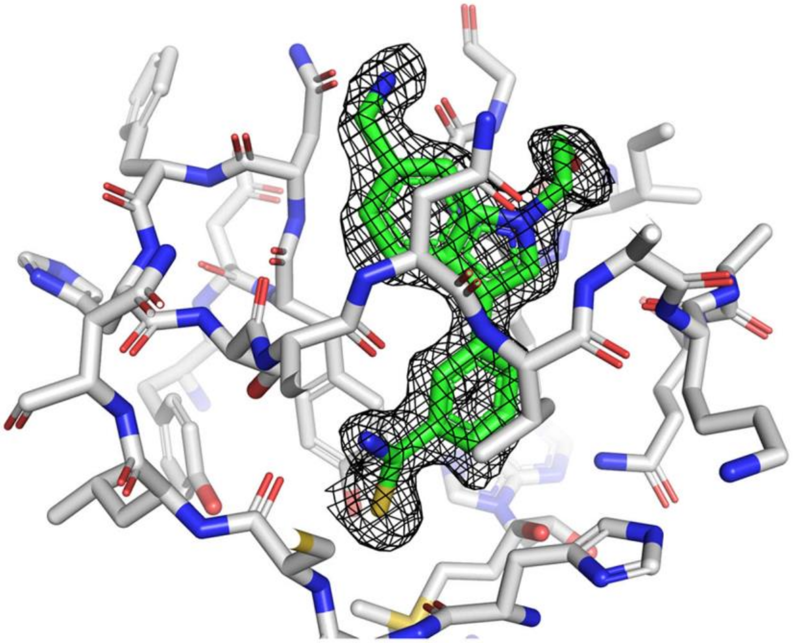

b

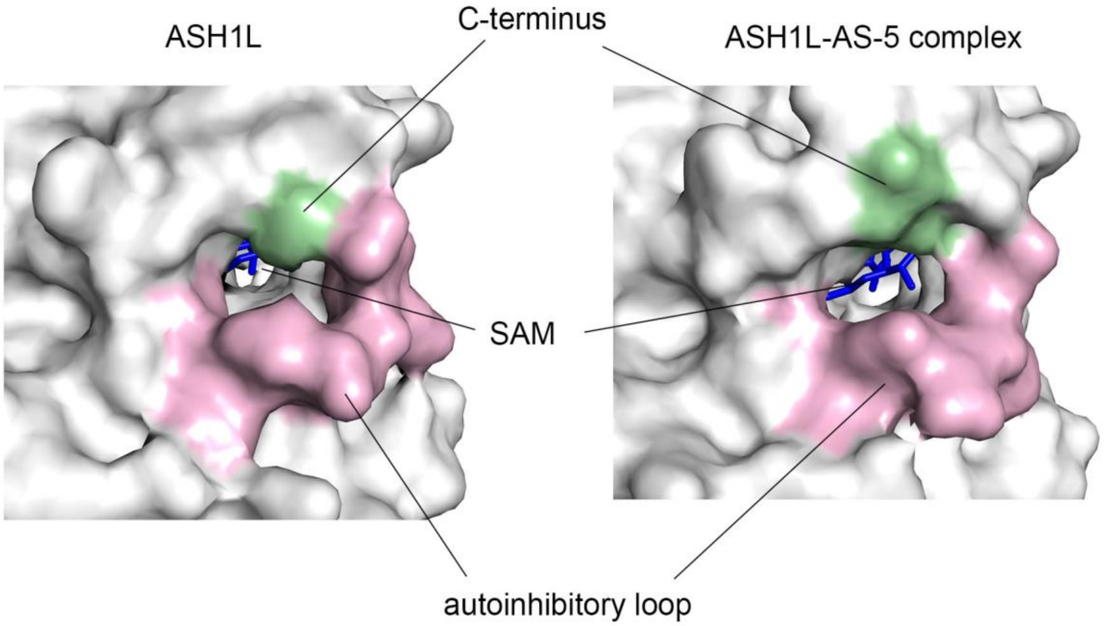

Supplementary Figure 3. Binding of AS-5 to ASH1L. (**a**) The crystal structure of the ASH1L-**AS-5** complex with 2Fo-Fc electron density map contoured at 1σ level for **AS-5**. **(b)** Comparison of the ligand binding site in the structure of ASH1L obtained in the absence of ligang (left) (4YNM in PDB) and in the ASH1L structure derived from the complex with **AS-5** (right). Protein is shown in surface representation. Pink color indicates residues of the autoinhibitory loop, while C-terminal residues of the SET domain are shown in green. SAM cofactor is presented in stick representation in blue.

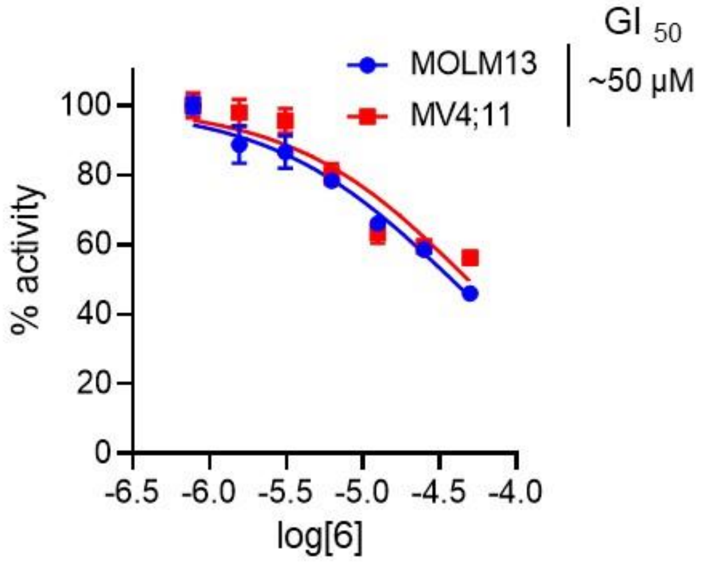

Supplementary Figure 4. Cell viability assay for AS-6 in MLL leukemia cell lines. Titration curves from the MTT cell viability assay performed after 7 days of treatment of human *MLL1*-rearranged leukemia cell lines MV4;11 and MOLM13 with **AS-6** (**6**); mean ± SD, n = 4 biological replicates. Representative graphs are shown from two independent MTT experiments performed for each cell line. GI_{50} values reflect **AS-6** concentration corresponding to 50% inhibition of cell proliferation.

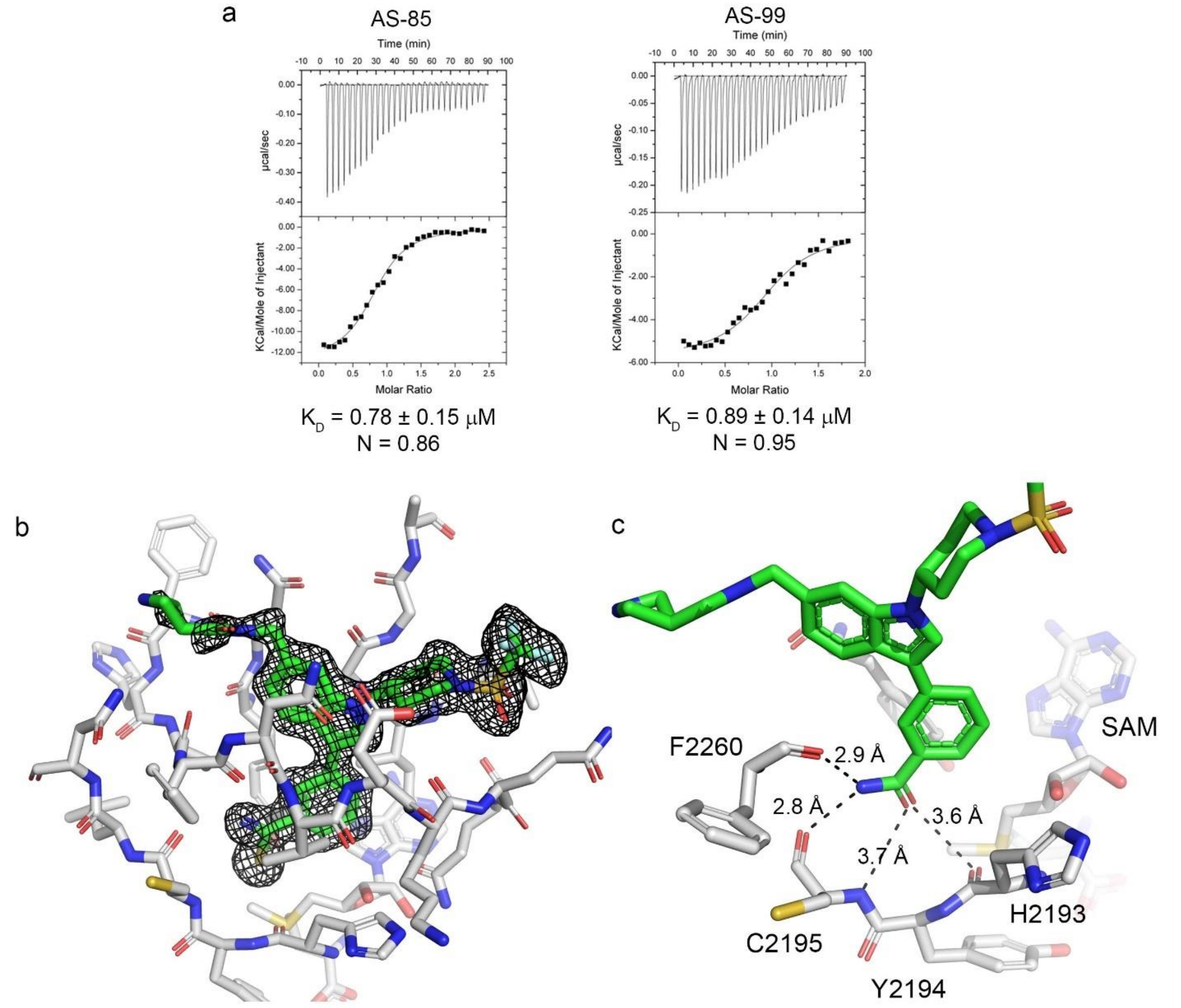

Supplementary Figure 5. Binding of optimized ASH1L inhibitors. (**a**) Binding isotherms from the ITC experiments performed for the binding of **AS-85** and **AS-99** to ASH1L. Data are mean ± s.d. from two independent experiments. Representative binding isotherms are shown. (**b**) Crystal structure of ASH1L SET domain in complex with **AS-85** showing 2Fo-Fc electron density map for **AS-85** contoured at the 1σ level. (**c**) Model of **AS-nc** bound to ASH1L demonstrating predicted interactions of the amide group. Distances between amide nitrogen and oxygen are shown in Å.

kinase	point 1	point 2	ave. % inhibition
ABL1	5	3	4
AKT2	45	41	43
ALK4	5	13	9
AMP-A1B1G1	22	28	25
CDK2-cycA	13	10	11
CDK9-CYCLINT1	3	1	2
CHEK2	33	59	46
CK1-DELTA	-22	-18	-20
CK2A2	-32	-31	-31
CRAF	-23	3	-10
CSK	7	1	4
EPH-B2	9	-2	4
FGFR2	-5	-7	-6
GSK-3-BETA	-6	-8	-7
IGF1R	-9	-12	-10
LCK	-52	-65	-58
MAP2K4	13	14	14
MAPK1	5	-3	1
MAPKAPK-2	9	13	11
MLK2	33	39	36
mTORC1	22	31	26
NEK2	19	14	16
PAK4	-11	-13	-12
PDK1	28	32	30
PRKACA	4	0	2
ROCK2	19	7	13
SRPK1	60	60	60
STK16	12	11	11
STK33	19	18	18
TBK1	18	8	13

Supplementary Figure 6. AS-99 does not inhibit strongly a panel of representative diverse set of kinases. AS-99 was tested at 25 µM in a panel of diverse set of 30 kinases representing the kinome[1]. % inhibition for duplicate samples of **AS-99** (point 1 and point 2) together with average % inhibition are shown.

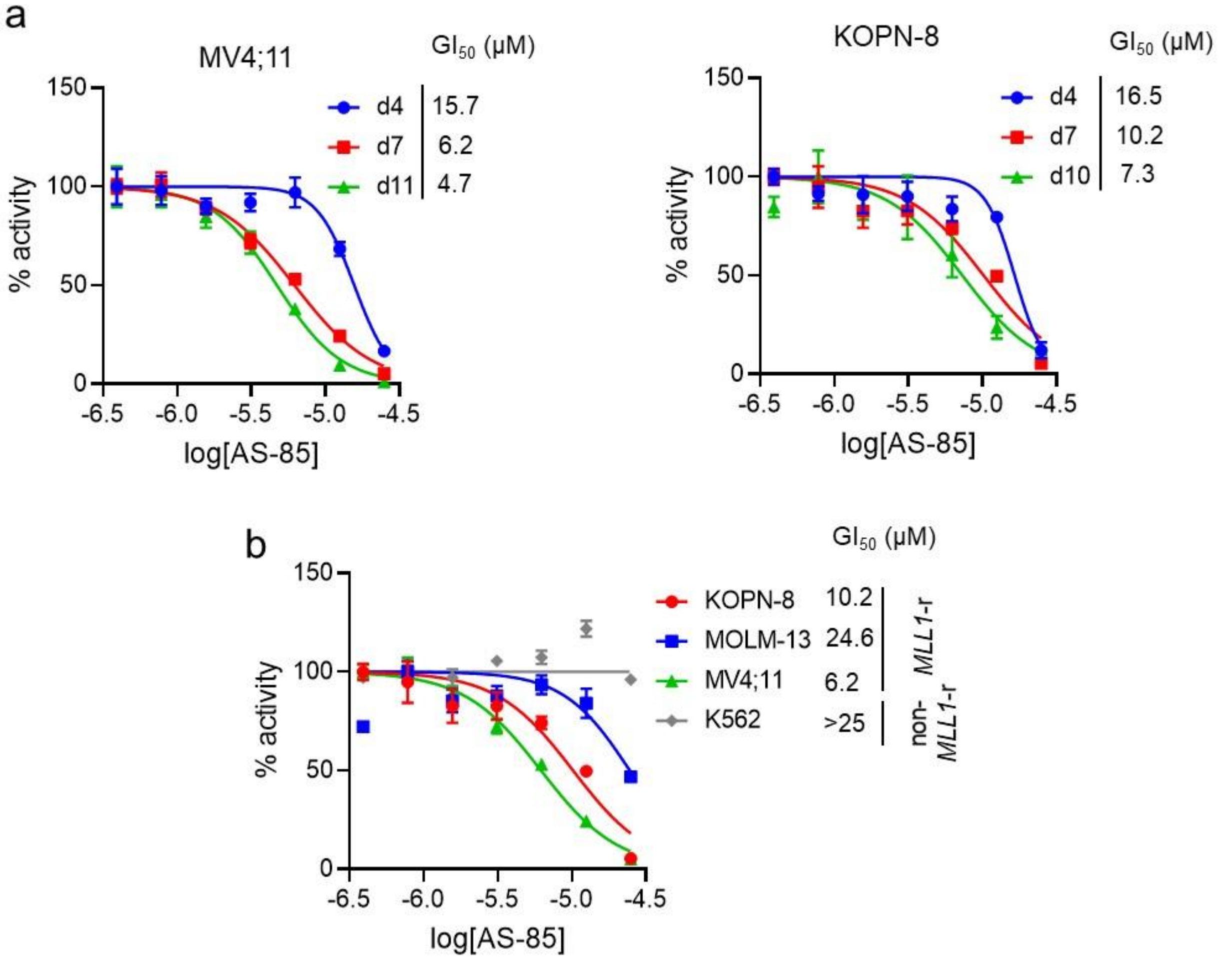

Supplementary Figure 7. Inhibition of cell proliferation in leukemia cells by AS-85. (a) Titration curves from the MTT cell viability assay performed after 4, 7, and 11 (or 10) days of treatment of MV4;11 or KOPN8 cell lines with **AS-85**; mean ± SD, n = 4 biological replicates. (**b**) Titration curves from the MTT cell viability assay performed after 7 days of treatment of KOPN8, MOLM13 and MV4;11 cell lines harboring *MLL1* translocation (*MLL1*-r) or K562 cell line (non-*MLL1*-r) with **AS-85**; mean ± SD, n = 4 biological replicates. Representative graphs are shown from two independent MTT experiments performed for each cell line. GI_{50} values correspond to **AS-85** concentration needed for 50% inhibition of cell proliferation.

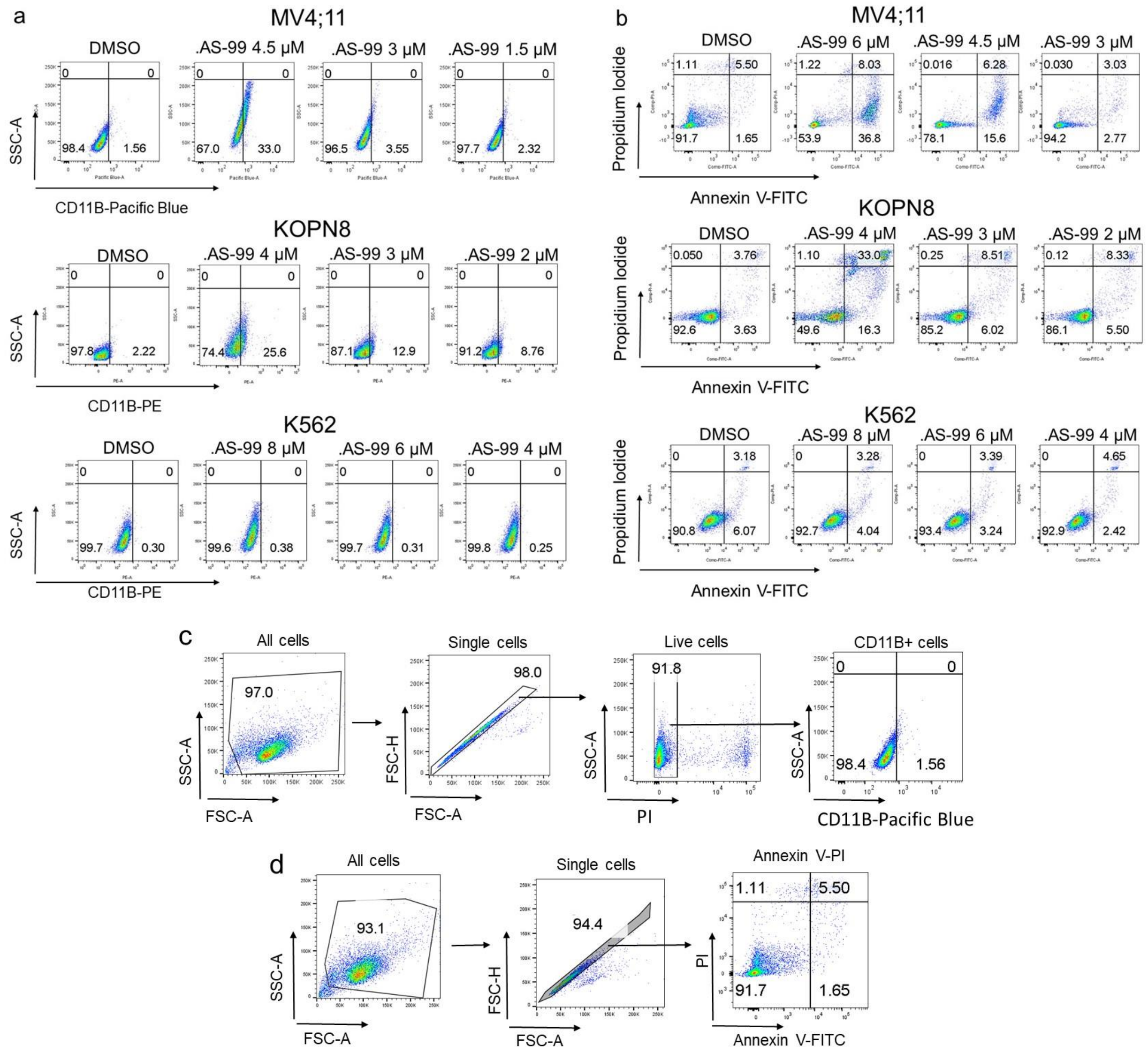

Supplementary Figure 8. AS-99 induces differentiation and apoptosis in MLL leukemia cells. (**a**) Representative histograms from flow cytometry experiments performed in leukemia cell lines upon treatment with **AS-99** using anti-CD11B antibodies to detect differentiation. Pacific blue or PE (Phycoerythrin) were used as fluorophores. SSC-A – side scatter area. (**b**) Representative histograms from flow cytometry experiments performed in leukemia cell lines upon treatment with **AS-99** using Annexin V (labeled with fluorescein, FITC) and Propidium iodide to detect apoptosis. (**c**) Gating strategy corresponding to **Figure 5d** and **Supplementary Figure 8a** for quantification of CD11B-positive cells. Representative gating strategy for MV4;11 cells treated with DMSO is shown. (**d**) Gating strategy corresponding to **Figure 5f** and **Supplementary Figure 8b** for quantification of Annexin V and PI (Propidium iodide) positive cells. Representative gating strategy for MV4;11 cells treated with DMSO is shown. FSC-A – forward scatter area; FSC-H – forward scatter height.

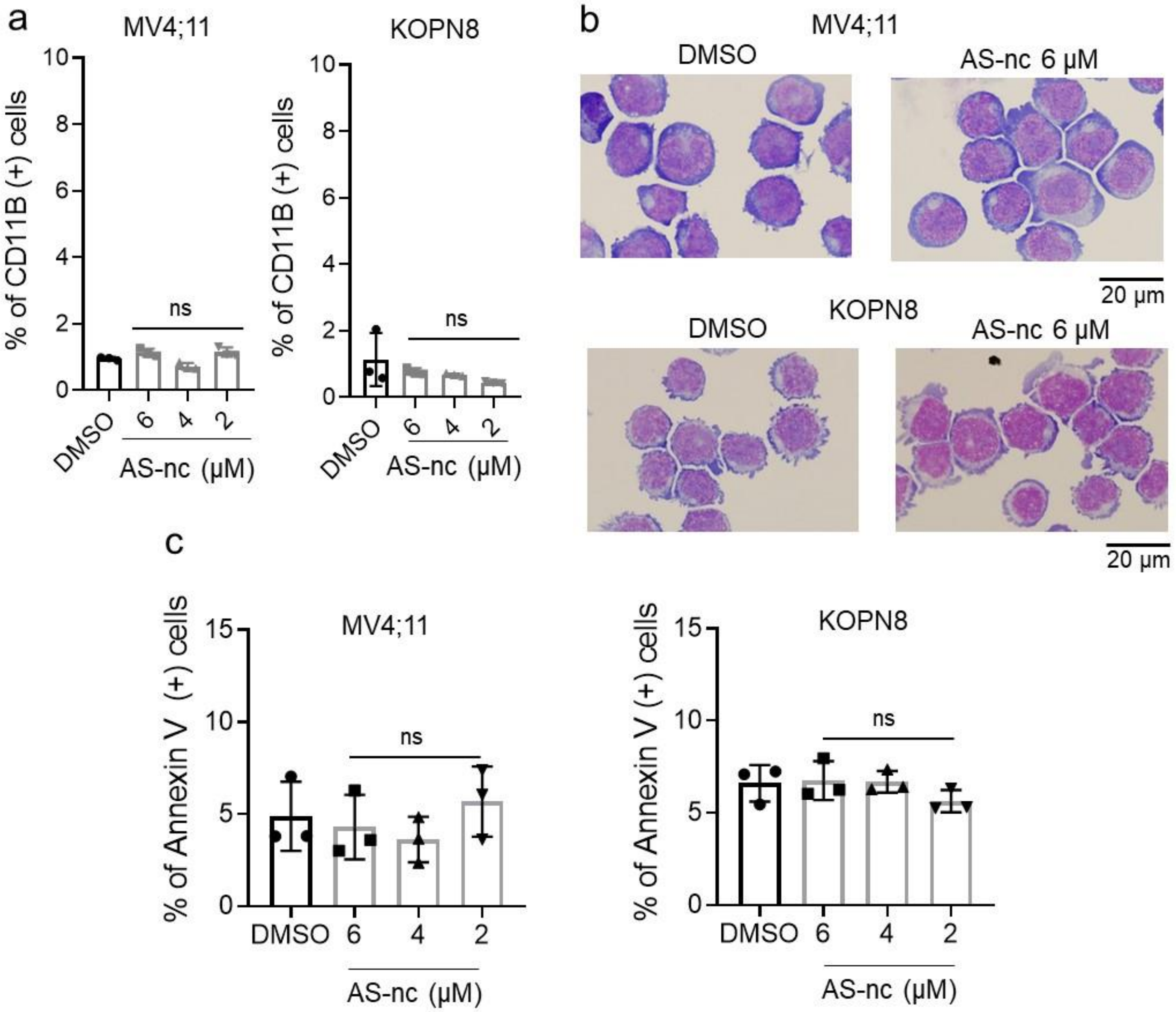

Supplementary Figure 9. Effect of AS-nc in MLL leukemia cells. (a) Quantification of CD11B expression in MV4;11 and KOPN8 human leukemia cells treated for 7 days with **AS-nc,** as detected by flow cytometry; mean ± SD, n = 3 biological replicates. Two independent experiments were performed for each cell line. (**b)** Wright-Giemsa–stained cytospins for MV4;11 and KOPN8 cells after 7 days of treatment with DMSO or 6 µM **AS-nc**. (**c)** Flow cytometry analysis of apoptosis induced by **AS-nc** in MV4;11 and KOPN8 cells after 7 days of treatment. Two independent experiments were performed in triplicates. ns - not significant, calculated using unpaired 2-tailed t test, referenced to DMSO.

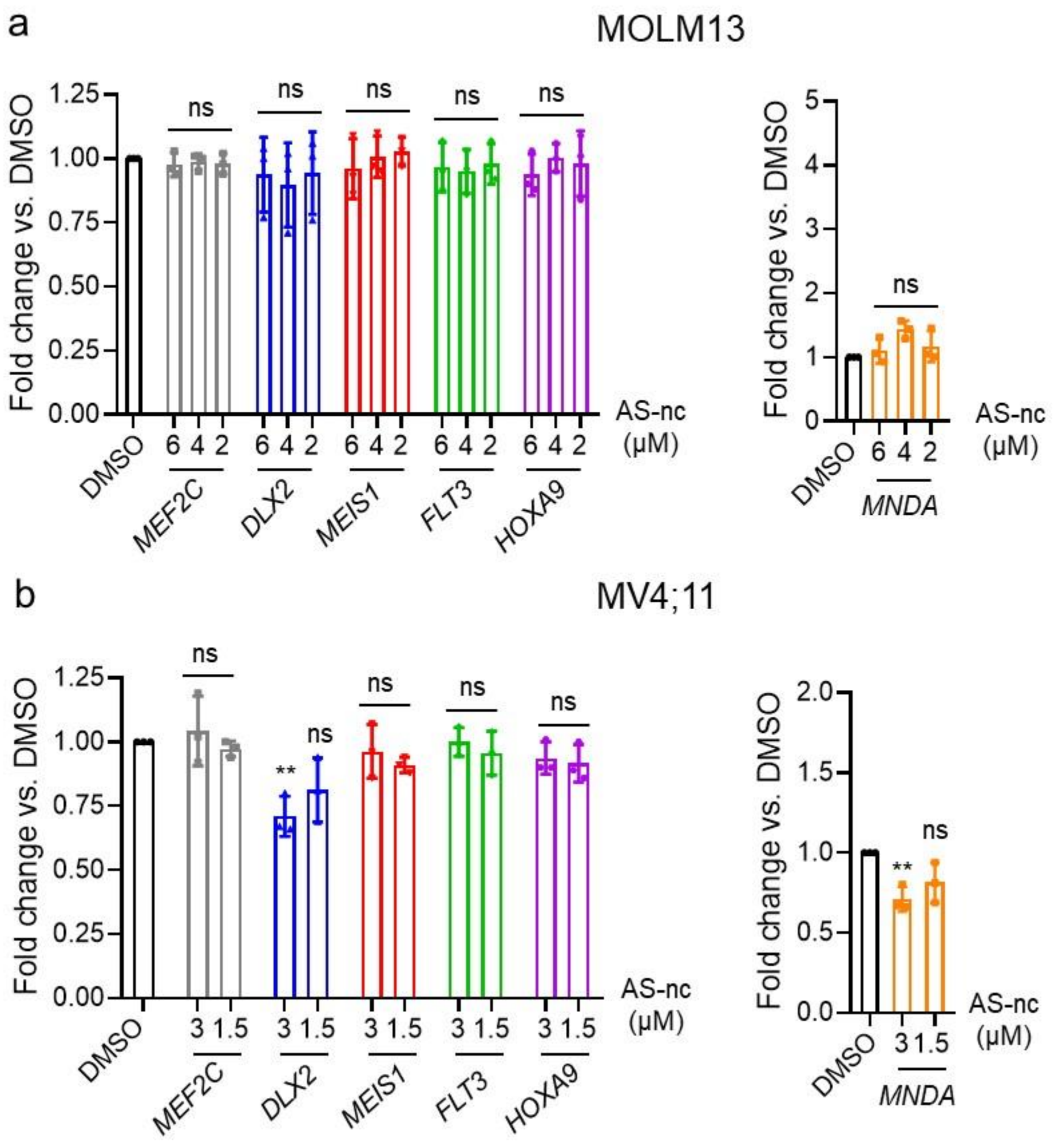

Supplementary Figure 10. Effect of AS-nc on gene expression in MLL leukemia cells. (a, b) Quantitative RT-PCR performed in MOLM13 cells (**a**) or MV4;11 cells (**b**) after 7 days of treatment with **AS-nc**. Gene expression was normalized to *HPRT1* and referenced to the DMSO treated cells. Representative data from two independent experiments, each performed in triplicates, are shown. mean ± SD, n = 3 biological replicates. P values (*DLX2* 3 µM: p=0.003 and *MNDA* 3 µM p= 0.003) were calculated using unpaired 2-tailed t test, referenced to DMSO. **P< 0.01; ns - not significant.

a

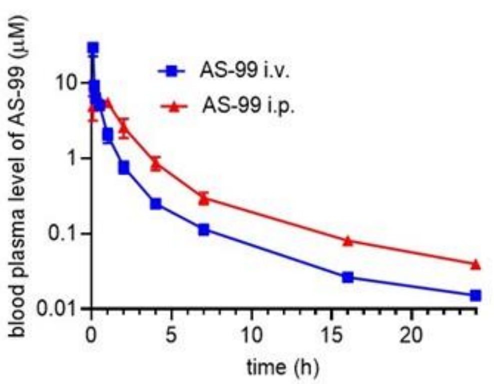

b

Route	Dose (mg/kg)	C_{max} (ng/mL)	T_{max} (hr)	AUC(0-inf) (hr*ng/mL)	$t_{1/2}$ (hr)	CL (mL/hr/kg)
i.v.	15	21400	0.083	9701.6	5.19	1546.5
i.p.	30	5160	0.167	10699.5	5.79	2803.9

c

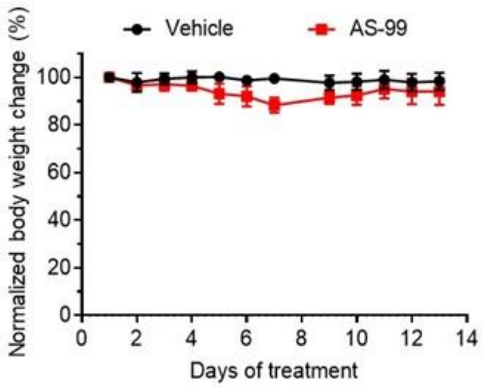

d

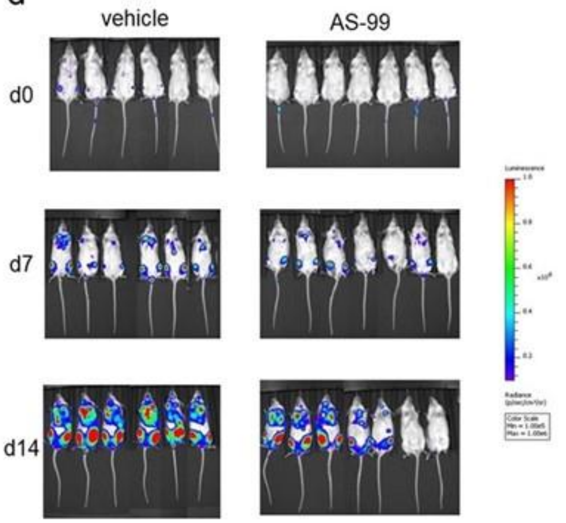

e

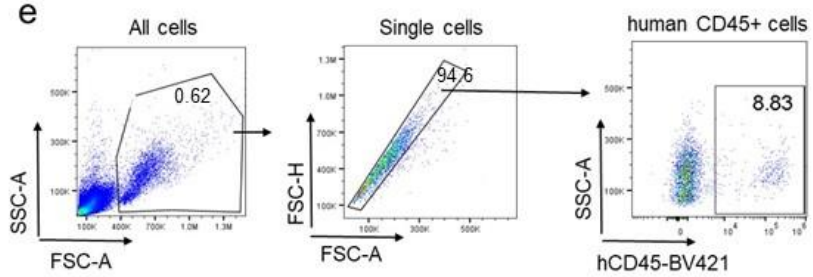

Supplementary Figure 11. PK and *in vivo* efficacy of AS-99. a, b) PK data in mice upon i.v. (15 mg/kg) or i.p. (30 mg/kg) administration of **AS-99**. CL (clearance), AUC (area under the curve), C_{max} (maximum concentration achieved), T_{max} (time at which maximum concentration was achieved), were calculated by noncompartmental methods using WinNonlin® software version 3.2. Mean +/- SEM. n = 3 mice / group. **c**) Body weight of mice monitored upon treatment with **AS-99** (30 mg/kg, q.d., i.p. n=7 mice) or vehicle (n=6 mice) conducted for 14 consecutive days. Measurements are reference to the values obtained at the first day of treatment, which were set as 100%. Mean +/- SEM. **d**) Bioluminescent imaging of NSG mice transplanted with MV4;11 human MLL leukemia cells expressing luciferase performed at the indicated days after initiation of treatment with **AS-99** or vehicle, n = 6-7 mice / group. (**e**) Representative gating strategy corresponding to **Figure 6h** for quantification of human CD45+ cells by flow cytometry in spleen samples.

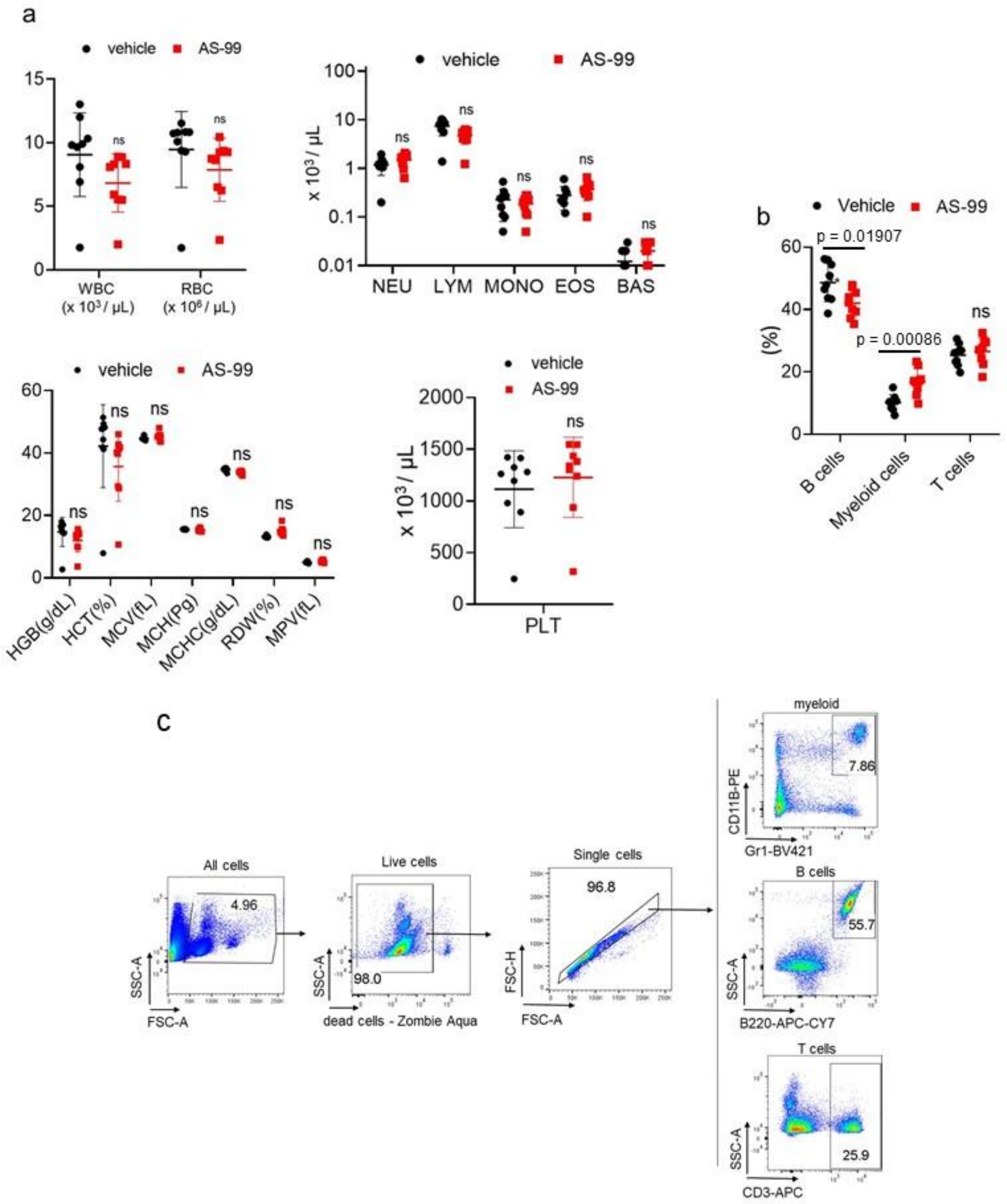

Supplementary Figure 12. Effect of AS-99 in normal mice. (a) Analysis of blood cell counts in normal mice upon treatment with **AS-99** (30 mg/kg, q.d., i.p.) or vehicle for 14 days (WBC – white blood cells, RBC – red blood cells, NEU – neutrophiles, LYM – lymphocytes. MONO – monocytes, EOS – eosinophils, BAS - basophils, PLT - platelets) and other blood parameters (Hgb – Hemoglobin, HCT- Hematocrit, MCV – Mean corpuscular volume, MCH - Mean corpuscular hemoglobin, MCHC - Mean corpuscular hemoglobin concentration, RDW – Red cell distribution

width, MPV – Mean platelet volume). n = 9 mice / group. Mean +/- SD. **b**) Flow cytometry analysis of blood samples from mice treated for 14 days with vehicle or **AS-99** (30 mg/kg, q.d., i.p.) to assess the effect on myeloid, B- and T-cells. Statistical significance calculated using unpaired 2-tailed t-test. n = 9 mice / group. Mean +/- SD. ns - not significant. (**c**) Representative gating strategy corresponding to the **Supplementary Figure 12b** for quantification of myeloid, B- and T-cells in blood samples of mice treated with vehicle or **AS-99**. SSC-A – side scatter area; FSC-A – forward scatter area; FSC-H – forward scatter height.

Supplementary Table 1. Data collection and refinement statistics for ASH1L-inhibitor complexes.

	**AS-5** (6X0P)	**AS-85** (6WZW)
**Data collection**		
Space group	P1	C121
Cell dimensions		
a, *b*, *c* (Å)	54.13, 62.31, 72.97	76.28, 32.00, 91.16
α, β, γ (°)	87.76, 85.55, 90.04	90.00, 102.34, 90.00
Resolution (Å)	40.86-1.69 (1.73-1.69)	37.33-1.69 (1.72-1.69)
R_{sym} or R_{merge}	0.096 (0.591)	0.105 (0.483)
$I / \sigma I$	14.4 (2.1)	14.9 (2.3)
Completeness (%)	95.8 (87.7)	99.8 (99.6)
Redundancy	3.9 (3.7)	3.7 (3.5)
**Refinement**		
Resolution (Å)	40.86-1.69	37.33-1.69
No. reflections	101290	24473
R_{work} / R_{free}	0.1773/0.2129	0.1639/0.2064
No. atoms		
Protein	7113	1815
Ligand	104	57
Water	1071	256
B-factors		
Protein	17.89	19.10
Ligand	16.35	26.59
Water	26.14	28.51
R.m.s. deviations		
Bond lengths (Å)	0.006	0.007
Bond angles (°)	0.90	1.00

The datasets were collected from single crystals.
Data for the highest-resolution shell is shown in parentheses.

Supplementary Table 2. Properties of ASH1L inhibitors. IC_{50} values measured in histone methyltransferase assay with ASH1L. LE (ligand efficiency), LLE (ligand-lipophilicity efficiency),[2] clogP and tPSA (topological polar surface area) were calculated using ChemDraw Professional 16 software.

Compound	IC_{50} (μM)	LE	LLE	clogP	tPSA
**2**	50.5	0.33	0.89	3.4	38.1
**3**	15.0	0.31	1.76	3.1	49.5
**4**	23.5	0.32	2.27	2.4	64.1
**AS-5**	4.0	0.32	3.38	2.0	75.5
**AS-6**	0.52	0.23	4.72	1.6	120.1
**AS-85**	0.60	0.24	3.91	2.3	107.8
**AS-99**	0.79	0.21	2.04	4.1	99.0

Supplementary Table 3. Assay conditions for selectivity experiments with a panel of histone methyltransferases. A buffer containing 50 mM Tris, pH = 8.5, 25 mM NaCl, 2 mM $MgCl_2$, 1 mM DTT and 0.01% Triton X-100 was used in experiments containing core histone. A buffer containing 50 mM Tris, pH 8.5, 1.5 mM $MgCl_2$, 1 mM TCEP and 0.01% Triton-X was used in experiments containing nucleosome.

Protein	[Protein] (nM)	Substrate	Concentration	
			Substrate	^{3}H-SAM (μM)
ASH1L	50	nucleosome	250 nM	1
DOT1L	20	nucleosome	250 nM	1
EZH2	50	core histone	0.05 mg/mL	1
G9a	5	core histone	0.05 mg/mL	1
GLP	5	core histone	0.05 mg/mL	1
MLL1	100	nucleosome	500 nM	1
MLL4	200	nucleosome	500 nM	1
NSD1	200	nucleosome	250 nM	1
NSD2	100	nucleosome	250 nM	1
NSD3	200	nucleosome	250 nM	1
PRDM9	10	core histone	0.05 mg/mL	1
PRMT1	100	core histone	0.05 mg/mL	1
PRMT3	20	core histone	0.05 mg/mL	1
PRMT4	50	core histone	0.05 mg/mL	1
PRMT6	100	core histone	0.05 mg/mL	1
SETD2	100	nucleosome	250 nM	1
SET7/9	10	core histone	0.05 mg/mL	1
SYMD1	25	core histone	0.05 mg/mL	1
SYMD2	50	core histone	0.05 mg/mL	1
SUV39H2	200	core histone	0.05 mg/mL	1

Chemical synthesis of ASH1L inhibitors

Abbreviations

aq.	aqueous
Boc	*tert*-butoxycarbonyl
calcd.	calculated
DBU	1,8-diazabicyclo[5.4.0]undec-7-ene
DCM	dichloromethane
DIPEA	*N*,*N*-diisopropylethylamine
DMF	*N*,*N*-dimethylformamide
DMSO	dimethylsulfoxide
DPPA	diphenylphosphoryl azide
ESI	electrospray ionization
EtOAc	ethyl acetate
EtOH	ethanol
h	hour(s)
HATU	1-[Bis(dimethylamino)methylene]-1*H*-1,2,3-triazolo[4,5-b]pyridinium 3-oxide hexafluorophosphate
HRMS	high-resolution mass spectrometry
LRMS	low-resolution mass spectrometry
MeCN	acetonitrile
MeOH	methanol
min	minute(s)
Ms	methanesulfonyl
NBS	*N*-Bromosuccinimide
NMR	nuclear magnetic resonance
STAB	Sodium triacetoxyborohydride
TBAF	Tetrabutylammonium fluoride
TBS	*tert*-butyldimethylsilyl
TEA	triethylamine
Tf	trifluoromethanesulfonyl

TFA	trifluoroacetic acid
THF	tetrahydrofuran
TLC	thin-layer chromatography
Ts	*p*-toluenesulfonyl
TTBP·HBF_4	tri-*tert*-butylphosphonium tetrafluoroborate
RT	room temperature

General Methods

All reagents were commercially available and used as received. Glassware was oven-dried before use for reactions run under anhydrous conditions. Analytical TLC was performed on Merck TLC aluminum plates precoated with F254 silica gel 60 (UV, 254 nm, and iodine). The NMR spectra were recorded on a Bruker Avance III instrument at 600 MHz for ^{1}H and 151 MHz for ^{13}C spectra. Chemical shifts are reported in ppm relative to tetramethylsilane or residual solvent signal. The following abbreviations were used to describe peak splitting patterns when appropriate: s = singlet, d = doublet, t = triplet, q = quartet, m = multiplet. Coupling constants, *J*, were reported in Hertz unit (Hz). The mass measurements were determined on a Micromass LCT time-of-flight mass spectrometer using positive mode and electrospray ionization. The exact mass measurements were determined on Agilent Q-TOF time-of-flight mass spectrometer using positive ion mode and electrospray ionization. The purity analysis of final compounds was determined on Shimadzu Prominence HPLC system (20 series: binary pump, UV/vis at 254 nm, heated column compartment 28 °C), using Restek Ultra C18 (5 μm) 150 mm × 4.6 mm column. LRMS was recorded on Shimadzu LC-2020 system (DUIS-ESI). The solvents were programmed to run at gradient starting 20% MeCN in water to 80%. If not indicated, the purity of all final compounds was >= 95% as determined by HPLC via integration of UV spectra at 254 nm.

Synthesis of 2

Synthetic pathway to prepare **2**.

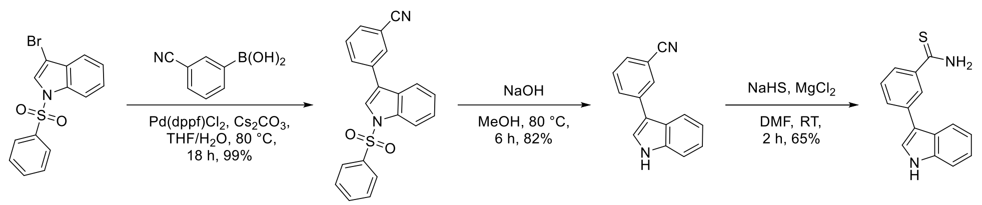

10 **11** **12** **2**

3-(1-(Phenylsulfonyl)-1*H*-indol-3-yl)benzonitrile (11). A mixture of 3-bromo-1-(phenylsulfonyl)-1*H*-indole (**10**, 500 mg, 1.487 mmol), (3-cyanophenyl)boronic acid (437 mg, 2.974 mmol), Pd(dppf)$Cl_2 \cdot$DCM complex (121 mg, 0.149 mmol), cesium carbonate (1454 mg, 4.462 mmol) in THF (7 mL) and water (0.7 mL) was stirred at 80 °C under argon atmosphere for 18 h. The mixture was cooled down to RT, partitioned by EtOAc (50 mL) and water (30 mL). The organic layer was concentrated *in vacuo*, and the residue was purified by column chromatography on silica gel (0~50% EtOAc in hexanes) to give the title compound (**11**, 530 mg, 1.479 mmol, 99% yield) as a white solid; ^{1}H NMR (600 MHz, DMSO-d_6) δ 8.31 (s, 1H), 8.23 (t, *J* = 1.9 Hz, 1H), 8.09 (dt, *J* = 8.2, 2.7 Hz, 3H), 8.03 (d, *J* = 8.4 Hz, 1H), 7.89 (d, *J* = 8.0 Hz, 1H), 7.86 – 7.81 (m, 1H), 7.70 (q, *J* = 7.9 Hz, 2H), 7.61 (t, *J* = 7.9 Hz, 2H), 7.44 (t, *J* = 7.8 Hz, 1H), 7.36 (t, *J* = 7.6 Hz, 1H).

3-(1*H*-Indol-3-yl)benzonitrile (12). A mixture of **11** (300 mg, 0.837 mmol) and sodium hydroxide (235 mg, 5.875 mmol) in MeOH (4 mL) was stirred at 80 °C for 6 h. The mixture was cooled down to RT and concentrated to remove MeOH. The residue was partitioned by EtOAc (50 mL) and water (30 mL). The organic layer was concentrated *in vacuo*, and the residue was purified by column chromatography on silica gel (0~30% EtOAc in hexanes) to give the title compound (**12**, 150 mg, 0.687 mmol, 82% yield) as a colorless oil; ^{1}H NMR (600 MHz, $CDCl_3$) δ 8.35 (s, 1H), 7.94 (d, *J* = 1.1 Hz, 1H), 7.92 – 7.85 (m, 2H), 7.66 – 7.61 (m, 1H), 7.55 (d, *J* = 1.4 Hz, 1H), 7.47 (dt, *J* = 8.1, 0.9 Hz, 1H), 7.43 (d, J = 2.6 Hz, 1H), 7.32 – 7.28 (m, 1H), 7.25 – 7.19 (m, 1H).

3-(1*H*-Indol-3-yl)benzothioamide (2). To a solution of **12** (20 mg, 0.092 mmol) in DMF (2 mL), was added sodium hydrosulfide hydrate (200 mg, 2.700 mmol) and magnesium chloride (200 mg, 2.101 mmol) at RT. Mixture was stirred at RT for 2 h. Water (20 mL), was added and product was extracted by EtOAc (2 × 20 mL). Organic phase was separated and evaporated. The crude was purified by column chromatography on silica gel (0~100% EtOAc in hexanes) to give the title

compound (**2**, 15 mg, 0.059 mmol, 65%) as a yellow solid; ^{1}H NMR (600 MHz, CD_3CN) δ 9.54 (s, 1H), 8.29 (s, 1H), 8.19 (t, *J* = 1.9 Hz, 1H), 8.12 (s, 1H), 7.94 (dd, *J* = 7.9, 1.2 Hz, 1H), 7.85 (ddd, J = 7.7, 1.9, 1.1 Hz, 1H), 7.78 (ddd, *J* = 7.8, 2.0, 1.1 Hz, 1H), 7.59 (d, *J* = 2.7 Hz, 1H), 7.53 – 7.42 (m, 2H), 7.25 – 7.21 (m, 1H), 7.20 – 7.15 (m, 1H); ^{13}C NMR (151 MHz, CD_3CN) δ 203.34, 140.76, 137.69, 136.55, 130.50, 129.28, 125.96, 125.90, 125.18, 123.99, 122.74, 120.81, 119.75, 116.51, 112.54; ESI HRMS (m/z): $[M+H]^+$ calcd. for $C_{15}H_{12}N_2S$ 253.0794; found 253.0793.

Synthesis of 3

Synthetic pathway to prepare **3**.

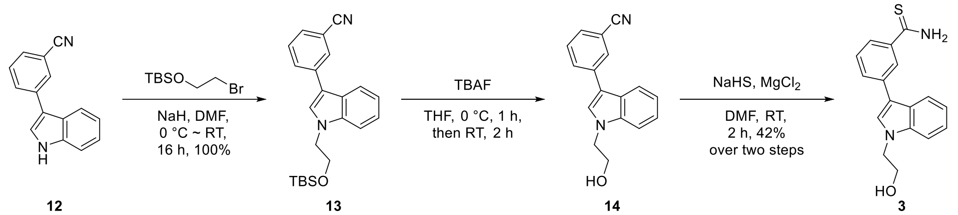

3-(1-(2-((*tert*-Butyldimethylsilyl)oxy)ethyl)-1*H*-indol-3-yl)benzonitrile (13). To a solution of **12** (87 mg, 0.399 mmol) in DMF (2 mL), was added 60% NaH (32 mg, 0.797 mmol) at 0 °C. The mixture was stirred at 0 °C for several minutes. Then, (2-bromoethoxy)(*tert*-butyl)dimethylsilane (130 µL, 0.598 mmol) was added to the mixture. The resulting mixture was stirred at RT for 16 h. EtOAc (20 mL) was added to dilute the mixture and water (20 mL) was added. The organic layer was separated, and aqueous layer was extracted with EtOAc (10 mL). The organic layers were combined and concentrated *in vacuo* to give crude product, which was purified by column chromatography on silica gel to give the title compound (150 mg, 0.398 mmol, 100% yield) as a colorless oil; ^{1}H NMR (600 MHz, $CDCl_3$) δ 8.01 – 7.83 (m, 2H), 7.63 – 7.48 (m, 2H), 7.41 (dd, *J* = 19.1, 11.0 Hz, 2H), 7.35 – 7.28 (m, 2H), 7.25 – 7.16 (m, 1H), 4.42 – 4.26 (m, 2H), 4.04 – 3.94 (m, 2H), 0.92 (s, 9H), 0.09 (s, 6H).

3-(1-(2-Hydroxyethyl)-1*H*-indol-3-yl)benzonitrile (14). TBAF (0.6 mmol, 0.6 mL of 1.0 M solution in THF) was added at 0 °C to a solution of **13** (150 mg, 0.398 mmol) in dry THF (3 mL), and the reaction mixture was kept under stirring at 0 °C for 1 h. The temperature was then raised to RT for 2 h. The reaction mixture was diluted with EtOAc (20 mL) and washed with water (20 mL). The organic layer was separated and concentrated *in vacuo*. The residue was purified by

column chromatography on silica gel (0~50% EtOAc in hexane) to give the title compound (**14**) as a colorless oil; ^{1}H NMR (600 MHz, DMSO-d_6) δ 8.09 – 8.07 (m, 1H), 8.04 (dt, *J* = 7.5, 1.7 Hz, 1H), 7.93 (dt, *J* = 8.0, 1.0 Hz, 1H), 7.90 (s, 1H), 7.68 – 7.62 (m, 2H), 7.59 (dt, *J* = 8.3, 0.9 Hz, 1H), 7.24 (ddd, *J* = 8.2, 7.0, 1.2 Hz, 1H), 7.17 (ddd, *J* = 8.0, 7.0, 1.0 Hz, 1H), 4.93 (t, *J* = 5.3 Hz, 1H), 4.28 (t, *J* = 5.6 Hz, 2H), 3.79 (q, *J* = 5.5 Hz, 2H).

3-(1-(2-Hydroxyethyl)-1*H*-indol-3-yl)benzothioamide (3). To a solution of **14** in the last step in DMF (3 mL), was added sodium hydrosulfide hydrate (210 mg, 2.835 mmol) and magnesium chloride (200 mg, 2.101 mmol) at RT. The mixture was stirred at RT for 2 h. Water (20 mL) was added to the mixture and the mixture was extracted by EtOAc (2 × 20 mL). Organic phase was separated and evaporated. The crude was purified by column chromatography on silica gel (0~100% EtOAc in hexanes) to give the title compound as a yellow solid (**3**, 50 mg, 0.169 mmol, 42% yield over two steps); ^{1}H NMR (600 MHz, CD_3OD) δ 8.25 (q, *J* = 1.5 Hz, 1H), 7.93 (d, *J* = 8.0 Hz, 1H), 7.80 (dt, *J* = 7.8, 1.4 Hz, 1H), 7.71 (dt, *J* = 7.9, 1.5 Hz, 1H), 7.58 (s, 1H), 7.48 (d, *J* = 8.3 Hz, 1H), 7.44 (t, *J* = 7.7 Hz, 1H), 7.25 – 7.20 (m, 1H), 7.16 – 7.13 (m, 1H), 4.32 (t, *J* = 5.6 Hz, 2H), 3.92 (t, *J* = 5.5 Hz, 2H); ^{13}C NMR (151 MHz, CD_3OD) δ 204.51, 141.84, 138.67, 137.39, 130.84, 129.46, 128.08, 127.49, 127.29, 124.90, 122.94, 121.10, 120.55, 116.75, 111.01, 62.11, 49.67; ESI HRMS (m/z): $[M+H]^+$ calcd. for $C_{17}H_{16}N_2OS$ 297.1056; found 297.1060.

Synthesis of 4

Synthetic pathway to prepare **4**.

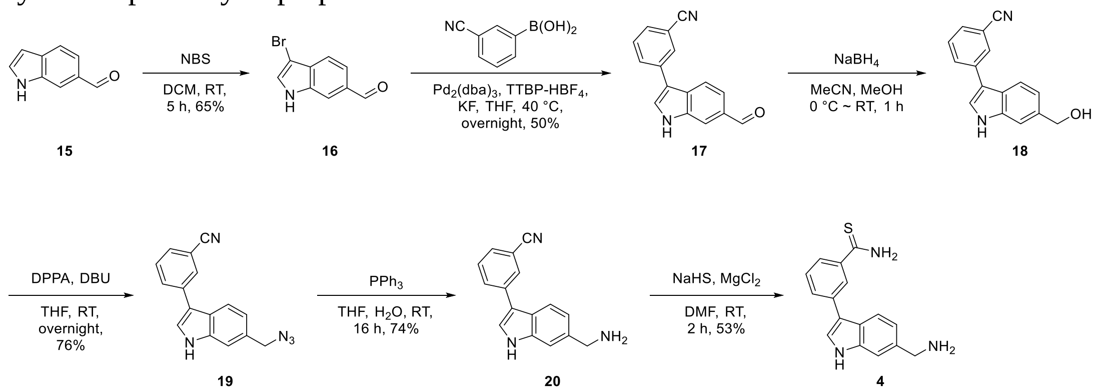

3-Bromo-1*H*-indole-6-carbaldehyde (16). To a mixture of 1*H*-indole-6-carbaldehyde (**15**, 10 g, 68.9 mmol) in DCM (600 mL), was added NBS (12.3 g, 69.1 mmol). The reaction mixture was stirred at RT for 5 h. Water was added to quench the reaction. The DCM phase was separated and

dried over anhydrous Na_2SO_4. After filtration and concentration, the crude product was obtained (**16**, 10 g, 44.6 mmol, 65% yield) as a purple solid; ^{1}H NMR (600 MHz, DMSO-d_6) δ 12.05 (s, 1H), 10.04 (s, 1H), 8.03 (d, *J* = 1.1 Hz, 1H), 7.88 (d, *J* = 2.7 Hz, 1H), 7.65 (dd, *J* = 8.3, 1.3 Hz, 1H), 7.57 (d, *J* = 8.3 Hz, 1H).

3-(6-Formyl-1*H*-indol-3-yl)benzonitrile (17). The mixture of **16** (2500 mg, 11.16 mmol), (3-cyanophenyl)boronic acid (3295 mg, 22.430 mmol), tris(dibenzylideneacetone) dipalladium (0) (1540 mg, 1.682 mmol), tri-*tert*-butylphosphonium tetrafluoroborate (976 mg, 3.364 mmol) and anhydrous KF (2605 mg, 44.836 mmol) in anhydrous THF (100 mL) was stirred at 40 °C overnight under argon atmosphere. The mixture was cooled down to RT, filtered through celite and washed by EtOAc (100 mL). The filtrate was concentrated *in vacuo*, and the residue was purified by column chromatography on silica gel (0~50% EtOAc in hexanes) to give the title compound (**17**, 1380 mg, 5.604 mmol, 50% yield) as a yellow solid; ^{1}H NMR (400 MHz, CD_3OD) δ 10.0 (s, 1H), 8.02 (m, 4H), 7.91 (m, 1H), 7.72 (dd, *J* = 8.0, 1.3 Hz, 1H), 7.62 (m, 2H).

3-(6-(Hydroxymethyl)-1*H*-indol-3-yl)benzonitrile (18). To a mixture of **17** (300 mg, 1.218 mmol) in MeOH (10 mL) and MeCN (10 mL), was added sodium borohydride (231 mg, 6.096 mmol) at 0 °C. The mixture was stirred at RT for another 1 h. Saturated NH_4Cl aq. solution (5 mL) was added to quench the reaction. After stirring for 15 min, the solvent MeOH was removed under reduced pressure. The product was extracted by EtOAc (2 × 20 mL). Organic phase was separated and evaporated to give crude product **18** in a quantitative yield, which was used in the next step directly; ^{1}H NMR (600 MHz, DMSO-d_6) δ 11.48 (s, 1H), 8.10 (s, 1H), 8.06 (dt, *J* = 7.3, 1.6 Hz, 1H), 7.88 – 7.84 (m, 2H), 7.66 – 7.60 (m, 2H), 7.43 (s, 1H), 7.10 (dd, *J* = 8.4, 1.5 Hz, 1H), 5.12 (t, *J* = 5.7 Hz, 1H), 4.61 (d, *J* = 5.8 Hz, 2H).

3-(6-(Azidomethyl)-1*H*-indol-3-yl)benzonitrile (19). To a 25-mL round-bottom flask equipped with a magnetic stirrer bar were added **18** (300 mg, 1.208 mmol), DPPA (339 µL, 1.573mmol), and THF (5 mL). After the mixture was stirred at 0 °C for 10 min, DBU (220 µL, 1.471 mmol) was added in one portion. The resulting mixture was stirred at RT overnight. The mixture was partitioned between EtOAc (20 mL) and water (20 mL). The combined organic layers were washed with water (10 mL × 2), dried over anhydrous Na_2SO_4, filtered, concentrated, and purified by column chromatography on silica gel (0~20% EtOAc in hexanes) to give the title compound as a pink oil (**19**, 250 mg, 76% yield); ^{1}H NMR (600 MHz, DMSO-d_6) δ 11.65 (s, 1H), 8.12 (t, *J* = 1.9

Hz, 1H), 8.07 (dt, J = 7.6, 1.7 Hz, 1H), 7.98 – 7.88 (m, 2H), 7.69 – 7.60 (m, 2H), 7.50 (s, 1H), 7.15 (dd, J = 8.4, 1.6 Hz, 1H), 4.55 (s, 2H).

3-(6-(Aminomethyl)-1*H*-indol-3-yl)benzonitrile (20). To a solution of **20** (30 mg, 0.110 mmol) in THF (2 mL), was added PPh_3 (115 mg, 0.438 mmol). The reaction mixture was stirred at RT for 1 h before adding water (0.2 mL). The mixture was stirred at RT for another 16 h. The mixture was partitioned by EtOAc and saturated $NaHCO_3$ aq. solution. The aqueous layer was extracted by EtOAc. The combined organic layers were dried over anhydrous Na_2SO_4, filtered, and concentrated *in vacuo*. The crude was purified by column chromatography on silica gel (0~40% MeOH (5% ammonia solution) in DCM) to give the title compound as a yellow solid (**20**, 20 mg, 0.081 mmol, 74% yield); ^{1}H NMR (600 MHz, DMSO-d_6) δ 11.54 (s, 1H), 8.18 (s, 1H), 8.15 (dt, J = 7.3, 1.9 Hz, 1H), 7.98 – 7.89 (m, 2H), 7.76 – 7.67 (m, 2H), 7.52 (s, 1H), 7.20 (d, J = 8.3 Hz, 1H), 3.94 (s, 2H); LRMS (ESI): *m/z* 231 $[M+H-NH_3]^+$.

3-(6-(Aminomethyl)-1*H*-indol-3-yl)benzothioamide (4). To a solution of **20** (20 mg, 0.081 mmol) in DMF (2 mL), was added sodium hydrosulfide hydrate (200 mg, 2.700 mmol) and magnesium chloride (200 mg, 2.101 mmol) at RT. Mixture was stirred at RT for 2 h. Water (20 mL) was added and product was extracted by EtOAc (2 × 20 mL). Organic phase was separated and evaporated. The crude was purified by column chromatography on silica gel (0~10% MeOH (10% ammonium hydroxide) in DCM) to give the title compound (**4**, 12 mg, 0.043 mmol, 53% yield) as a white solid; ^{1}H NMR (600 MHz, CD_3CN) δ 9.58 (s, 1H), 8.65 (s, 1H), 8.32 (s, 1H), 8.22 – 8.19 (m, 1H), 8.15 (s, 1H), 7.94 (d, J = 8.2 Hz, 1H), 7.87 (dt, J = 7.7, 1.5 Hz, 1H), 7.81 (dt, J = 7.7, 1.5 Hz, 1H), 7.62 (d, J = 2.6 Hz, 1H), 7.54 – 7.49 (m, 2H), 7.19 (dd, J = 8.3, 1.4 Hz, 1H), 4.93 (d, J = 5.6 Hz, 2H); ^{13}C NMR (151 MHz, CD_3CN) δ 203.31, 140.76, 137.74, 136.41, 131.73, 130.47, 129.31, 125.94, 125.35, 125.25, 124.41, 121.33, 119.92, 116.54, 112.24, 50.44; ESI HRMS (m/z): $[M+H-NH_3]^+$ calcd. for $C_{16}H_{15}N_3S$ 265.0794; found 265.0800.

Synthesis of 5 (AS-5)

Synthetic pathway to prepare **5 (AS-5)**.

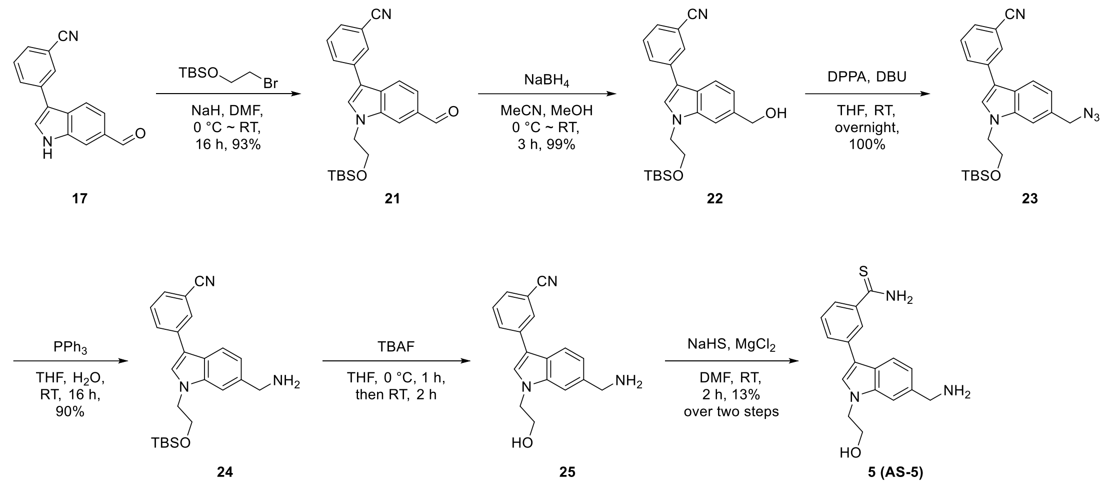

3-(1-(2-((*tert*-Butyldimethylsilyl)oxy)ethyl)-6-formyl-1*H*-indol-3-yl)benzonitrile (21). To a solution of **17** (210 mg, 0.853 mmol) in DMF (2 mL), was added 60% NaH (68 mg, 1.707 mmol) at 0 °C. The mixture was stirred at 0 °C for several minutes. Then, (2-bromoethoxy)(*tert*-butyl)dimethylsilane (275 µL, 1.280 mmol) was added to the mixture. The resulting mixture was stirred at RT for 16 h. The reaction mixture was partitioned between EtOAc (20 mL) and water (20 mL). The organic layer was separated, and aqueous layer was extracted with EtOAc (10 mL). The combined organic layers were concentrated under vacuo to give crude product, which was purified by column chromatography on silica gel (0~20% EtOAc in hexanes) to give the title compound (**21**, 320 mg, 0.791 mmol, 93% yield) as yellow oil; ^{1}H NMR (600 MHz, DMSO-d_6) δ 10.06 (s, 1H), 8.23 (d, *J* = 1.4 Hz, 1H), 8.19 (s, 1H), 8.09 – 8.06 (m, 2H), 8.04 (ddd, *J* = 7.8, 1.9, 1.2 Hz, 1H), 7.72 (dt, *J* = 7.7, 1.4 Hz, 1H), 7.70 – 7.65 (m, 2H), 4.47 (t, *J* = 5.0 Hz, 2H), 3.99 – 3.95 (m, 2H), 0.69 (s, 9H), -0.24 (s, 6H).

3-(1-(2-((*tert*-Butyldimethylsilyl)oxy)ethyl)-6-(hydroxymethyl)-1*H*-indol-3-yl)benzonitrile (22). To a mixture of **21** (320 mg, 0.791 mmol) in MeOH (30 mL), was added sodium borohydride (150 mg, 3.965 mmol) at 0 °C. The mixture was stirred at RT for 3 h. Saturated NH_4Cl aq. solution (5 mL) was added to quench the reaction. After stirring for 5 min, MeOH was removed under reduced pressure. The product was extracted by EtOAc (2 × 20 mL). Organic phase was separated, dried and evaporated to give crude product (**22**, 320 mg, 0.787 mmol, 99%), which was used in the next step directly; ^{1}H NMR (600 MHz, DMSO-d_6) δ 8.05 – 7.99 (m, 2H), 7.86 (d, *J* = 8.3 Hz, 1H), 7.83 (s, 1H), 7.67 – 7.60 (m, 2H), 7.50 (d, *J* = 1.3 Hz, 1H), 7.13 (dd, *J* = 8.3, 1.4 Hz, 1H),

5.13 (t, *J* = 5.6 Hz, 1H), 4.62 (d, *J* = 5.6 Hz, 2H), 4.31 (t, *J* = 5.3 Hz, 2H), 3.93 (t, *J* = 5.3 Hz, 2H), 0.76 (s, 9H), -0.18 (s, 6H).

3-(6-(Azidomethyl)-1-(2-((*tert*-butyldimethylsilyl)oxy)ethyl)-1*H*-indol-3-yl)benzonitrile (23). To a 25-mL round-bottom flask equipped with a magnetic stirrer bar, were added **22** (320 mg, 0.787 mmol), DPPA (270 µL, 1.253 mmol), and THF (4 mL). After the mixture was stirred at 0 °C for 10 min, DBU (176 µL, 1.177 mmol) was added in one portion. The resulting mixture was stirred at RT overnight. Saturated $NaHCO_3$ aq. solution (5 mL) was added to the mixture. The organic layer was separated, and the aqueous layer was extracted with EtOAc (10 mL ×3). The combined organic layers were washed with water (10 mL × 2), dried over anhydrous Na_2SO_4, filtered, and concentrated *in vacuo*. The crude was purified by column chromatography on silica gel (0~20% EtOAc in hexanes) to give the title compound as a colorless oil (**23**, 340 mg, 0.787 mmol, 100%); ^{1}H NMR (600 MHz, $CDCl_3$) δ 8.02 – 7.82 (m, 2H), 7.70 – 7.45 (m, 3H), 7.46 – 7.34 (m, 2H), 7.22 – 7.08 (m, 1H), 4.67 – 4.40 (m, 2H), 3.96 – 4.05 (m, 2H), 0.81 (s, 9H), -0.13 (s, 6H).

3-(6-(Aminomethyl)-1-(2-((*tert*-butyldimethylsilyl)oxy)ethyl)-1*H*-indol-3-yl)benzonitrile (24). To a solution of **23** (340 mg, 0.787 mmol) in THF/H_2O (2 mL/0.2 mL) was added PPh_3 (827 mg, 3.153 mmol). The reaction mixture was stirred at RT for 16 h. The mixture was quenched with 1 N NaOH and extracted with EtOAc (100 mL). The organic layer was separated and dried over anhydrous Na_2SO_4. After filtration and concentration, the product was purified by column chromatography on silica gel (0~20% MeOH (10% ammonia) in DCM) to give the title compound (288 mg, 0.709 mmol, 90% yield) as a colorless oil; ^{1}H NMR (600 MHz, DMSO-d_6) δ 8.04 – 7.99 (m, 2H), 7.88 – 7.82 (m, 2H), 7.66 – 7.60 (m, 3H), 7.54 (s, 1H), 7.15 (dd, *J* = 8.3, 1.4 Hz, 1H), 4.31 (t, *J* = 5.3 Hz, 2H), 3.94 (t, *J* = 5.3 Hz, 2H), 3.88 (s, 2H), 0.76 (s, 9H), -0.18 (s, 6H); LRMS (ESI): *m/z* 389 $[M+H-NH_3]^+$.

3-(6-(Aminomethyl)-1-(2-hydroxyethyl)-1*H*-indol-3-yl)benzonitrile (25). 1.0 M TBAF in THF (0.6 mL, 0.6 mmol) was added to a solution of **24** (160 mg, 0.355 mmol) in dry THF (3 mL) at 0 °C, and the reaction mixture was kept under stirring at 0 °C for 1 h. The temperature was then raised to RT over 2 h. The reaction mixture was diluted with EtOAc (20 mL) and washed with water (20 mL). The organic layer was separated and concentrated *in vacuo*. The crude product **25** was used in the next step directly; ^{1}H NMR (600 MHz, DMSO-d_6) δ 8.05 (d, *J* = 5.1 Hz, 1H), 7.86

– 7.77 (m, 3H), 7.65 – 7.58 (m, 2H), 7.43 (s, 1H), 7.14 (d, *J* = 8.2 Hz, 1H), 4.19 – 4.27 (m, 4H), 3.78 (q, *J* = 6.2 Hz, 2H); LRMS (ESI): *m/z* 275 $[M+H-NH_3]^+$.

3-(6-(Aminomethyl)-1-(2-hydroxyethyl)-1*H*-indol-3-yl)benzothioamide (5, AS-5). To a solution of **25** in the last step in DMF (3 mL), was added sodium hydrosulfide hydrate (210 mg, 0.284 mmol) and magnesium chloride (200 mg, 0.210 mmol) at RT. The mixture was stirred at RT for 2 h. Water (20 mL) was added and product was extracted by EtOAc (2 × 20 mL). The organic phase was separated and evaporated. The crude was purified by column chromatography on silica gel (0~40% MeOH (10% ammonia) in DCM) to give the title compound as a yellow solid (**5**, **AS-5**, 15 mg, 0.046 mmol, 13% yield over two steps); ^{1}H NMR (600 MHz, CD_3OD) δ 8.25 (d, *J* = 1.8 Hz, 1H), 7.90 (d, *J* = 8.2 Hz, 1H), 7.79 (d, *J* = 7.7 Hz, 1H), 7.73 – 7.67 (m, 1H), 7.58 (s, 1H), 7.47 (s, 1H), 7.43 (t, *J* = 7.7 Hz, 1H), 7.15 (d, *J* = 8.3 Hz, 1H), 4.33 (t, *J* = 5.5 Hz, 2H), 3.96 (s, 2H), 3.93 (t, *J* = 5.5 Hz, 2H); ^{13}C NMR (151 MHz, CD_3OD) δ 204.48, 141.84, 138.89, 137.31, 136.56, 130.75, 129.48, 128.35, 127.31, 126.68, 124.84, 121.21, 120.81, 116.73, 110.00, 62.15, 49.65, 47.03; ESI HRMS (m/z): $[M+H]^+$ calcd. for $C_{18}H_{19}N_3OS$ 326.1322; found 326.1325.

Synthesis of 6 (AS-6)

Synthetic pathway to prepare **6 (AS-6)**.

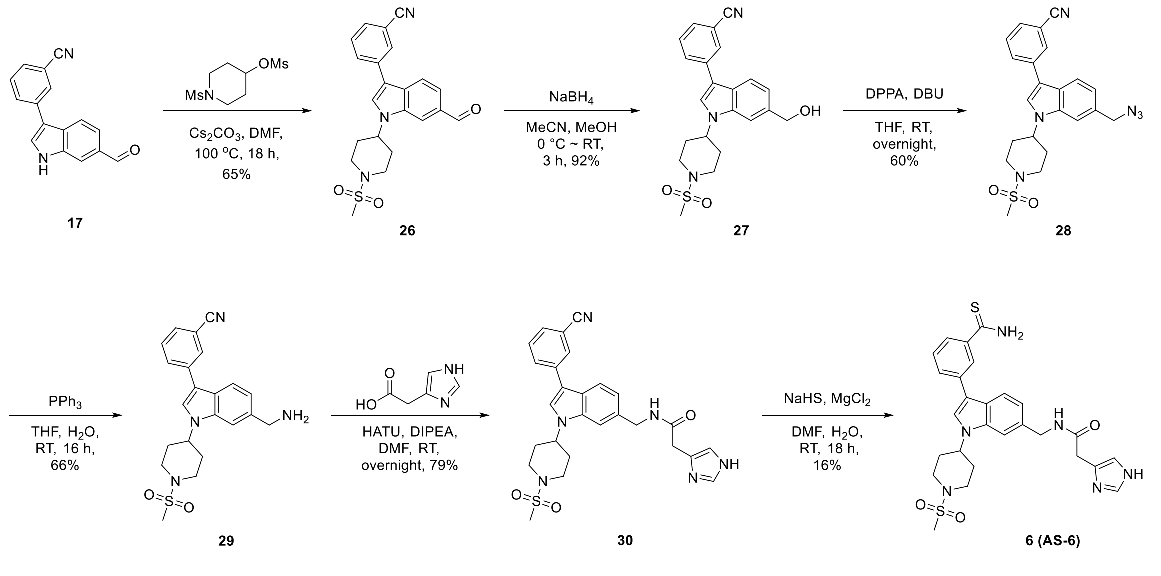

3-(6-Formyl-1-(1-(methylsulfonyl)piperidin-4-yl)-1*H*-indol-3-yl)benzonitrile (26). To an anhydrous DMF solution (5 mL) of **17** (50 mg, 0.203 mmol), was added cesium carbonate (199

mg, 0.610 mmol) and stirred at RT for 5 min. 1-(Methylsulfonyl)piperidin-4-yl methanesulfonate (209 mg, 0.812 mmol) was added into the mixture. The mixture was stirred at 100 °C for 18 h. Water (15 mL) was added to the mixture and product was extracted by EtOAc (2 × 20 mL). Organic phase was separated and evaporated to give crude product, which was purified by column chromatography on silica gel (0~100% EtOAc in hexanes) to give the title compound (**26**, 54 mg, 0.133 mmol, 65% yield)as yellow solid; ^{1}H NMR (600 MHz, Acetone-d_6) δ 10.10 (s, 1H), 8.34 (s, 1H), 8.26 (s, 1H), 8.19 – 8.10 (m, 3H), 7.77 (dd, *J* = 8.3, 1.4 Hz, 1H), 7.72 – 7.65 (m, 2H), 4.94 (tt, *J* = 11.5, 4.6 Hz, 1H), 3.97 (dp, *J* = 12.6, 2.2 Hz, 2H), 3.20 (td, *J* = 12.0, 3.1 Hz, 2H), 2.93 (s, 3H), 2.32 (dqd, *J* = 24.4, 12.2, 10.8, 3.8 Hz, 4H).

3-(6-(Hydroxymethyl)-1-(1-(methylsulfonyl)piperidin-4-yl)-1*H*-indol-3-yl)benzonitrile (27). To a mixture of **26** (54 mg, 0.133 mmol) in MeOH (10 mL) and MeCN (20 mL), was added sodium borohydride (25 mg, 0.663 mmol) at 0 °C. The mixture was stirred at RT for 3 h. Saturated NH_4Cl aq. solution (5 mL) was added to quench the reaction. After stirring for 5 min, MeOH was removed under reduced pressure. The product was extracted by EtOAc (2 × 20 mL). Organic phase was separated, dried and evaporated to give crude product (**27**, 50 mg, 0.122 mmol, 92%), which was used in the next step directly; ^{1}H NMR (600 MHz, CD_3OD) δ 7.89 (dd, *J* = 6.4, 1.7 Hz, 2H), 7.75 (d, *J* = 8.3 Hz, 1H), 7.67 (s, 1H), 7.51 – 7.44 (m, 3H), 7.13 – 7.08 (m, 1H), 4.66 (s, 2H), 4.50 (tt, *J* = 10.9, 6.5 Hz, 1H), 3.89 – 3.82 (m, 2H), 2.99 (td, *J* = 12.1, 5.6 Hz, 2H), 2.84 (s, 3H), 2.11 (td, *J* = 11.0, 9.7, 3.6 Hz, 4H).

3-(6-(Azidomethyl)-1-(1-(methylsulfonyl)piperidin-4-yl)-1*H*-indol-3-yl)benzonitrile (28). To a 25-mL round-bottom flask equipped with a magnetic stirrer bar, were added **27** (50 mg, 0.122 mmol), DPPA (34 µL, 0.159 mmol), and THF (2 mL). After the mixture was stirred at 0 °C for 10 min, DBU (22 µL, 0.147 mmol) was added to the mixture in one portion. The resulting mixture was stirred at RT overnight. Saturated $NaHCO_3$ aq. solution (5 mL) was added. The organic layer was separated, and the aqueous layer was extracted with EtOAc (10 mL ×3). The combined organic layers were washed with water (10 mL × 2), dried over anhydrous Na_2SO_4, filtered, concentrated, and purified by column chromatography on silica gel (0~100% EtOAc in hexanes) to give the title compound (32 mg, 0.074 mmol, 60% yield) as a colorless oil; ^{1}H NMR (600 MHz, Acetone-d_6) δ 8.18 – 8.07 (m, 3H), 8.03 (d, *J* = 8.2 Hz, 1H), 7.73 (d, *J* = 1.5 Hz, 1H), 7.69 – 7.61 (m, 2H), 7.27 (dd, *J* = 8.2, 1.5 Hz, 1H), 4.83 – 4.74 (m, 1H), 4.58 (s, 2H), 3.97 (dt, *J* = 12.1, 2.4 Hz, 2H), 3.17 (td, *J* = 12.1, 3.1 Hz, 2H), 2.94 (s, 3H), 2.36 – 2.22 (m, 4H).

3-(6-(Aminomethyl)-1-(1-(methylsulfonyl)piperidin-4-yl)-1*H*-indol-3-yl)benzonitrile (29). To a solution of the **28** (32 mg, 0.074 mmol) in mixed solution of THF (2 mL) and H_2O (0.2 mL), was added PPh_3 (58 mg, 0.221 mmol). The reaction mixture was stirred at RT for 16 h. The mixture was acidified to pH = 1 with 1 N HCl and extracted with EtOAc (100 mL). The aq. layer was separated and basified to pH = 10 with 1N NaOH. The resulting solution was extracted with EtOAc (30 mL). The organic layer was separated and dried over anhydrous Na_2SO_4. After filtration and concentration, the product was obtained as a white solid (**29**, 20 mg, 0.049 mmol, 66% yield); ^{1}H NMR (600 MHz, DMSO-d_6) δ 8.20 – 8.04 (m, 3H), 7.86 (d, *J* = 8.2 Hz, 1H), 7.70 – 7.59 (m, 3H), 7.16 (d, *J* = 8.2 Hz, 1H), 4.62 (td, *J* = 10.5, 5.6 Hz, 1H), 3.86 (s, 2H), 3.82 – 3.74 (m, 2H), 3.06 (td, *J* = 11.9, 3.9 Hz, 2H), 2.97 (s, 3H), 2.21 – 2.06 (m, 4H); LRMS (ESI): *m/z* 392 $[M+H-NH_3]^+$.

***N*-((3-(3-cyanophenyl)-1-(1-(methylsulfonyl)piperidin-4-yl)-1*H*-indol-6-yl)methyl)-2-(1*H*-imidazol-4-yl)acetamide (30).** The mixture of **29** (20 mg, 0.049 mmol), 4-imidazoleacetic acid hydrochloride (16 mg, 0.098 mmol), HATU (37 mg, 0.098 mmol) and DIPEA (26 µL, 0.147 mmol) in THF (2 mL) was stirred at RT overnight. The reaction mixture was partitioned between EtOAc (30 mL) and water (20 mL). The aqueous layer was extracted with EtOAc (10 mL). The organic layers were combined and washed with water and brine. After concentration, the crude was purified by column chromatography on silica gel (0~10% MeOH in DCM) to give the title compound (**30**, 20 mg, 0.039 mmol, 79% yield) as a white solid; ^{1}H NMR (600 MHz, DMSO-d_6) δ 11.88 (br. s., 1H), 8.37 (br. s., 1H), 8.09 - 8.18 (m, 1H), 8.06 (d, *J* = 6.24 Hz, 1H), 7.88 (d, *J* = 7.70 Hz, 1H), 7.60 - 7.69 (m, 1H), 7.56 (br. s., 1H), 7.50 (br. s., 1H), 7.11 (d, *J* = 8.07 Hz, 1H), 6.90 (br. s., 1H), 4.55 (br. s., 1H), 4.42 (d, *J* = 5.50 Hz, 2H), 4.02 - 4.09 (m, *J* = 5.14 Hz, 1H), 3.79 (d, *J* = 11.00 Hz, 2H), 3.17 (d, *J* = 5.14 Hz, 2H), 3.06 (t, *J* = 11.37 Hz, 3H), 2.98 (s, 3H), 2.03 - 2.19 (m, 4H); LRMS (ESI): *m/z* 517 $[M+H]^+$.

***N*-((3-(3-carbamothioylphenyl)-1-(1-(methylsulfonyl)piperidin-4-yl)-1*H*-indol-6-yl)methyl)-2-(1*H*-imidazol-4-yl)acetamide (6, AS-6)).** To a solution of **30** (20 mg, 0.039 mmol) in DMF (2 mL) and water (0.1 mL), was added sodium hydrosulfide hydrate (180 mg, 2.430 mmol) and magnesium chloride (200 mg, 2.100 mmol) at RT. The mixture was stirred at RT for 18 h. The reaction mixture was partitioned by EtOAc (30 mL) and water (20 mL). The aqueous layer was extracted with EtOAc (10 mL). The organic layer was combined and washed with water and brine. After concentration, the crude was purified by column chromatography on silica gel (0~8% MeOH in DCM) to give the title compound (**6**, **AS-6**, 3.5 mg, 0.0063 mmol, 16% yield) as a pale yellow

solid; ^{1}H NMR (600 MHz, Acetone-d_6) δ 9.00 (br. s., 1H), 8.89 (br. s., 1H), 8.31 (s, 1H), 7.79 - 7.92 (m, 5H), 7.62 (s, 1H), 7.43 - 7.52 (m, 2H), 7.12 (d, *J* = 8.44 Hz, 1H), 7.00 (br. s., 1H), 4.58 - 4.66 (m, 1H), 4.54 (d, *J* = 5.87 Hz, 2H), 3.94 (d, *J* = 12.10 Hz, 2H), 3.52 (s, 2H), 3.11 - 3.18 (m, 2H), 2.93 (s, 4H), 2.18 - 2.26 (m, 4H); ^{13}C NMR (151 MHz, Acetone-d_6) δ 203.3, 170.4, 141.5, 141.5, 137.9, 136.8, 136.2, 134.6, 130.4, 129.4, 126.4, 126.1, 125.5, 124.1, 121.3, 120.6, 117.0, 109.8, 55.0, 53.5, 46.4, 44.2, 35.2, 32.9; ESI HRMS (m/z): $[M+H]^+$ calcd. for $C_{27}H_{30}N_6O_3S_2$ 551.1894; found 551.1895.

Synthesis of 7 (AS-85) and 8 (AS-99)

Synthetic pathway to prepare **7 (AS-85)** and **8 (AS-99)**.

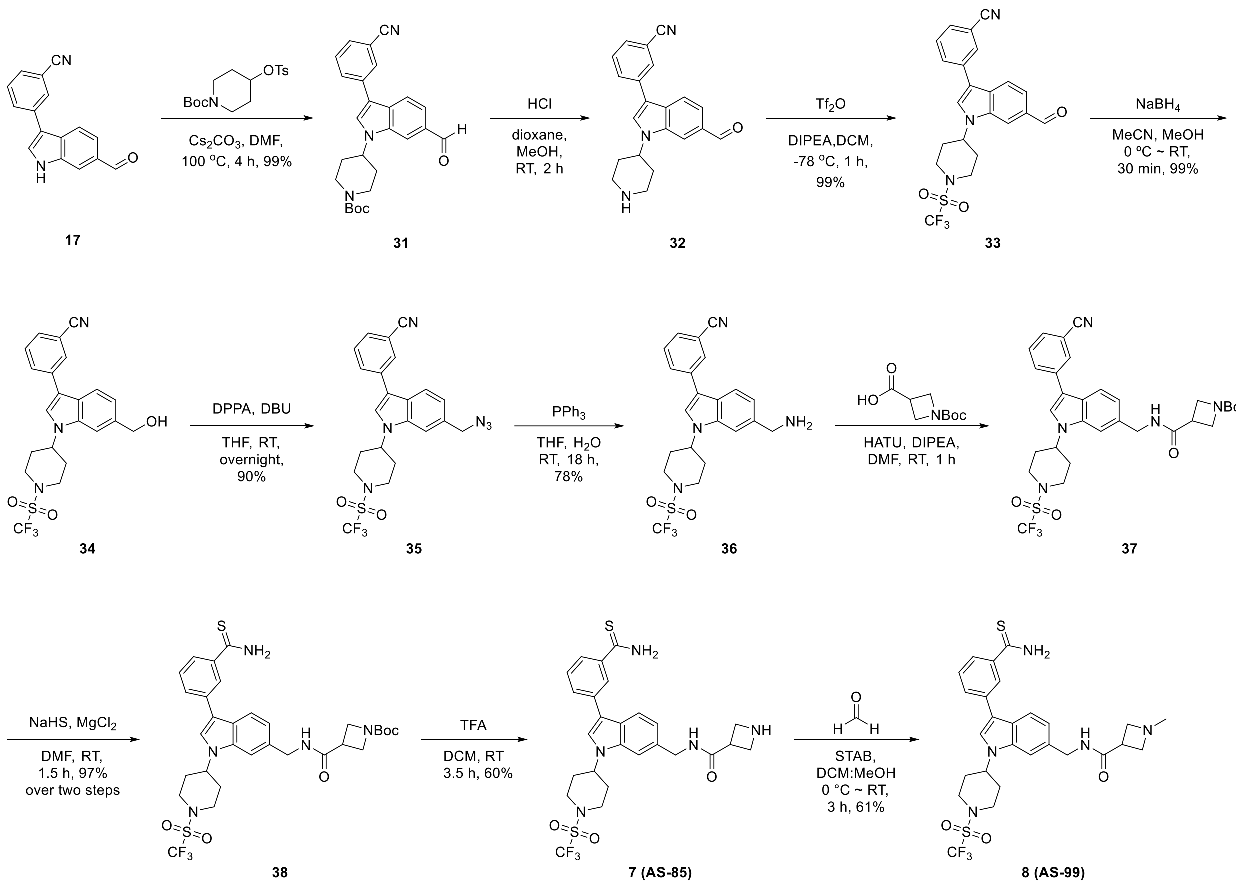

***tert*-Butyl 4-(3-(3-cyanophenyl)-6-formyl-1*H*-indol-1-yl)piperidine-1-carboxylate (31).** To an anhydrous DMF solution (20 mL) of **17** (1500 mg, 6.091 mmol) was added cesium carbonate (5957 mg, 18.283 mmol) and stirred at 0 °C for 15 min. Then, *tert*-butyl 4-(tosyloxy)piperidine-1-

carboxylate (8700 mg, 24.476 mmol) was added into the above mixture. The mixture was stirred at 100 °C for 4 h followed by cooling to RT. Water (15 mL) was added and product was extracted by EtOAc (2 × 50 mL). Organic phase was separated and evaporated to give crude product, which was purified by column chromatography on silica gel (0~30% EtOAc in hexanes) to give the title compound (**31**, 2580 mg, 6.007 mmol, 99% yield) as a yellow oil; ^{1}H NMR (600 MHz, CD_3OD) δ 10.05 (s, 1H), 8.23 (s, 1H), 8.06 (s, 1H), 8.04 – 7.97 (m, 3H), 7.75 (d, *J* = 8.4 Hz, 1H), 7.63 (d, *J* = 5.2 Hz, 2H), 4.76 – 4.74 (m, 1H), 4.33 (d, *J* = 13.5 Hz, 2H), 3.10 (s, 2H), 2.19 – 2.11 (m, 2H), 2.06 (td, *J* = 12.4, 4.3 Hz, 2H), 1.50 (s, 9H).

3-(6-Formyl-1-(piperidin-4-yl)-1*H*-indol-3-yl)benzonitrile (32). To a 250-mL round-bottom flask with **31** (2300 mg, 5.355 mmol) at RT followed by MeOH (50 mL). 4 N HCl in dioxane (16 mL) was added into the above mixture. The resulting mixture was stirred at RT for 2 h. The mixture was concentrated under vacuo to give product **32** (1600 mg, 4.373 mmol, 82% yield) as pale brown solid which was used in the next step directly; ^{1}H NMR (600 MHz, D_2O) δ 9.83 (s, 1H), 8.00 (s, 1H), 7.84 (d, *J* = 1.3 Hz, 1H), 7.72 (d, *J* = 7.9 Hz, 1H), 7.61 (d, *J* = 8.9 Hz, 2H), 7.56 (dt, *J* = 7.9, 1.4 Hz, 1H), 7.50 – 7.46 (m, 2H), 4.70 – 4.65 (m, 1H), 3.71 – 3.65 (m, 2H), 3.36 – 3.29 (m, 2H), 2.33 (d, *J* = 13.8 Hz, 2H), 2.23 (qd, *J* = 14.0, 13.5, 4.1 Hz, 2H).

3-(6-Formyl-1-(1-((trifluoromethyl)sulfonyl)piperidin-4-yl)-1*H*-indol-3-yl)benzonitrile (33). To the suspension of **32** (1600 mg, 4.373 mmol) in DCM (100 mL) was added DIPEA (2285 µL, 13.118 mmol) until the mixture was homogeneous. To the mixture, was added Tf_2O (883 µL, 5.247 mmol) at -78 °C. The reaction mixture was stirred at -78 °C for 1 h. Water and saturated $NaHCO_3$ aq. solution was added to quench the reaction. DCM layer was separated and washed with water. The organic layer was dried over anhydrous Na_2SO_4, filtered and concentrated to give crude product **33** (2000 mg, 4.334 mmol, 99% yield) which was used in the next step directly; ^{1}H NMR (600 MHz, DMSO-d_6) δ 10.08 (d, *J* = 2.7 Hz, 1H), 8.49 (d, *J* = 2.7 Hz, 1H), 8.34 (s, 1H), 8.19 (s, 1H), 8.13 – 8.08 (m, 2H), 7.73 (d, *J* = 8.4 Hz, 2H), 7.67 (td, *J* = 7.7, 2.6 Hz, 1H), 5.12 – 4.88 (m, 1H), 4.27 – 3.90 (m, 2H), 3.55 (t, *J* = 12.5 Hz, 2H), 2.18 (d, *J* = 20.3 Hz, 4H).

3-(6-(Hydroxymethyl)-1-(1-((trifluoromethyl)sulfonyl)piperidin-4-yl)-1*H*-indol-3-yl)benzonitrile (34). To a mixture of **33** (2000 mg, 4.334 mmol) in MeOH (500 mL) and MeCN (500 mL), was added sodium borohydride (820 mg, 21.668 mmol) at 0 °C. The mixture was stirred at RT for 30 min. Saturated NH_4Cl aq. solution (15 mL) was added to quench the reaction. After stirring for 10 min, the solvent was removed under reduced pressure. The product was extracted

by EtOAc (2 × 100 mL). The organic phase was separated, dried over anhydrous Na_2SO_4, and evaporated to give crude product **34** (2000 mg, 4.315 mmol, 99% yield) as a pale yellow solid which was used in the next step directly; ^{1}H NMR (600 MHz, DMSO-d_6) δ 8.14 (s, 2H), 8.08 (dd, *J* = 7.4, 2.1 Hz, 1H), 7.90 (d, *J* = 8.3 Hz, 1H), 7.72 – 7.57 (m, 3H), 7.16 (d, *J* = 8.3 Hz, 1H), 5.17 (t, *J* = 5.6 Hz, 1H), 4.87 – 4.76 (m, 1H), 4.65 (d, *J* = 5.5 Hz, 2H), 4.08 – 3.94 (m, 2H), 3.55 (t, *J* = 13.5 Hz, 2H), 2.14 (dt, *J* = 15.1, 7.3 Hz, 4H).

3-(6-(Azidomethyl)-1-(1-((trifluoromethyl)sulfonyl)piperidin-4-yl)-1*H*-indol-3-yl)benzonitrile (35). To a 25-mL round-bottom flask equipped with a magnetic stirrer bar were added **34** (2000 mg, 4.315 mmol), DPPA (2.0 mL, 9.281 mmol), and THF (20 mL). After the mixture was stirred at 0 °C for 10 min, DBU (2.125 mL, 14.209 mmol) was added in one portion. The resulting mixture was stirred at RT overnight. Saturated $NaHCO_3$ aq. solution (5 mL) was added. The organic layer was separated, and the aqueous layer was extracted with EtOAc (40 mL ×2). The combined organic layers were washed with water (10 mL × 2), dried over anhydrous Na_2SO_4, filtered, and concentrated. The crude was purified by column chromatography on silica gel (0~50% EtOAc in hexanes) to give the title compound (**35**, 1900 mg, 3.890 mmol, 90%) as a pale yellow solid; ^{1}H NMR (600 MHz, DMSO-d_6) δ 8.22 (s, 1H), 8.16 (s, 1H), 8.09 (dd, *J* = 7.8, 1.7 Hz, 1H), 7.98 (d, *J* = 8.2 Hz, 1H), 7.72 (s, 1H), 7.70 – 7.60 (m, 2H), 7.21 (dd, *J* = 8.1, 1.5 Hz, 1H), 4.85 (tt, *J* = 10.7, 4.5 Hz, 1H), 4.58 (s, 2H), 4.16 – 3.76 (m, 2H), 3.53 (t, *J* = 12.2 Hz, 2H), 2.24 – 2.05 (m, 4H).

3-(6-(Aminomethyl)-1-(1-((trifluoromethyl)sulfonyl)piperidin-4-yl)-1*H*-indol-3-yl)benzonitrile (36). To a solution of the **35** (1900 mg, 3.890 mmol) in THF (20 mL) and H_2O (2 mL) was added PPh_3 (5000 mg, 19.058 mmol). The reaction mixture was stirred at RT for 18 h. The mixture was partitioned with EtOAc (100 mL) and saturated $NaHCO_3$ aq. solution (50 mL). The aqueous layer was extracted with EtOAc (30 mL). The organic layer was combined and concentrated. The crude was purified by column chromatography on silica gel (0 ~ 20% MeOH (10% ammonia solution) in DCM) to give the title compound (**36**, 1400 mg, 3.027 mmol, 78% yield) as a yellow solid; ^{1}H NMR (600 MHz, DMSO-d_6) δ 8.13 (d, *J* = 7.2 Hz, 2H), 8.08 (d, *J* = 7.3 Hz, 1H), 7.87 (d, *J* = 8.2 Hz, 1H), 7.72 – 7.61 (m, 3H), 7.18 (d, *J* = 8.3 Hz, 1H), 4.80 (td, *J* = 11.0, 5.2 Hz, 1H), 4.01 (d, *J* = 12.6 Hz, 2H), 3.87 (s, 2H), 3.53 (t, *J* = 12.6 Hz, 2H), 2.28 – 2.05 (m, 4H); LRMS (ESI): *m/z* 446 $[M+H-NH_3]^+$.

***tert*-Butyl 3-(((3-(3-cyanophenyl)-1-(1-((trifluoromethyl)sulfonyl)piperidin-4-yl)-1*H*-indol-6-yl)methyl)carbamoyl)azetidine-1-carboxylate (37).** The mixture of **36** (1400 mg, 3.027 mmol), 1-(*tert*-butoxycarbonyl)azetidine-3-carboxylic acid (1218 mg, 6.054 mmol), HATU (2302 mg, 6.054 mmol) and DIPEA (1600 µL, 9.185 mmol) in DMF (10 mL) was stirred at RT for 1 h. The reaction mixture was partitioned between EtOAc (30 mL) and water (20 mL). The aqueous layer was extracted with EtOAc (10 mL). The organic layers were combined, washed with water and brine, and concentrated to give crude product **37**. The crude was used directly in the next step; ^{1}H NMR (600 MHz, DMSO-d_6) δ 8.48 (d, *J* = 6.0 Hz, 1H), 8.15 (dd, *J* = 14.9, 4.6 Hz, 2H), 8.10 – 8.06 (m, 1H), 7.93 – 7.90 (m, 1H), 7.71 – 7.60 (m, 2H), 7.55 (d, *J* = 5.0 Hz, 1H), 7.14 – 7.07 (m, 1H), 4.43 (t, *J* = 5.4 Hz, 2H), 3.97 (dd, *J* = 56.8, 24.1 Hz, 8H), 2.15 (s, 4H), 1.38 (d, *J* = 4.3 Hz, 9H); LRMS (ESI): *m/z* 668 $[M+Na]^+$.

***tert*-Butyl 3-(((3-(3-carbamothioylphenyl)-1-(1-((trifluoromethyl)sulfonyl)piperidin-4-yl)-1*H*-indol-6-yl)methyl)carbamoyl)azetidine-1-carboxylate (38).** To a solution of **37** in the last step in DMF (15 mL), was added sodium hydrosulfide hydrate (1200 mg, 16.201 mmol) and magnesium chloride (1200 mg, 12.608 mmol) at RT. The mixture was stirred at RT for 1.5 h. Water (20 mL) was added to the mixture and product was extracted by EtOAc (2 × 50 mL). The combined organic layers were dried over anhydrous Na_2SO_4. After filtration and evaporation, the crude was purified by column chromatography on silica gel (0~100% EtOAc in hexanes) to give the title compound (**38**, 2000 mg, 2.942 mmol, 97% yield over two steps) as a yellow oil; ^{1}H NMR (600 MHz, DMSO-d_6) δ 9.89 (s, 1H), 9.53 (s, 1H), 8.47 (t, *J* = 5.7 Hz, 1H), 8.18 (t, *J* = 1.9 Hz, 1H), 8.00 (s, 1H), 7.90 (d, *J* = 8.2 Hz, 1H), 7.85 – 7.81 (m, 1H), 7.74 (ddd, *J* = 7.8, 1.9, 1.0 Hz, 1H), 7.54 (d, *J* = 1.3 Hz, 1H), 7.47 (t, *J* = 7.8 Hz, 1H), 7.11 (dd, *J* = 8.3, 1.4 Hz, 1H), 4.78 (dt, *J* = 15.6, 9.2 Hz, 1H), 4.44 (d, *J* = 5.7 Hz, 2H), 4.02 – 3.83 (m, 6H), 3.57 – 3.49 (m, 2H), 3.36 – 3.33 (m, 1H), 2.20 – 2.11 (m, 4H), 1.39 (s, 9H); LRMS (ESI): *m/z* 680 $[M+H]^+$.

***N*-((3-(3-Carbamothioylphenyl)-1-(1-((trifluoromethyl)sulfonyl)piperidin-4-yl)-1*H*-indol-6-yl)methyl)azetidine-3-carboxamide hydrochloride salt (7, AS-85).** To a mixture of **38** (2000 mg, 2.942 mmol) in DCM (20 mL) was added TFA (6 mL) at RT. The reaction mixture was stirred at RT for 3.5 h. After concentration of mixture, the crude was purified by reverse phase column chromatography (C18, 0~47% MeOH in water (0.1% HCl)) to give the title compound (**7**, **AS-85**, 1080 mg, 1.756 mmol, 60% yield) as a pale yellow solid; ^{1}H NMR (600 MHz, DMSO-d_6) δ 9.89 (br. s., 1H), 9.54 (br. s., 1H), 9.10 (br. s., 1H), 8.85 (br. s., 1H), 8.65 (t, *J* = 5.32 Hz, 1H), 8.19 (s,

1H), 8.01 (s, 1H), 7.89 (d, *J* = 8.07 Hz, 1H), 7.81 (d, *J* = 7.70 Hz, 1H), 7.73 (d, *J* = 8.07 Hz, 1H), 7.58 (s, 1H), 7.46 (t, *J* = 7.70 Hz, 1H), 7.11 (d, *J* = 8.07 Hz, 1H), 4.75 - 4.86 (m, 1H), 4.45 (d, *J* = 5.50 Hz, 2H), 3.95 - 4.08 (m, 6H), 3.62 - 3.68 (m, 1H), 3.50 - 3.55 (m, 2H),2.07 - 2.21 (m, 4H); ^{13}C NMR (151 MHz, DMSO-d$_6$) δ 200.5, 169.5, 140.2, 136.2, 135.0, 132.4, 128.9, 128.3, 125.2, 124.3, 123.9, 120.8, 120.3, 119.4, 118.7, 115.1, 109.4, 50.8, 47.5, 46.1, 43.1, 34.9, 31.8; ESI HRMS (m/z): $[M+H]^+$ calcd. for $C_{26}H_{28}F_3N_5O_3S_2$ 580.1658; found 580.1659.

***N*-((3-(3-Carbamothioylphenyl)-1-(1-((trifluoromethyl)sulfonyl)piperidin-4-yl)-1*H*-indol-6-yl)methyl)-1-methylazetidine-3-carboxamide hydrochloride (8, AS-99).** To a stirred solution of **7** (480 mg, 0.779 mmol) and triethylamine (217 µL, 1.557 mmol) in DCM (5 mL) and MeOH (1 mL), was added STAB (124 mg, 0.584 mmol) in one portion at 0 °C. Formaldehyde (37% solution, 1168 µL, 15.582 mmol) was then added dropwise to the mixture at 0 °C. The reaction mixture was then allowed to warm to RT over 3 h. Saturated NH_4Cl aq. solution was added to quench the reaction. The organic layer was separated and concentrated *in vacuo*. The crude was purified by column chromatography on silica gel (0~20% MeOH (5% ammonia solution) in DCM) to give the title compound (**8**, **AS-99**, 300 mg, 0.476 mmol, 61% yield); ^{1}H NMR (600 MHz, DMSO-d_6) δ 9.89 (s, 1H), 9.54 (s, 1H), 8.53 (t, *J* = 5.8 Hz, 1H), 8.19 (t, *J* = 1.9 Hz, 1H), 8.00 (s, 1H), 7.89 (d, *J* = 8.2 Hz, 1H), 7.84 – 7.78 (m, 1H), 7.74 (ddd, *J* = 7.8, 1.9, 1.1 Hz, 1H), 7.56 (d, *J* = 1.5 Hz, 1H), 7.46 (t, *J* = 7.8 Hz, 1H), 7.11 (dd, *J* = 8.3, 1.4 Hz, 1H), 4.79 (dq, *J* = 11.1, 7.5, 5.6 Hz, 1H), 4.44 (d, *J* = 5.7 Hz, 2H), 4.05 – 3.98 (m, 2H), 3.83 (t, *J* = 8.6 Hz, 2H), 3.70 – 3.61 (m, 2H), 3.58 – 3.49 (m, 2H), 3.43 – 3.37 (m, 1H), 2.53 (s, 3H), 2.15 (tq, *J* = 11.0, 5.1, 4.5 Hz, 4H); ^{13}C NMR (151 MHz, CD_3OD) δ 204.39, 173.77, 141.82, 138.10, 137.05, 133.83, 130.94, 129.54, 127.37, 126.78, 125.21, 124.04, 121.64, 121.03, 117.80, 110.41, 74.53, 59.34, 53.29, 47.54, 45.09 (d, *J* = 14.4 Hz), 35.82, 33.42; ESI HRMS (m/z): $[M+H]^+$ calcd. for $C_{27}H_{30}F_3N_5O_3S_2$ 594.1815; found 594.1818.

Synthesis of 9 (AS-nc)

Synthetic pathway to prepare **9 (AS-nc)**.

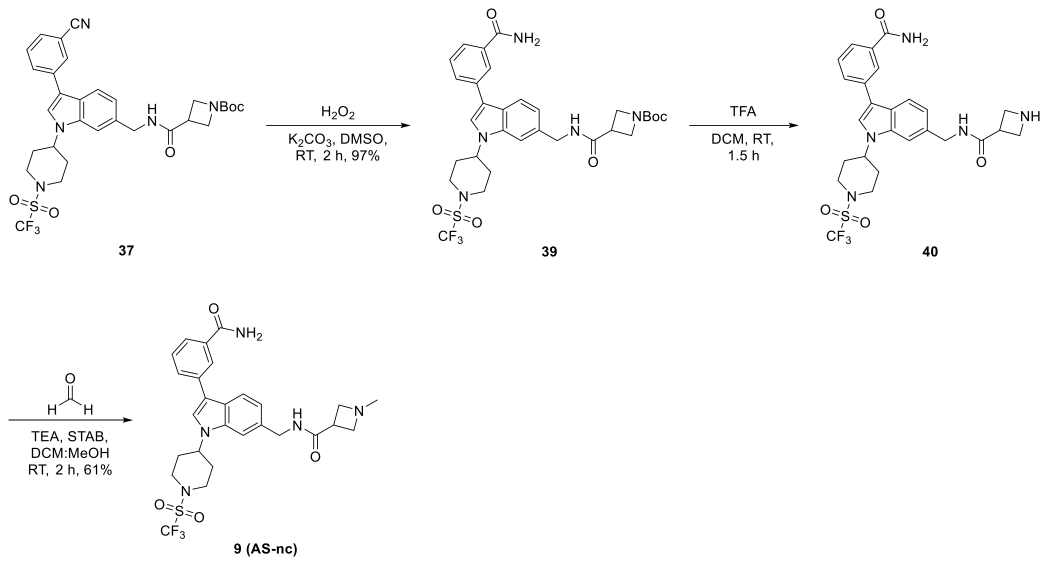

***tert*-Butyl 3-(((3-(3-carbamoylphenyl)-1-(1-((trifluoromethyl)sulfonyl)piperidin-4-yl)-1*H*-indol-6-yl)methyl)carbamoyl)azetidine-1-carboxylate (39).** To a solution of **37** (200 mg, 0.310 mmol) in DMSO (4 mL) was added K_2CO_3 (214 mg, 1.548 mmol) and H_2O_2 (30% solution, 200 µL, 1.958 mmol) at 0 °C. The mixture was stirred and slowly warmed to RT over 2 h. Saturated sodium thiosulfate aq. solution (10 mL) was added dropwise to the mixture to quench the reaction. The product was extracted by EtOAc (2 × 20 mL). The organic phase was separated and evaporated. The crude was purified by column chromatography on silica gel (0~9% MeOH in DCM) to give the title compound (**39**, 200 mg, 0.301 mmol, 97% yield) as a yellow oil. ^{1}H NMR (600 MHz, DMSO-d_6) δ 8.47 (t, *J* = 5.8 Hz, 1H), 8.16 (d, *J* = 1.8 Hz, 1H), 7.99 (d, *J* = 9.2 Hz, 2H), 7.89 (d, *J* = 8.2 Hz, 1H), 7.87 – 7.83 (m, 1H), 7.72 (d, *J* = 7.7 Hz, 1H), 7.54 (s, 1H), 7.50 (t, *J* = 7.7 Hz, 1H), 7.37 (s, 1H), 7.10 (dd, *J* = 8.2, 1.4 Hz, 1H), 4.79 (dd, *J* = 14.9, 8.9 Hz, 1H), 4.43 (d, *J* = 5.7 Hz, 2H), 4.04 – 3.86 (m, 7H), 3.57 – 3.48 (m, 2H), 2.16 (d, *J* = 8.8 Hz, 4H), 1.38 (s, 9H).

***N*-((3-(3-Carbamoylphenyl)-1-(1-((trifluoromethyl)sulfonyl)piperidin-4-yl)-1*H*-indol-6-yl)methyl)azetidine-3-carboxamide (40).** A mixture of **39** (200 mg, 0.301 mmol) in DCM (3 mL) was added TFA (1 mL). The reaction mixture was stirred at RT for another 1.5 h. After concentration of the mixture, the crude used in the next step directly. ^{1}H NMR (600 MHz, DMSO-d_6) δ 8.66 (s, 1H), 8.60 (t, *J* = 5.7 Hz, 1H), 8.17 (t, *J* = 1.8 Hz, 1H), 8.01 (s, 1H), 7.99 (s, 1H), 7.89 (d, *J* = 8.2 Hz, 1H), 7.84 (dt, *J* = 7.7, 1.3 Hz, 1H), 7.73 (dt, *J* = 7.8, 1.3 Hz, 1H), 7.57 (d, *J* = 1.4 Hz, 1H), 7.51 (t, *J* = 7.7 Hz, 1H), 7.38 (s, 1H), 7.12 (dd, *J* = 8.2, 1.4 Hz, 1H), 4.78 (p, *J* = 8.0 Hz,

1H), 4.46 (d, *J* = 5.7 Hz, 2H), 4.05 – 4.00 (m, 6H), 3.65 – 3.57 (m, 1H), 3.52 (dt, *J* = 13.6, 7.5 Hz, 2H), 2.16 (q, *J* = 5.7 Hz, 4H).

***N*-((3-(3-Carbamoylphenyl)-1-(1-((trifluoromethyl)sulfonyl)piperidin-4-yl)-1*H*-indol-6-yl)methyl)-1-methylazetidine-3-carboxamide (9, AS-nc).** To a stirring solution of **40** (170 mg, 0.301 mmol) and TEA (85 µL, 0.610 mmol) in a mix solution of DCM (6 mL) and MeOH (2 mL), was added STAB (192 mg, 0.906 mmol) in one portion. Formaldehyde (37% solution, 1200 µL, 16.015 mmol) was then added dropwise at 0 °C. The reaction mixture was then allowed to warm to RT over 2 h. Saturated NH_4Cl aq. solution was added to quench the reaction. The organic layer was separated and concentrated *in vacuo*. The crude was purified by column chromatography on silica gel (0~20% MeOH (5% ammonia solution) in DCM) to give the title compound (**9**, **AS-nc**, 106 mg, 0.184 mmol, 61% yield) as a pale yellow solid. ^{1}H NMR (600 MHz, CD_3CN) δ 8.11 (t, *J* = 1.8 Hz, 1H), 7.88 (d, *J* = 8.3 Hz, 1H), 7.85 (ddd, *J* = 7.7, 1.8, 1.1 Hz, 1H), 7.70 (ddd, *J* = 7.8, 1.8, 1.1 Hz, 1H), 7.67 (s, 1H), 7.52 (t, *J* = 7.7 Hz, 1H), 7.47 (d, *J* = 1.4 Hz, 1H), 7.12 (dd, *J* = 8.3, 1.4 Hz, 1H), 7.00 (s, 1H), 6.83 (s, 1H), 6.02 (s, 1H), 4.65 (dd, *J* = 11.8, 4.0 Hz, 1H), 4.50 (d, *J* = 5.9 Hz, 2H), 4.09 (dt, *J* = 13.5, 2.4 Hz, 2H), 3.53 (t, *J* = 8.0 Hz, 2H), 3.44 (t, *J* = 12.8 Hz, 2H), 3.36 (dd, *J* = 7.9, 6.5 Hz, 2H), 3.28 (s, 3H), 3.19 (tt, *J* = 8.0, 6.6 Hz, 1H), 2.24 – 2.19 (m, 2H), 2.14 (qd, *J* = 12.4, 4.3 Hz, 2H). ^{13}C NMR (151 MHz, CD_3CN) δ 169.34, 137.25, 136.47, 135.29, 134.13, 130.47, 129.56, 126.33, 125.54, 125.32, 123.88, 121.01, 120.32, 116.56, 109.63, 58.96, 52.31, 49.52, 46.91, 44.80, 35.59, 32.53; ESI HRMS (m/z): $[M+H]^+$ calcd. for $C_{27}H_{30}F_3N_5O_4S$ 578.2043; found 578.2043.

Supplementary References

1. Engelhardt, H. et al. Start Selective and Rigidify: The Discovery Path toward a Next Generation of EGFR Tyrosine Kinase Inhibitors. *J Med Chem* **62**, 10272-10293 (2019).

2. Hopkins, A.L., Keseru, G.M., Leeson, P.D., Rees, D.C. & Reynolds, C.H. The role of ligand efficiency metrics in drug discovery. *Nat Rev Drug Discov* **13**, 105-21 (2014).
