# Supplementary material for: Discovery of first-in-class inhibitors of ASH1L histone methyltransferase with anti-leukemic activity
Source: Nat Commun. 2021 May 14;12:2792. doi: 10.1038/s41467-021-23152-6 (PMC8121805; doi:10.1038/s41467-021-23152-6)
Supplement: Supplementary file 2 — Reporting Summary [file 41467_2021_23152_MOESM2_ESM.pdf]

Corresponding author(s): Jolanta Grembecka, Tomasz Cierpicki

Last updated by author(s): Jan 30, 2021

Reporting Summary

Nature Research wishes to improve the reproducibility of the work that we publish. This form provides structure for consistency and transparency in reporting. For further information on Nature Research policies, see Authors & Referees and the Editorial Policy Checklist.

Statistics

For all statistical analyses, confirm that the following items are present in the figure legend, table legend, main text, or Methods section.

n/a	Confirmed	
☐	☒	The exact sample size (*n*) for each experimental group/condition, given as a discrete number and unit of measurement
☐	☒	A statement on whether measurements were taken from distinct samples or whether the same sample was measured repeatedly
☐	☒	The statistical test(s) used AND whether they are one- or two-sided<br>*Only common tests should be described solely by name; describe more complex techniques in the Methods section.*
☒	☐	A description of all covariates tested
☒	☐	A description of any assumptions or corrections, such as tests of normality and adjustment for multiple comparisons
☐	☒	A full description of the statistical parameters including central tendency (e.g. means) or other basic estimates (e.g. regression coefficient) AND variation (e.g. standard deviation) or associated estimates of uncertainty (e.g. confidence intervals)
☐	☒	For null hypothesis testing, the test statistic (e.g. *F*, *t*, *r*) with confidence intervals, effect sizes, degrees of freedom and *P* value noted<br>*Give P values as exact values whenever suitable.*
☒	☐	For Bayesian analysis, information on the choice of priors and Markov chain Monte Carlo settings
☒	☐	For hierarchical and complex designs, identification of the appropriate level for tests and full reporting of outcomes
☒	☐	Estimates of effect sizes (e.g. Cohen's *d*, Pearson's *r*), indicating how they were calculated

Our web collection on statistics for biologists contains articles on many of the points above.

Software and code

Policy information about availability of computer code

Data collection

NMR data were collected using 600 MHz Bruker Avance III spectrometer running Topspin 2.1.
ITC data were collected at the VP-ITC titration calorimetric system (MicroCal)
X-ray data were collected at the 21ID-F, 21ID-G beamlines at the Advanced Photon Source.
Flow cytometry experiments were performed on LSRII (BD Biosciences)
RNA- sequencing and CUT&RUN profiling: Illumina NovaSeq (S4) Sequencer (Illumina)
Mouse image data were collected on in Vivo Imaging System BLI (IVIS, 200)

Data analysis

All software used in current study were commercially or publicly available and are described in the Methods section. Data displayed in graphs were generated and analyzed using GraphPad Prism 8.0.0 (GraphPadSoftware Inc.).
NMR data were analyzed using NMRPipe v3.0 and Sparky v3.113 programs.
ITC experiments were analyzed using Origin 7.0 software (Originlab).
X-ray diffraction data were processed using HKL2000 v.720. Crystal structures were determined using MOLREP v.11, WinCoot v. v.0.8.9.2, PHASER-MR v.1.17.1, PHENIX v.1.17.1 and MolProbity v.4.02b-467 (details are provided in the Methods section).
Flow cytometry data were analyzed with FlowJo v.10.6.0 software (Tree Star, Inc.)
For analysis of RNA-seq data Illumina's bcl2fastq2 (v2.17), Tophat2 (2.1.1), SAMtools (v1.5), Cufflinks (v2.2.1), Cuffdiff242, “ggplot2” R package, Cuffdiff2, “gplots” package and “fgsea” were used. Details are provided in the methods section.
For analysis of CUT&RUN data Bowtie2 (v2-2.2.4), SAMtools (v1.5), BEDTools v2.28.0, MACS (v 1.4.2), IGV (v2.3), MACS2 (V2.1.1), Homer (v4.10), deepTools (v2.5.7), ggpubr (v0.4.0) were used. Details are provided in the methods section.
PK parameters were calculated using WinNonlin® version 3.2

For manuscripts utilizing custom algorithms or software that are central to the research but not yet described in published literature, software must be made available to editors/reviewers. We strongly encourage code deposition in a community repository (e.g. GitHub). See the Nature Research guidelines for submitting code & software for further information.

mouse image data were analyzed in software IVIS Imaging Systems 200

Data

Policy information about availability of data

All manuscripts must include a data availability statement. This statement should provide the following information, where applicable:

- Accession codes, unique identifiers, or web links for publicly available datasets
- A list of figures that have associated raw data
- A description of any restrictions on data availability

The coordinates for ASH1L-AS-5 and ASH1L-AS-85 complexes were deposited in PDB under PDB codes 6X0P (https://www.rcsb.org/structure/unreleased/6X0P) and 6WZW (https://www.rcsb.org/structure/unreleased/6WZW), respectively. RNA-seq and CUT&RUN data were deposited to GEO under the accession number GSE150087 (SubSeries numbers: GSE150085 and GSE150086 for the RNA-seq and CUT&RUN data, respectively; https://www.ncbi.nlm.nih.gov/geo/query/acc.cgi?acc=GSE150087).

Field-specific reporting

Please select the one below that is the best fit for your research. If you are not sure, read the appropriate sections before making your selection.

☒ Life sciences ☐ Behavioural & social sciences ☐ Ecological, evolutionary & environmental sciences

For a reference copy of the document with all sections, see nature.com/documents/nr-reporting-summary-flat.pdf

Life sciences study design

All studies must disclose on these points even when the disclosure is negative.

Sample size	Sample sizes were not predetermined using statistical software
Data exclusions	No data were excluded
Replication	Cell based experiments were performed at least twice with two to three technical replicates in each experiment, as indicated in the figure legends. All attempts were successful. RNA-Seq experiment was performed once with three technical replicates. CUT&RUN experiment was performed once with one replicate. In vivo study, control group had 6 mice, treatment group had 7 mice.
Randomization	Mice grouping was based on the bioluminescence level of individual mice. Experiments were not blinded or randomized.
Blinding	No blinding was used. Experimental results were obtained by automated methods (e.g. qRT-PCR, mTT read-out, etc.)

Reporting for specific materials, systems and methods

We require information from authors about some types of materials, experimental systems and methods used in many studies. Here, indicate whether each material, system or method listed is relevant to your study. If you are not sure if a list item applies to your research, read the appropriate section before selecting a response.

Materials & experimental systems

n/a	Involved in the study	
☐	☒	Antibodies
☐	☒	Eukaryotic cell lines
☒	☐	Palaeontology
☐	☒	Animals and other organisms
☒	☐	Human research participants
☒	☐	Clinical data

Methods

n/a	Involved in the study	
☒	☐	ChIP-seq
☐	☒	Flow cytometry
☒	☐	MRI-based neuroimaging

Antibodies

Antibodies used

FITC Annexin V Apoptosis Detection Kit I (BD 556547, BD Pharmingen™)
Zombie Aqua™ Fixable Viability Kit (Biolegend®, 423102;
anti-human CD11b (Biolegend®, 982606, ICRF44, and BD BioLegend, 101224) anti-H3K36me2 (Active Motif, 39255)
anti-hCD45-BV421 (BD Bioscience, 563879, HI30)
anti-mouse CD11b (Biolegend, #101208, M1/70),
anti-mouse Gr-1 (BioLegend, #108433, RB6-8C5),
anti-mouse B220 (BioLegend, #103224, RA3-6B2),
anti-mouse CD19 (BioLegend, #115520, 6D5),
anti-mouse CD3 (BioLegend, #109211, H57-597),
anti-mouse Zombie Aqua-dead cells (BioLegend, #423101).

Validation	All antibodies were obtained commercially and had been validated by the companies. The information can be accessed through the manufacturer's websites using the catalog numbers provided in the Method section.

Eukaryotic cell lines

Policy information about cell lines

Cell line source(s)	MV4;11, K562, KOPN8 and RS4;11 cell lines were obtained from ATCC. MOLM13 and SET2 cell lines were obtained from DSMZ. MLL-AF9, MLL-AF6, E2A-HLF and HM-2 cells were generated by transforming murine progenitor cells with indicated oncogenes (details are provided in the manuscript/ previously described); human CD34+ hematopoietic cord blood cells were purchased from Stem Cell Technologies
Authentication	Authentication of human cells was performed by supplying vendors (ATCC using DNA fingerprinting and DSMZ by STR profiling as described on ATCC and DSMZ web pages). No additional authentication of these cell lines was performed. Murine cell lines transformed with oncogenes were confirmed by colony formation.
Mycoplasma contamination	All cell isolates tested negative for mycoplasma.
Commonly misidentified lines (See ICLAC register)	No commonly misidentified cell lines were used in the present study.

Animals and other organisms

Policy information about studies involving animals; ARRIVE guidelines recommended for reporting animal research

Laboratory animals	NSG mice, 8-10 weeks old, female; C57BL/6 mice, 8-10 weeks old, female
Wild animals	This study did not involve wild animals.
Field-collected samples	This study did not involve field-collected animals.
Ethics oversight	University of Michigan Committee on Use and Care of Animals and Unit for Laboratory Animal Medicine

Note that full information on the approval of the study protocol must also be provided in the manuscript.

Flow Cytometry

Plots

Confirm that:

☒ The axis labels state the marker and fluorochrome used (e.g. CD4-FITC).

☒ The axis scales are clearly visible. Include numbers along axes only for bottom left plot of group (a 'group' is an analysis of identical markers).

☒ All plots are contour plots with outliers or pseudocolor plots.

☒ A numerical value for number of cells or percentage (with statistics) is provided.

Methodology

Sample preparation	For the cells isolated from the mice transplanted with MV4-11, cells from peripheral blood or spleen were isolated and suspended in PBS with 1% FBS for flow cytometry analysis. Red blood cells were lysed with ACK (Lonza). Cells from human cell lines were washed in PBS with 1% FBS (flow buffer), stained 30 minutes, washed twice with flow buffer, and used for flow cytometry analysis.
Instrument	FACSCelesta flow cytometer, Becton-Dickinson, FACSCelesta
Software	The data was collected using BD FACSDiva version 8, and data was analyzed using Flowjo, version 10.6.0
Cell population abundance	No cell sorting was performed
Gating strategy	FSC/SSC were used for gating out the debris, the FSC A/FSC W were used for single cell selection. For the hCD45 gating in the samples derived from mice, the non-stained samples served as a negative control. For gating in experiments using human cell lines, the non-stained samples were used as a reference. Gating strategy is presented in Supplementary Information; Supplementary Fig. 8c, 8d; Supplementary Fig. 11e and Supplementary Fig. 12c..

☒ Tick this box to confirm that a figure exemplifying the gating strategy is provided in the Supplementary Information.
